# Transcriptomic insights on the virulence-controlling CsrA, BadR, RpoN, and RpoS regulatory networks in the Lyme disease spirochete

**DOI:** 10.1371/journal.pone.0203286

**Published:** 2018-08-30

**Authors:** William K. Arnold, Christina R. Savage, Kathryn G. Lethbridge, Trever C. Smith, Catherine A. Brissette, Janakiram Seshu, Brian Stevenson

**Affiliations:** 1 Department of Microbiology, Immunology and Molecular Genetics, University of Kentucky School of Medicine, Lexington, KY, United States of America; 2 Department of Biology, University of Texas at San Antonio, San Antonio, TX, United States of America; 3 Department of Biomedical Sciences, University of North Dakota School of Medicine and Health Sciences, Grand Forks, ND, United States of America; 4 Department of Entomology, University of Kentucky, Lexington, KY, United States of America; University of Toledo College of Medicine and Life Sciences, UNITED STATES

## Abstract

*Borrelia burgdorferi*, the causative agent of Lyme disease, survives in nature through a cycle that alternates between ticks and vertebrates. To facilitate this defined lifestyle, *B*. *burgdorferi* has evolved a gene regulatory network that ensures transmission between those hosts, along with specific adaptations to niches within each host. Several regulatory proteins are known to be essential for the bacterium to complete these critical tasks, but interactions between regulators had not previously been investigated in detail, due to experimental uses of different strain backgrounds and growth conditions. To address that deficit in knowledge, the transcriptomic impacts of four critical regulatory proteins were examined in a uniform strain background. Pairs of mutants and their wild-type parent were grown simultaneously under a single, specific culture condition, permitting direct comparisons between the mutant strains. Transcriptomic analyses were strand-specific, and assayed both coding and noncoding RNAs. Intersection analyses identified regulatory overlaps between regulons, including transcripts involved in carbohydrate and polyamine metabolism. In addition, it was found that transcriptional units such as *ospC* and *dbpBA*, which were previously observed to be affected by alternative sigma factors, are transcribed by RNA polymerase using the housekeeping sigma factor, RpoD.

## Introduction

*Borrelia burgdorferi*, the bacterium that causes Lyme disease, is an obligately parasitic spirochete whose enzootic cycle alternates between vertebrates and *Ixodes* spp. ticks. Survival of *B*. *burgdorferi* in nature requires that the spirochete accurately control production of proteins and other factors necessary for each aspect of its cycle. A number of *B*. *burgdorferi* factors have been identified that are critical for bacterial survival in nature, and have been observed in culture to control production of borrelial RNAs, proteins, and other components [1–3]. All evidence indicates that regulatory networks that operate in cultured bacteria are also functional during the bacteria’s vertebrate-tick infectious cycle [1–3]. Insights on *B*. *burgdorferi* regulatory networks have been obtained through transcriptome or proteome comparisons between mutant and wild-type bacteria. However, all prior studies examined only a single mutant and its parent. Due to variations in culture conditions and/or strain backgrounds, results of such studies cannot be directly compared with each other. To permit comparisons of mutants with each other, and thereby identify regulatory network overlaps, the present studies examined wild-type and several congenic mutant strains, all cultured under the same conditions. Four regulatory factors were examined that have been shown to be, or hypothesized to be, critical for *B*. *burgdorferi*’s transmission between feeding ticks and vertebrate hosts [1–3].

An alternative sigma factor, RpoS, is required for maximal expression of a regulon that is important for transmission from ticks into vertebrates, and during vertebrate infection. Previous studies of the RpoS regulon were focused on bacteria cultured under conditions that induce high-level expression of RpoS, such that comparisons of wild-type and Δ*rpoS* strains readily revealed differences in expression levels of RpoS-targeted transcripts [4–7]. A caveat of examining bacteria under such conditions is that low-level expression of transcripts in an *rpoS* mutant may be misinterpreted as absence of those transcripts, especially when using techniques with limited sensitivity, such as immunoblotting or arrays. Using RpoS-inducing conditions may also complicate studies of other regulatory factors that function both through and independently of RpoS, since a mutant’s impact on RpoS might be misinterpreted as evidence that all effects of the other regulator are mediated through RpoS.

Two transcriptional promoters have previously been described for *rpoS*, one of which is dependent upon another alternative sigma, RpoN [5, 8–10], and a second that appears to be dependent upon the housekeeping sigma, RpoD [11]. For that reason, an *rpoN* mutant was included in these analyses. Three DNA-binding proteins, including BadR, bind to sites 5’ of *rpoS*, and have been reported to affect the transcription of *rpoS* under certain conditions [11–17].

BadR is a ROK (repressor of kinase) type of DNA-binding protein. It was originally annotated as a putative xylose-responsive repressor [16, 18]. That hypothetical function is likely incorrect, as *B*. *burgdorferi* cannot utilize xylose as an energy source [19]. Prior studies found that BadR binds to DNA 5’ of *rpoS*, and a *badR* mutant exhibited altered expression of *rpoS* under a tested culture condition [16, 17]. Furthermore, BadR was also shown to bind DNA 5’ of *bosR*, which encodes another DNA-binding protein that binds 5’ of *rpoS* [17]. A previous array analysis of a cultured *badR* mutant detected significantly altered expression of over 200 transcripts, including numerous mRNAs of proteins that are important for mammalian infection [16, 17]. Consistent with those findings, *badR* mutants are not able to colonize mice. As with other ROK-type DNA-binding proteins, binding of recombinant BadR to DNA is modulated by certain phosphorylated carbohydrates [16].

CsrA (Carbon storage regulator A) is an RNA-binding protein that, in other organisms, regulates diverse cellular processes including its namesake process, carbon storage [20]. Homologues have roles in regulation of virulence in several pathogenic bacterial species [21, 22]. Previous studies on the *B*. *burgdorferi* CsrA homologue revealed seemingly contradictory results. Several studies observed that *csrA* mutants exhibited altered expression of lipoproteins, motility, and cell shape, and were unable to infect mice [23–27]. In contrast, another study did not observe those changes in protein expression or infectivity [28]. The basis of those different results remains to be determined. Consistent with the former, the present studies observed significant changes to numerous transcripts in a *csrA* mutant, supporting the hypothesis that CsrA is a regulator of *B*. *burgdorferi* physiology.

In this study, a comprehensive transcriptomic approach was undertaken to identify transcripts that were significantly affected in bacteria deleted of either *rpoS*, *rpoN*, *badR*, or *csrA*. Strand-specific RNA sequencing (RNA-Seq) was used, permitting global analyses of the coding and noncoding transcriptomes. Those data were compared with results of their wild-type parental strain, to identify components of the CsrA, BadR, RpoS, and RpoN regulons that were affected under a single, uniform condition. Points were identified where those regulons intersect. The culture conditions used for these studies did not induce high-level expression of *rpoS*, the result of which provided substantial new insights on pathways that control borrelial gene expression. For example, CsrA was found to function independently of RpoS to exert substantial effects on numerous transcripts, and some transcriptional units that had previously been hypothesized to require RpoS for transcription, such as *ospC* and *dbpBA*, were found to be transcribed using the “housekeeping” sigma factor, RpoD.

## Materials and methods

### Bacteria and culture conditions

All studies described were performed using the *B*. *burgdorferi* strain B31-A3 and direct derivatives. B31-A3 is a clonal derivative of the type strain B31 [29, 30]. B31-A3 contains the full complement of naturally-occurring DNA elements identified in the sequenced culture of strain B31 with the exception of cp9 [18, 31]. Absence of cp9 does not have any detectable effects on infectivity or gene expression [29, 32, 33]. Generation and validation of each of the four mutations in the B31-A3 background has been described previously [8, 16, 27, 29]. Prior to RNA-Seq analyses, all strains were assessed for the presence of the full repertoire of natural DNA elements by multiplex PCR [34]. The *badR* and *rpoN* mutants had apparently lost lp21 during production or subsequent cultivation. lp21 contains a long stretch of untranscribed, repetitive DNA along with ORFs that are involved in maintenance and partitioning, and lp21 is not known to play a role in infection processes [18, 30, 32]. All other naturally-occurring plasmids were retained in all cultures of the strains.

Cultures and harvesting of bacteria were performed essentially as described previously [30]. *B*. *burgdorferi* were cultured in Barbour-Stoenner-Kelly II (BSK-II) liquid medium [35]. All strains were grown as at least three distinct cultures. Briefly, 5 ml of medium was inoculated with a 1:100 dilution of bacteria from frozen glycerol stocks, then incubated at 34°C. Previous studies have demonstrated that the inoculation from -80°C to warmer media conditions induces substantial changes in transcript and protein levels [36] which can confound studies of gene regulation. To avoid those effects, the initial 34°C cultures were grown until cell densities reached mid exponential phase (~1x10^7^ bacteria/ml). Cultures were then diluted into 10 ml of fresh BSK-II to a final density of 1x10^5^ bacteria/ml, and again incubated at 34°C. All cultures grew with essentially identical division rates. When cultures reached mid-exponential phase (1x10^7^ bacteria/ml), bacteria were harvested by centrifugation at 8200xG for 30 minutes at 4°C. Supernatants were removed and the cell pellets immediately resuspended in 1 ml of pre-warmed (60°C) TRIzol (Thermo-Fisher, Waltham, MA). Cell suspensions were stored until use at -80°C.

### RNA isolation and library construction

RNA was isolated and its integrity validated essentially as described previously [30]. Briefly, RNA was isolated from 500 μl of the above-described cell suspensions using the Zymo RNA Direct-Zol miniprep kit (Zymo, CA USA). RNA was eluted from the column with 35 μl RNase-free water and stored at -80C. Yield and integrity were examined using a Bioanalyzer with the RNA 6000 Nano kit (Agilent, CA USA). Electropherograms were examined to ensure that RNA was intact and all samples used for library construction had RIN scores >9. RNA concentration was further determined using a Nanodrop 2000 spectrophotometer (Thermo-Fisher, Waltham, MA).

Illumina cDNA libraries were generated using the RNAtag-seq protocol as described previously [30, 37]. Briefly, 840 ng of total RNA was fragmented, dephosphorylated, and ligated to DNA adapters carrying 5’-AN8-3’ barcodes with a 5’ phosphate and a 3’ blocking group. Samples bearing unique barcoded RNAs were pooled and depleted of rRNA using the RiboZero Bacterial Gold rRNA depletion kit (Illumina, CA USA). These pools of barcoded RNAs were converted to Illumina cDNA libraries and sequenced in paired end mode for 75 cycles on the Illumina Nextseq 500 platform (Illumina, San Diego, CA).

### RNA-Seq data analysis

As previously described [30, 38], reads corresponding to each particular sample were deconvoluted, based on their associated barcode. Up to 1 mismatch in the barcode was allowed, with the caveat that it did not result in assignment to multiple barcodes. Multiplexing barcodes were trimmed using in house scripts [30]. The expected read length following removal of indexing barcodes was 33bp and we attained an average read length of 32.5 bp. Quality of reads was assessed using FastQC (v0.11.5) (http://www.bioinformatics.babraham.ac.uk/projects/fastqc). De-convoluted reads were trimmed using Trimmomatic v0.36 [39] to remove low quality reads, trim low quality bases from the ends of reads, and to trim any reads in which both pairs did not have a length of at least 25 bases. A custom transcriptome (multi-FASTA) was created by merging the curated *B*. *burgdorferi* B31 coding sequences (NCBI Assembly ASM868v2_CDS as of 5/1/17) FASTA with a multi-FASTA of all ribosomal and tRNAs and a multi-FASTA containing a set of recently identified putative ncRNAs[30]. The custom index is available on Figshare (see below). The transcriptome was indexed using the Salmon-index function set for quasi mapping with default settings and auto library detection (v0.8.2) [40]. Mapping and counting was conducted using Salmon (v.0.8.2) in quasi mode with seqBias and GCbias flags activated. The *B*. *burgdorferi* genome contains several regions of high similarity encoded on the plasmids that have confounded both transcript quantification and genome assembly in the past [18, 31, 41, 42]. Salmon utilizes a probabilistic model to estimate the true mapping location for ambiguously-mapped reads, providing increased accuracy of estimation of both identical sequences in different locations and of paralogous gene clusters [40, 43]. For the examination of read abundance surrounding the *rpoS* locus reads from each sample were aligned to the *B*. *burgdorferi* B31 genome sequence using BWA [18, 31, 44], and read abundance was examined using Artemis (Release 16.0) [45].

For logistical reasons, cultures of *csrA*, *badR*, and the wild-type parent were grown simultaneously, and *rpoS*, *rpoN*, and additional cultures of the wild-type parent were simultaneously grown at a later date. Batch effects are a well-known confounding variable in RNA-Seq experiments, and can often account for as much or more variability than the biological effect in question [46]. To account for this, data from each mutant were compared with its simultaneously-grown wild-type and other mutant strain. Results from each set of cultures clustered well by principal component analysis, whereas the two batches of wild-type bacteria were separate from each other, supporting our decision to compare mutants to wild-type samples only within batch (S1 Fig). Results from the *csrA*, *badR*, and the wild-type parent were compared with each other, and the *rpoS*, *rpoN*, and wild-type were compared separately.

Downstream data analysis (differential expression testing, plotting, significance filtering, and intersection identification) was performed in RStudio (1.0.143) (http://www.rstudio.com). Differential expression analyses were conducted using DESeq2 (v1.41.1) both with and without a Benjamini–Hochberg FDR correction set to alpha = .05 [47]. Thirty-nine transcripts had less than three total reads summed across all samples and were not tested. PCA and MA plots were generated using DESeq functions plotMA and plotPCA. Count data was extracted using the DESeq plotCounts function and replotting the data using ggplot2 [48]. Significance filtering was set at a padj value less than 0.05 and a log2FoldChange of greater than one. The availability of all code and reference data utilized in these studies is openly available and is described below in the Data Availability section.

These analyses used the ncRNA list and nomenclature of the first comprehensive analysis of the *B*. *burgdorferi* noncoding transcriptome [30]. A later study by other researchers used different criteria for calling putative ncRNAs, resulting in a somewhat different list [49, 50]. Although the later list was not used in the current analyses, our raw data are readily accessible to anyone who wishes to analyze them against those or other transcript sets (see Data Availability, below).

### Quantitative reverse transcription-PCR (qRT-PCR)

Purified RNAs from each of the above-described cultures was also assayed by qRT-PCR for comparison with RNA-Seq results. Approximately 1 μg of isolated RNA was treated with Turbo DNase I for 45 minutes to remove contaminating genomic DNA (Thermo-Fisher, Waltham, MA). Normalized amounts of RNA were converted to cDNA using SuperScript (BioRad, Hercules, CA). cDNAs were diluted 1:20 for use in qPCR. SYBER-Green based qPCR was performed essentially as described previously [30, 51] using a CFX96 Touch (BioRad, Hercules, CA). Briefly, 2 μl of cDNA was combined with 5 μl 2X iTaq qPCR Supermix (BioRad, Hercules, CA), 300 μM of appropriate oligonucleotide primer pairs (S1 Table), and nuclease free H_2_O to a final volume of 10 μl. Reactions were performed in technical triplicate. Cycling conditions consisted of an initial melt at 95°C for 2 minutes followed by 40 cycles of PCR with a 15 second melt at 95°C, a 15 second extension at 60°C and fluorescence detection. Melt curves were performed by increasing reaction temperatures in 0.5°C increments from 65°C to 95°C. Melt curves confirmed that each particular set of primers and template generated a single specific product. Transcripts were targeted that do not have associated antisense RNAs. Data from qRT-PCR were analyzed by the ΔΔCt method [52] normalized to *ftsK*, which has previously been shown to be stably expressed during a variety of different culture conditions [30].

### Quantitative PCR analyses of native plasmid lp28-4

Total DNA was isolated from all five strains. For each, qPCR was performed, targeting lp28-4 (primer pair qlp28-4F and qlp28-4R) and the *dnaA* gene at the chromosome’s center (primer pair qDnaAF and qDnaAR) (S1 Table). The relative abundance of each strain’s lp28-4 was normalized to its chromosome, using the ΔCt method.

## Results

### Deletion of *csrA* perturbs transcripts of genes associated with virulence and diverse cellular processes

**Fig 1:**
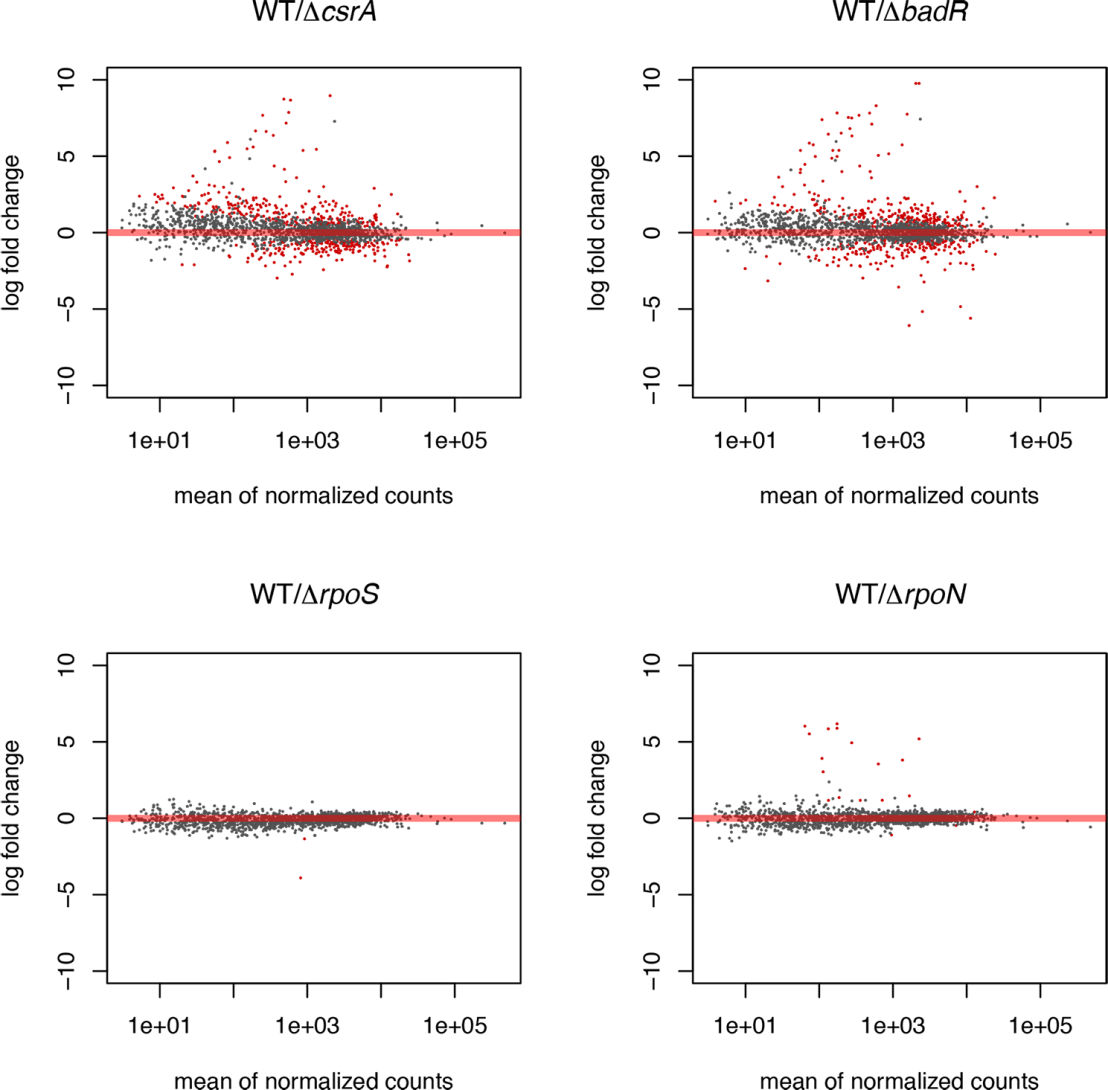
Log ratios of samples vs. mean abundance of transcripts. (A) *csrA* mutant compared to wild-type. (B) *badR* mutant compared to wild-type. (C) *rpoS* mutant compared to wild-type. (D) *rpoN* mutant compared to wild-type. Red points indicate transcripts which met the criteria of a log2 fold change >1 and an adjusted p-value (padj) < 0.05. The X-axis is given as mean normalized count across compared samples and the Y-axis as log2 fold change between conditions.

**Fig 2:**
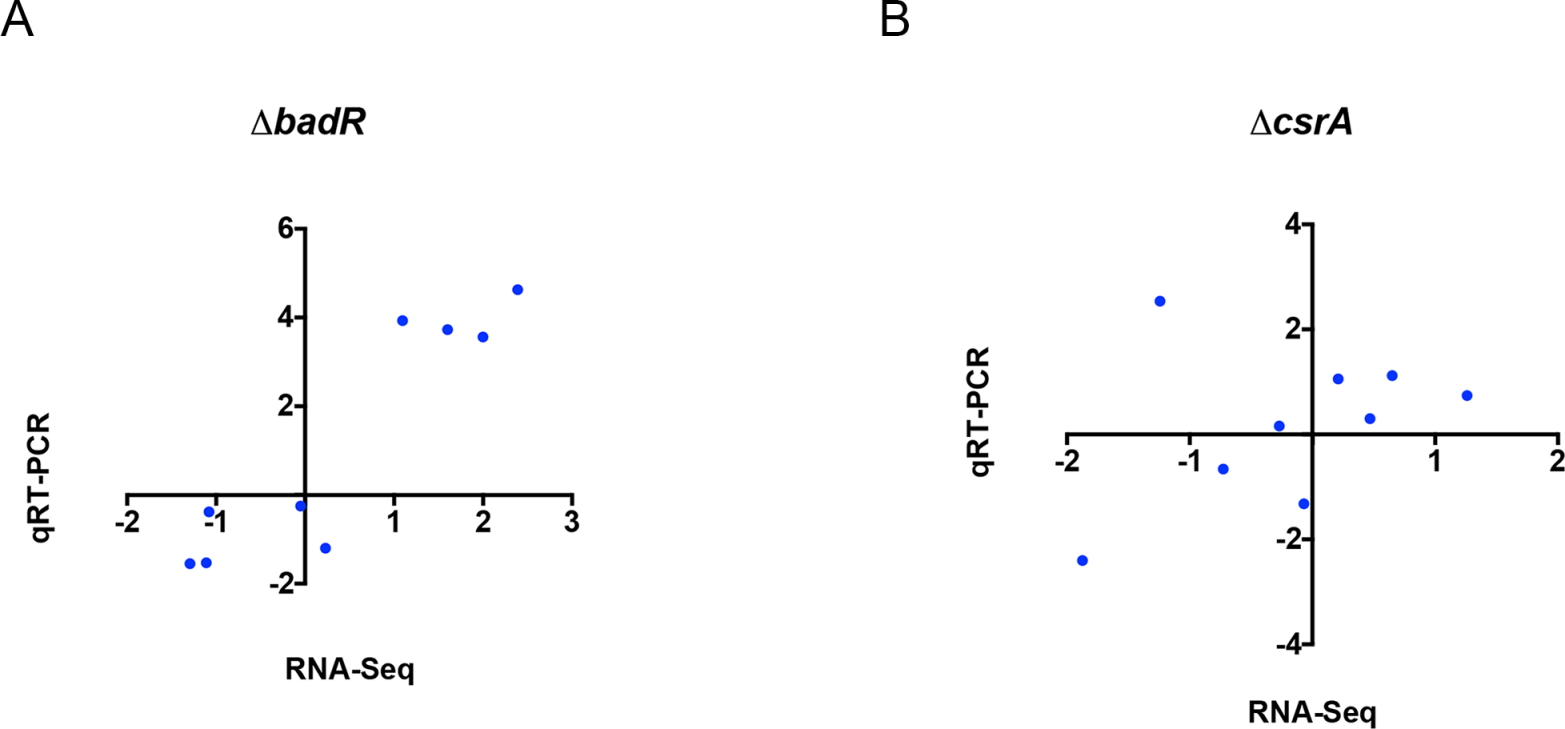
qRT-PCR of select transcripts in *csrA* and *badR* mutants. Total RNAs from the cultures used for RNA-Seq were converted to cDNA. qRT-PCR was performed on *cdaA*, *bosR*, *glpF*, *glpK*, *glpD*, *spoVG*, *bbk32*, *dbpA*, and *sodA*. Pearson correlations were calculated and plots were generated using GraphPad Prism 6. (A) Scatter plot comparing fold-change of each transcript as assayed by qRT-PCR (Y-axis) and RNA-Seq (X-axis) for Δ*badR* compared to the wild-type parent. The assayed transcripts were highly correlated, with a Pearson coefficient of 0.921, and two-tailed P-value of 0.0004. (B) Scatter plot comparing fold-change of each transcript as assayed by qRT-PCR (Y-axis) and RNA-Seq (X-axis) for Δ*csrA* compared to WT. The analyzed transcripts were not correlated, due to a single outlier, *sodA*, with a Pearson coefficient of 0.364. Also due to the outlier, the two-tailed P-value is 0.336. The reason for the sole inconsistency of *sodA* in Δ*csrA* is unclear. Ongoing investigations of *sodA* indicate that it is under complex regulation, including apparent post-transcriptional control by the BpuR RNA-binding protein (our unpublished results).

CsrA has been proposed to pre- and post-transcriptionally regulate a number of processes in *B*. *burgdorferi*, from flagellar assembly and motility to the expression of infection-associated proteins [23–26, 53]. To further investigate these hypotheses, RNA-Seq analyses were performed on a *csrA* null mutant, the first such global analysis of *B*. *burgdorferi* CsrA. We observed that 239 transcripts were significantly different between the *csrA* mutant and the wild-type parent (13.4% of the transcriptome) (Fig 1A, Table 1 and S2 and S5 Tables). Of the affected transcripts, 153 had reduced abundance and 86 had increased abundance in the mutant. Approximately two thirds (158 transcripts or 66%) of the differentially expressed (DE) transcripts consisted of ORF mRNAs [30]. The majority of DE transcripts (171/239 or 71.5%) were plasmid-encoded, and the majority of these were reduced in the mutant (116/169 or 68.6%). Importantly, deletion of *csrA* did not have significant effects on any of the other three regulatory proteins being studied, indicating that the observed effects were not due to CsrA working through BadR, RpoS, or RpoN. The RNA-Seq results were validated by performing qRT-PCR analyses of *cdaA*, *glpF*, *glpK*, *glpD*, *bosR*, *spoVG*, *bbk32*, *dbpA*, *and sodA* transcripts (Fig 2).

**Table 1:** Differentially expressed transcripts when comparing the csrA mutant to wild-type, listed in order of genome reference number. The included transcripts met the criteria of >1 log2 fold-change and an adjusted p-value (padj) when comparing the *csrA* mutant to wild-type. A total of 239 transcripts were differentially regulated, not including the mutated gene, by the mutation. The first column contains the CDS/custom transcript ID which is the transcript ID for all coding sequences obtained from the NCBI Gene file format file or the transcript ID given to ncRNAs. RefSeq entries are further separated by the character “_”. The first portion gives the genetic element from which it is derived, the second describes the type of element (CDS), the third provides RefSeq ID, and the fourth provides a number indicating the particular entries ordered number in the RefSeq entry. The second column is the gene information, for the ncRNAs it contains the location relative to other genes and for predicted or known genes it contains gene name. The remaining columns describe the various metrics of expression of each impacted transcript including, base mean (average library size normalized counts across all samples), log2FC (Fold change estimate), lfcSE (uncertainty of the log fold change estimate), stat (Wald statistic), pvalue, padj (pvalue following Benjamini-Hochberg adjustment). ORFs and ncRNAs are identified according to the names or numbers assigned to genes and transcripts by the initial genome sequencing of *B*. *burgdorferi* strain B31 [18, 31] or from our previous analyses of that strain’s ncRNA transcriptome [30].

RefSeq CDS/Custom Transcript ID	Gene Name	baseMean	log2FoldChange	lfcSE	stat	pvalue	padj
ncRNA0002	AI-(BB_0004,BB_0004/BB_0005)	3041.858416	-1.36714353	0.248794138	-5.495079342	3.91E-08	7.56E-07
ncRNA0003	AA-(BB_0005,BB_0006)	1243.576562	-1.545333022	0.311573702	-4.959767184	7.06E-07	1.03E-05
ncRNA0006	A-(BB_0013)	3033.894978	-1.608698814	0.339632615	-4.736585192	2.17E-06	2.74E-05
ncRNA0007	A-(BB_0014)	214.8526993	-1.215649307	0.481870969	-2.522769338	0.011643475	0.040193419
ncRNA0014	A-(BB_0084)	380.5460997	-1.357896884	0.388753058	-3.492954863	0.000477707	0.002884877
ncRNA0031	AA-(BB_0198,BB_0199)	400.7134034	-1.332232934	0.373955161	-3.562547258	0.000367274	0.002360254
ncRNA0035	A-(BB_0208)	510.9105757	-1.073378765	0.291108995	-3.687205759	0.00022673	0.001526171
ncRNA0037	A-(BB_0211)	1238.76926	-1.369299269	0.314591832	-4.352621806	1.35E-05	0.000134756
ncRNA0042	A-(BB_0240)	2021.458735	-1.879991865	0.349110627	-5.385089195	7.24E-08	1.33E-06
ncRNA0043	A-(BB_0244)	577.795707	-1.256653342	0.403868558	-3.111540418	0.00186114	0.009029975
ncRNA0050	AIA-(BB_0269,BB_0269/BB_0270,BB_0270)	1285.298201	-1.696511444	0.556125738	-3.050589691	0.002283925	0.010685504
ncRNA0057	A-(BB_0347)	34.51259723	-1.887737468	0.548599499	-3.441012019	0.000579543	0.003368469
ncRNA0063	A-(BB_0381)	391.0552137	-1.635832191	0.433065836	-3.777329118	0.000158519	0.001120158
ncRNA0070	A-(BB_0446)	529.3172145	-1.785175913	0.312674426	-5.709376155	1.13E-08	2.54E-07
ncRNA0071	A-(BB_0450)	65.28090439	-4.683065312	0.697311108	-6.715890878	1.87E-11	7.76E-10
ncRNA0072	A-(BB_0454)	396.651084	2.956000331	0.659455311	4.482487719	7.38E-06	8.16E-05
ncRNA0073	I-(BB_t06/BB_0461)	2784.933991	1.305078491	0.230041096	5.673240628	1.40E-08	3.10E-07
ncRNA0080	p-(BB_0522)	879.8130905	-1.668711458	0.235879187	-7.074432798	1.50E-12	6.73E-11
ncRNA0084	A-(BB_0581)	87.52936081	-1.86382887	0.465310745	-4.005557342	6.19E-05	0.000490079
ncRNA0087	A-(BB_0588)	1536.156421	-1.983017812	0.709181109	-2.796207891	0.005170613	0.020915805
ncRNA0099	A-(BB_0633)	150.8640122	-2.112142504	0.596785888	-3.539196463	0.000401347	0.002531459
ncRNA0125	AIA-(BB_0794,BB_0794/BB_0795,BB_0795)	2242.036294	-1.51380039	0.260264023	-5.816402779	6.01E-09	1.42E-07
ncRNA0132	pI-(BB_0845a,BB_0845a/BB_0845b)	211.2546476	-1.882309932	0.534541277	-3.521355622	0.000429346	0.002649192
ncRNA0133	A-(BB_B03)	471.6283693	-1.017684262	0.245633747	-4.143096275	3.43E-05	0.000299246
ncRNA0136	AI-(BB_B09,BB_B09/BB_B10)	476.369844	-1.314926159	0.352213017	-3.733326407	0.000188967	0.001302881
ncRNA0144	AIA-(BB_P01,BB_P01/BB_P02,BB_P02)	5.386579535	-2.405247545	0.855087975	-2.812865595	0.004910217	0.020149638
ncRNA0148	A-(BB_P21)	93.09625772	-1.057974939	0.420039394	-2.518751701	0.011777167	0.040193419
ncRNA0152	IA-(BB_P32/BB_P33,BB_P33)	276.3515173	2.167303442	0.364938587	5.938816884	2.87E-09	7.64E-08
ncRNA0153	AIA-(BB_P35,BB_P35/BB_P36,BB_P36)	34.01766031	-1.98207391	0.798737356	-2.481508965	0.013082742	0.043686097
ncRNA0168	A-(BB_R43)	73.05907492	-1.339319655	0.46258358	-2.89530306	0.003787926	0.016127096
ncRNA0185	I-(BB_O29/BB_O30)	1573.01516	1.543988845	0.477729846	3.231928796	0.001229577	0.006403576
ncRNA0186	AIA-(BB_O32,BB_O32/BB_O33,BB_O33)	90.75800452	1.563150832	0.325703169	4.799311092	1.59E-06	2.06E-05
ncRNA0187	A-(BB_O36)	30.42655709	-1.803465374	0.720330268	-2.503664573	0.01229145	0.041402801
ncRNA0191	A-(BB_O44)	8.708649435	-2.530781759	0.797617394	-3.172926993	0.001509105	0.007514635
ncRNA0200	IA-(BB_L29/BB_L30,BB_L30)	867.0447963	-1.763677237	0.352133584	-5.008545957	5.48E-07	8.12E-06
ncRNA0218	IA-(BB_N32/BB_N33,BB_N33)	29.8614237	2.060586205	0.516857045	3.986762348	6.70E-05	0.000525662
ncRNA0226	P-(BB_D05a)	56.8286166	-5.381016157	0.67963523	-7.917506215	2.42E-15	1.29E-13
ncRNA0229	I-(BB_D18/BB_D20)	117.8530042	-1.450639654	0.345422551	-4.199609001	2.67E-05	0.000248821
ncRNA0231	p-(BB_D20)	3348.603578	-1.31009764	0.387511439	-3.380797334	0.000722758	0.004089227
ncRNA0232	I-(BB_D22/BB_D23)	141.5006657	-1.092538093	0.252136531	-4.333120985	1.47E-05	0.000146408
ncRNA0233	p-(BB_D23)	46.44975772	-2.615412768	0.750703561	-3.483948797	0.000494074	0.002973173
ncRNA0239	A-(BB_E09)	1464.223554	-2.363199357	0.428192961	-5.519005611	3.41E-08	6.91E-07
ncRNA0240	A-(BB_E09)	74.83749	-2.991500413	0.853438126	-3.50523409	0.000456206	0.002764835
ncRNA0242	I-(BB_E23b/BB_E29a)	107.0500482	-1.832009291	0.592483694	-3.092083897	0.001987567	0.009534721
ncRNA0245	I-(BB_E31/BB_E33)	314.7614859	-1.875521284	0.485353166	-3.864240341	0.000111435	0.000825107
ncRNA0246	I-(BB_E31/BB_E33)	124.5161323	-1.367831258	0.432304443	-3.164046267	0.001555921	0.007725168
ncRNA0247	A-(BB_F03)	1699.002338	-3.043049689	0.293976557	-10.35133453	4.13E-25	5.41E-23
ncRNA0248	AIP-(BB_F03,BB_F03/BB_F05,BB_F05)	239.2275787	-1.691341153	0.556097881	-3.041445059	0.002354455	0.010985308
ncRNA0249	pI-(BB_F05,BB_F05/BB_F06)	9.67900222	-2.374420185	0.741158663	-3.203659762	0.001356927	0.006931911
ncRNA0250	PI-(BB_F11a,BB_F11a/BB_F12)	15.60876515	-2.95590859	0.733796314	-4.028241261	5.62E-05	0.000453887
ncRNA0251	I-(BB_F11a/BB_F12)	727.7453318	-3.628950353	0.253865442	-14.29477888	2.36E-46	1.00E-43
ncRNA0252	Ip-(BB_F14/BB_F14a,BB_F14a)	58.86305107	-1.389686207	0.459183251	-3.026430526	0.002474597	0.011514312
ncRNA0253	pIp-(BB_F14a,BB_F14a/BB_F16,BB_F16)	16.1459582	-1.986715918	0.793616815	-2.503369235	0.012301713	0.041402801
ncRNA0255	I-(BB_F0040/BB_F32)	49.42843423	-2.200046006	0.637668726	-3.450139414	0.000560297	0.00327899
ncRNA0257	PI-(BB_G05,BB_G05/BB_G06)	56.23393737	-3.085696694	0.788709911	-3.912334118	9.14E-05	0.000694948
ncRNA0259	AA-(BB_G07,BB_G08)	88.149747	1.50709705	0.260690153	5.781181338	7.42E-09	1.71E-07
ncRNA0263	IA-(BB_G28/BB_G29,BB_G29)	24.0789363	-1.668736711	0.650242423	-2.566330111	0.010278096	0.036239332
ncRNA0271	P-(BB_H30)	22.38915613	-1.767197173	0.655615851	-2.695476582	0.007028802	0.026365748
ncRNA0281	IpI-(BB_K09/BB_K10,BB_K10,BB_K10/BB_K12)	166.2483876	-1.672741729	0.505577971	-3.308573206	0.000937727	0.005053634
ncRNA0284	A-(BB_K17)	123.8271122	-2.273935919	0.296721246	-7.663542628	1.81E-14	8.80E-13
ncRNA0285	A-(BB_K19)	59.46915813	-1.710662288	0.514990052	-3.321738508	0.000894585	0.004882943
ncRNA0286	A-(BB_K19)	219.9417934	-1.500183594	0.498015038	-3.012325895	0.002592541	0.011876819
ncRNA0287	I-(BB_K55/BB_K56)	86.5607051	-2.023857127	0.572688859	-3.53395582	0.00040939	0.0025632
ncRNA0289	AIA-(BB_K33,BB_K33/BB_K34,BB_K34)	54.0182385	-1.710605599	0.416989275	-4.10227721	4.09E-05	0.000348352
ncRNA0297	A-(BB_J18)	206.5236032	-2.307138732	0.688145025	-3.352692597	0.000800295	0.004439424
ncRNA0299	I-(BB_J20/BB_J0058)	119.1266624	-1.713102967	0.322707732	-5.308527805	1.11E-07	1.90E-06
ncRNA0300	I-(BB_J20/BB_J0058)	169.4427612	-1.622166436	0.5479595	-2.960376517	0.003072633	0.013686553
ncRNA0304	I-(BB_J37/BB_J41)	103.2796554	-1.321009225	0.482436974	-2.738200627	0.006177638	0.023910266
ncRNA0306	I-(BB_J37/BB_J41)	3515.789368	1.039600627	0.355673147	2.922910082	0.003467766	0.014950902
ncRNA0307	IA-(BB_J37/BB_J41,BB_J41)	1187.105717	1.028436646	0.375322809	2.740138948	0.006141322	0.023823851
ncRNA0308	I-(BB_J50/BB_J51)	893.058206	-5.421448561	0.494839696	-10.95596938	6.22E-28	1.06E-25
ncRNA0310	Ip-(BB_J50/BB_J51,BB_J51)	106.491945	-1.600677163	0.607456387	-2.635048699	0.008412521	0.030809728
ncRNA0311	A-(BB_A04)	361.981407	1.92915261	0.411397747	4.689263914	2.74E-06	3.36E-05
ncRNA0318	IP-(BB_A16/BB_A18,BB_A18)	1800.37672	-2.047895874	0.566676993	-3.613868038	0.000301663	0.001983519
ncRNA0322	I-(BB_A37/BB_A38)	2507.453992	-1.934721258	0.464555498	-4.164671965	3.12E-05	0.000276561
ncRNA0325	A-(BB_A66)	457.9294428	-1.540030266	0.339220102	-4.539914515	5.63E-06	6.35E-05
ncRNA0326	I-(BB_A66/BB_A68)	456.2996811	1.397904593	0.367271858	3.806184882	0.000141127	0.001014089
ncRNA0327	I-(BB_A68/BB_A69)	288.5694033	1.134754485	0.411545698	2.75729886	0.005828106	0.022764367
ncRNA0328	I-(BB_A73/BB_A74)	55.68358442	-1.579146243	0.646463656	-2.44274559	0.014576008	0.047920738
ncRNA0344	A-(BB_Q52)	10.93288614	-2.492065144	0.858939246	-2.901328767	0.003715838	0.01585983
ncRNA0353	IA-(BB_Q85/BB_Q88,BB_Q88)	66.89124586	-1.49929536	0.616918906	-2.430295692	0.015086509	0.049219013
lcl|NC_001318.1_cds_NP_212138.2_3	BB_0004	3213.612708	-1.011843497	0.350191279	-2.889402332	0.003859749	0.016310552
lcl|NC_001318.1_cds_NP_212156.1_20	ruvB	2074.85845	-1.076565311	0.202076814	-5.327505365	9.96E-08	1.75E-06
lcl|NC_001318.1_cds_NP_212161.1_25	BB_0027	9710.085949	-1.78806112	0.33743615	-5.298961365	1.16E-07	1.96E-06
lcl|NC_001318.1_cds_NP_212169.1_33	BB_0035	2717.907721	1.110406753	0.214694041	5.172042722	2.32E-07	3.65E-06
lcl|NC_001318.1_cds_NP_212306.2_161	BB_0172	2887.764892	-1.217319862	0.123742701	-9.837508402	7.76E-23	8.81E-21
lcl|NC_001318.1_cds_NP_212319.1_174	BB_0185	1345.323777	1.349770078	0.218738922	6.17069	6.80E-10	1.93E-08
lcl|NC_001318.1_cds_NP_212419.2_270	BB_0285	4139.079468	-1.154948662	0.317610837	-3.636364149	0.000276513	0.001839462
lcl|NC_001318.1_cds_NP_212464.1_313	BB_0330	9233.475103	1.149087729	0.219007371	5.246799329	1.55E-07	2.56E-06
lcl|NC_001318.1_cds_NP_212468.1_317	BB_0334	3335.870455	1.049316796	0.157717762	6.653130124	2.87E-11	1.14E-09
lcl|NC_001318.1_cds_NP_212498.1_345	mgsA	3019.346339	1.742561091	0.164753604	10.57677071	3.82E-26	5.42E-24
lcl|NC_001318.1_cds_NP_212499.1_346	la7	8401.745644	1.813485579	0.28233191	6.423239871	1.33E-10	4.09E-09
lcl|NC_001318.1_cds_NP_212541.1_388	manA	2887.103188	1.179754844	0.149300098	7.901902686	2.75E-15	1.42E-13
lcl|NC_001318.1_cds_NP_212542.1_389	fruA1	6219.62582	1.501253133	0.222065333	6.760411964	1.38E-11	5.86E-10
lcl|NC_001318.1_cds_NP_212549.1_394	BB_0415	1448.604328	-1.231446613	0.221932825	-5.548735809	2.88E-08	5.98E-07
lcl|NC_001318.1_cds_NP_212568.1_409	BB_0434	410.6547229	-1.026236311	0.305759261	-3.356353976	0.000789774	0.004409788
lcl|NC_001318.1_cds_NP_212585.1_426	BB_0451	435.9912764	1.065285948	0.208990807	5.097286147	3.45E-07	5.33E-06
lcl|NC_001318.1_cds_NP_212620.2_459	rpmC	492.1829109	1.007744941	0.202337379	4.980517913	6.34E-07	9.31E-06
lcl|NC_001318.1_cds_NP_212643.1_482	BB_0509	8232.921912	-2.925501491	0.365731686	-7.999037549	1.25E-15	6.89E-14
lcl|NC_001318.1_cds_NP_212652.1_488	dnaK	5264.983523	1.178215406	0.19904193	5.919433176	3.23E-09	8.21E-08
lcl|NC_001318.1_cds_NP_212671.1_500	BB_0537	1848.252264	1.232384605	0.238276054	5.172087516	2.31E-07	3.65E-06
lcl|NC_001318.1_cds_NP_212672.1_501	BB_0538	967.7451027	1.121459917	0.239070239	4.690922314	2.72E-06	3.36E-05
lcl|NC_001318.1_cds_NP_212696.1_524	BB_0562	1222.608908	1.195586657	0.147028901	8.13164381	4.24E-16	2.49E-14
lcl|NC_001318.1_cds_NP_212711.1_539	BB_0577	1867.115855	-1.438036835	0.222937044	-6.450416729	1.12E-10	3.59E-09
lcl|NC_001318.1_cds_NP_212722.1_550	BB_0588	1645.236707	-1.769614933	0.130872183	-13.52170412	1.16E-41	3.31E-39
lcl|NC_001318.1_cds_NP_212751.1_578	BB_0617	777.7214187	1.015625323	0.226394111	4.486094267	7.25E-06	8.07E-05
lcl|NC_001318.1_cds_NP_212760.1_587	rnmV	17091.35904	-1.237327813	0.410056749	-3.017455062	0.002549068	0.011824118
lcl|NC_001318.1_cds_NP_212771.2_597	BB_0637	8048.197727	1.095157563	0.261344496	4.190474945	2.78E-05	0.00025591
lcl|NC_001318.1_cds_NP_212772.1_598	BB_0638	3886.718713	1.105574398	0.209984884	5.265018978	1.40E-07	2.34E-06
lcl|NC_001318.1_cds_NP_212773.1_599	potD	1482.689456	1.254211323	0.286927391	4.371180168	1.24E-05	0.000125269
lcl|NC_001318.1_cds_NP_212778.1_604	nanE	697.3884376	1.073611768	0.19917336	5.39033818	7.03E-08	1.32E-06
lcl|NC_001318.1_cds_NP_212812.1_638	BB_0678	5061.700522	1.19084516	0.184625608	6.450054089	1.12E-10	3.59E-09
lcl|NC_001318.1_cds_NP_212813.2_639	BB_0679	4281.290839	1.087255066	0.192459876	5.649255778	1.61E-08	3.47E-07
lcl|NC_001318.1_cds_NP_212828.2_653	ffh	14060.76269	-2.511684041	0.374188652	-6.71234691	1.92E-11	7.77E-10
lcl|NC_001318.1_cds_YP_008686588.1_680	cabP	3690.875669	-1.146485517	0.267498556	-4.285950297	1.82E-05	0.000176067
lcl|NC_001318.1_cds_NP_212900.1_719	cvpA	420.5109223	1.157542839	0.239778975	4.827541022	1.38E-06	1.86E-05
lcl|NC_001318.1_cds_NP_212901.1_720	murG	901.267597	1.366187686	0.281129643	4.859635828	1.18E-06	1.62E-05
lcl|NC_001318.1_cds_NP_212902.1_721	BB_0768	1156.42566	1.01241342	0.234590479	4.315662868	1.59E-05	0.000156641
lcl|NC_001318.1_cds_NP_212903.1_722	BB_0769	1653.77636	1.253523799	0.240916322	5.203150152	1.96E-07	3.18E-06
lcl|NC_001318.1_cds_NP_212904.1_723	BB_0770	1364.054451	1.318173253	0.24452395	5.390773603	7.02E-08	1.32E-06
lcl|NC_001318.1_cds_NP_212907.1_727	BB_0773	467.79866	1.273917781	0.178834561	7.123442872	1.05E-12	4.84E-11
lcl|NC_001318.1_cds_NP_212919.1_739	spoVG	2664.703766	1.261401239	0.211981895	5.950514032	2.67E-09	7.23E-08
lcl|NC_001318.1_cds_YP_008686594.1_748	BB_0794	8844.212411	-1.020839036	0.17423885	-5.858848551	4.66E-09	1.15E-07
lcl|NC_001318.1_cds_NP_212932.1_752	BB_0798	412.7429471	-1.367264622	0.230607112	-5.928978559	3.05E-09	7.94E-08
lcl|NC_001318.1_cds_NP_212975.1_793	arcA	2690.756525	1.011615231	0.157525181	6.421927127	1.35E-10	4.09E-09
lcl|NC_001318.1_cds_NP_212976.2_794	arcB	2452.381364	1.161259482	0.247934672	4.6837317	2.82E-06	3.43E-05
lcl|NC_001903.1_cds_NP_046987.2_798	BB_B01	877.5381411	1.388320735	0.215561895	6.440473802	1.19E-10	3.76E-09
lcl|NC_001903.1_cds_NP_046988.1_799	BB_B02	2128.099072	1.093811634	0.196510568	5.566172052	2.60E-08	5.47E-07
lcl|NC_001903.1_cds_NP_046990.2_801	chbC	11469.16755	1.518292428	0.224234778	6.77099442	1.28E-11	5.58E-10
lcl|NC_001903.1_cds_NP_046991.1_802	chbA	2551.066468	1.802467823	0.276127156	6.527673154	6.68E-11	2.42E-09
lcl|NC_001903.1_cds_NP_046992.2_803	chbB	1687.198719	2.399229955	0.263942445	9.089973967	9.91E-20	8.88E-18
lcl|NC_001903.1_cds_NP_046993.1_804	BB_B07	8409.809781	1.379859483	0.163157096	8.457244702	2.74E-17	1.79E-15
lcl|NC_001903.1_cds_NP_047004.2_812	guaA	11235.00282	1.122969516	0.136736824	8.212634214	2.16E-16	1.32E-14
lcl|NC_001903.1_cds_NP_047005.1_813	ospC	292.2425211	-1.444790637	0.487067621	-2.966304013	0.003014024	0.013507586
lcl|NC_001903.1_cds_NP_047009.2_815	BB_B23	2583.013976	1.194275661	0.153920788	7.759027729	8.56E-15	4.29E-13
lcl|NC_001903.1_cds_NP_047013.1_819	BB_B27	1716.86092	1.09085916	0.184033083	5.927516626	3.08E-09	7.94E-08
lcl|NC_001903.1_cds_NP_047014.1_820	BB_B28	4249.142152	1.266728604	0.143683797	8.816085248	1.19E-18	9.18E-17
lcl|NC_001903.1_cds_NP_047015.1_821	BB_B29	24118.646	1.407250063	0.212691935	6.616377174	3.68E-11	1.42E-09
lcl|NC_000948.1_cds_NP_051171.1_830	BB_P10	89.29313498	-1.665579649	0.568149994	-2.931584381	0.003372377	0.014650915
lcl|NC_000948.1_cds_NP_051190.1_849	BB_P29	117.5902665	-1.925349814	0.651102731	-2.95705996	0.003105877	0.013738464
lcl|NC_000948.1_cds_NP_051192.1_851	BB_P31	319.4277675	1.450607612	0.331481902	4.37612915	1.21E-05	0.000123284
lcl|NC_000948.1_cds_NP_051193.1_852	BB_P32	812.8429087	1.909156521	0.235635362	8.102164722	5.40E-16	3.06E-14
lcl|NC_000948.1_cds_NP_051194.2_853	BB_P33	507.5530008	2.135405263	0.245441907	8.700247189	3.31E-18	2.45E-16
lcl|NC_000948.1_cds_NP_051195.1_854	bdrA	840.2082301	1.327617956	0.302692249	4.386032214	1.15E-05	0.000119145
lcl|NC_000948.1_cds_NP_051196.1_855	bppA	59.0359037	-1.406828985	0.558482981	-2.519018542	0.011768246	0.040193419
lcl|NC_000948.1_cds_NP_051197.1_856	bppB	16.41223164	-1.500572432	0.595533131	-2.519712763	0.011745063	0.040193419
lcl|NC_000949.1_cds_NP_051234.2_890	BB_S31	105.7674492	-1.61576679	0.504778499	-3.200942183	0.00136979	0.006963441
lcl|NC_000949.1_cds_NP_051237.1_892	BB_S34	208.7781932	1.122702036	0.372157025	3.016742832	0.002555065	0.011824118
lcl|NC_000949.1_cds_NP_051238.1_893	BB_S35	265.7310316	1.325298003	0.239761792	5.52756131	3.25E-08	6.66E-07
lcl|NC_000949.1_cds_NP_051240.1_894	bdrE	326.736536	1.358376963	0.242081995	5.611226741	2.01E-08	4.28E-07
lcl|NC_000949.1_cds_NP_051241.2_895	bppA	41.41867394	-2.507569395	0.644486353	-3.890802937	9.99E-05	0.000752885
lcl|NC_000950.1_cds_NP_051274.2_927	bdrH	752.05999	-1.019325728	0.238699916	-4.270322938	1.95E-05	0.000186747
lcl|NC_000950.1_cds_NP_051278.1_930	BB_R31	1579.32556	-1.113009695	0.443119222	-2.51176126	0.012013033	0.040672355
lcl|NC_000950.1_cds_NP_051280.1_932	BB_R33	295.7891493	1.070632049	0.254705484	4.203411853	2.63E-05	0.000246019
lcl|NC_000950.1_cds_NP_051281.1_933	BB_R34	206.0163455	1.161125002	0.218429448	5.315789667	1.06E-07	1.85E-06
lcl|NC_000950.1_cds_NP_051282.1_934	bppA	20.6580321	-2.419159274	0.672161892	-3.599072338	0.000319354	0.002075803
lcl|NC_000951.1_cds_NP_051301.1_951	BB_M10	26.57919292	-1.783800995	0.552979602	-3.225798903	0.001256216	0.00650254
lcl|NC_000951.1_cds_NP_051329.2_978	erpK	89.22936299	-1.238045803	0.471227478	-2.627278462	0.008607087	0.031253451
lcl|NC_000951.1_cds_NP_051330.1_979	BB_M39	174.8340308	-1.364514032	0.518402401	-2.632152222	0.008484584	0.031006966
lcl|NC_000952.1_cds_NP_051338.1_986	BB_O05	56.56386104	-1.292207756	0.465504454	-2.775929952	0.005504407	0.021850826
lcl|NC_000952.1_cds_NP_051362.1_1010	BB_O29	207.6325904	-1.106695965	0.441931172	-2.50422698	0.012271928	0.041402801
lcl|NC_000952.1_cds_NP_051365.1_1013	BB_O32	381.8142121	1.026021902	0.245024295	4.187429255	2.82E-05	0.00025591
lcl|NC_000952.1_cds_NP_051366.1_1014	BB_O33	262.1173124	1.010347941	0.210338316	4.803442187	1.56E-06	2.04E-05
lcl|NC_000952.1_cds_NP_051372.1_1020	erpL	12.95799511	-1.949705703	0.71377829	-2.731528444	0.00630413	0.02418003
lcl|NC_000953.1_cds_NP_051387.1_1034	BB_L10	89.29313498	-1.665579649	0.568149994	-2.931584381	0.003372377	0.014650915
lcl|NC_000954.1_cds_NP_051443.1_1087	BB_N31	161.0855567	1.627410115	0.348823424	4.665426693	3.08E-06	3.69E-05
lcl|NC_000954.1_cds_NP_051444.1_1088	BB_N32	241.9969265	1.586427412	0.308950619	5.134889897	2.82E-07	4.41E-06
lcl|NC_000954.1_cds_NP_051445.1_1089	BB_N33	157.3216497	1.888712116	0.288264846	6.552002924	5.68E-11	2.10E-09
lcl|NC_000954.1_cds_NP_051446.1_1090	bdrQ	227.796319	1.701845715	0.263258846	6.464533837	1.02E-10	3.46E-09
lcl|NC_000954.1_cds_NP_051450.1_1094	erpQ	1968.305688	1.458291354	0.359888349	4.052066028	5.08E-05	0.000415658
lcl|NC_001849.2_cds_NP_045388.1_1111	BB_D04	31.08243243	-1.094553278	0.428260926	-2.555809345	0.010594115	0.037122999
lcl|NC_001849.2_cds_NP_045397.1_1115	BB_D13	908.2099786	-1.394902701	0.304581916	-4.57972922	4.66E-06	5.36E-05
lcl|NC_001849.2_cds_NP_045398.1_1116	BB_D14	3487.083186	-1.744927691	0.326860939	-5.338440546	9.37E-08	1.66E-06
lcl|NC_001849.2_cds_NP_045404.1_1119	BB_D21	967.6859329	-1.895897547	0.440022711	-4.308635668	1.64E-05	0.000159853
lcl|NC_001849.2_cds_NP_045405.1_1120	BB_D22	128.5625749	-1.23419393	0.316273386	-3.902300932	9.53E-05	0.000721183
lcl|NC_001849.2_cds_YP_004940417.1_1121	BB_D0031	25.13306541	-2.773679485	0.70351362	-3.942609505	8.06E-05	0.000626765
lcl|NC_001850.1_cds_NP_045416.1_1133	BB_E09	524.595921	-1.11887635	0.396427462	-2.82239869	0.004766588	0.019798779
lcl|NC_001850.1_cds_NP_045428.1_1139	BB_E21	8358.69302	-1.131514218	0.253371318	-4.465833882	7.98E-06	8.76E-05
lcl|NC_001850.1_cds_NP_045436.1_1141	BB_E31	93.10926862	-1.879099504	0.381704541	-4.922916288	8.53E-07	1.21E-05
lcl|NC_001851.2_cds_YP_004940409.1_1142	arp	9.140034358	-1.998405324	0.818669073	-2.441041673	0.014644965	0.047971938
lcl|NC_001851.2_cds_NP_045439.1_1144	repU	189.0840177	-2.286930833	0.378577934	-6.040845561	1.53E-09	4.21E-08
lcl|NC_001851.2_cds_NP_045442.1_1145	BB_F06	11.18133266	-2.261136123	0.827384713	-2.732871528	0.006278482	0.024136014
lcl|NC_001851.2_cds_NP_045444.1_1146	BB_F08	152.5453178	-1.253124952	0.415636758	-3.014952189	0.002570198	0.011848455
lcl|NC_001851.2_cds_YP_004940410.1_1147	BB_F0034	131.6802735	-1.220309664	0.498446076	-2.448228046	0.014356076	0.047564976
lcl|NC_001851.2_cds_NP_045449.2_1148	BB_F14	47.65068541	-3.144088867	0.736154608	-4.270962693	1.95E-05	0.000186747
lcl|NC_001851.2_cds_YP_004940411.1_1149	BB_F17	21.28080946	-2.380548185	0.759226444	-3.13549166	0.001715663	0.008444434
lcl|NC_001851.2_cds_NP_045453.2_1150	BB_F20	136.227553	-2.849065131	0.295141639	-9.653213099	4.76E-22	4.77E-20
lcl|NC_001851.2_cds_NP_045457.1_1152	BB_F24	283.2747783	-2.12742045	0.368574807	-5.772018074	7.83E-09	1.78E-07
lcl|NC_001851.2_cds_NP_045458.1_1153	BB_F25	156.0679977	-1.989776392	0.451280505	-4.409178704	1.04E-05	0.000109757
lcl|NC_001851.2_cds_NP_045459.1_1154	BB_F26	1080.037774	-2.756403353	0.444938874	-6.195015798	5.83E-10	1.68E-08
lcl|NC_001851.2_cds_YP_004940414.1_1157	BB_F0041	431.5944656	-2.393300878	0.516787972	-4.63110794	3.64E-06	4.27E-05
lcl|NC_001852.1_cds_NP_045464.1_1159	BB_G02	182.6516298	1.367896406	0.397186284	3.443966874	0.000573246	0.00334328
lcl|NC_001852.1_cds_NP_045472.1_1163	BB_G12	81.95387397	-2.359314711	0.670799011	-3.517170827	0.000436173	0.002671952
lcl|NC_001852.1_cds_NP_045481.1_1172	BB_G21	257.5363142	-1.920188356	0.710774817	-2.701542473	0.006901866	0.02611973
lcl|NC_001852.1_cds_NP_045482.1_1174	BB_G22	64.42227065	-2.664763491	0.638466233	-4.17369526	3.00E-05	0.000268625
lcl|NC_001853.1_cds_NP_045498.1_1188	BB_H04	146.0244758	-1.764826058	0.517657817	-3.40925221	0.000651412	0.003760526
lcl|NC_001853.1_cds_NP_045510.1_1195	BB_H17	10.29555978	-2.234942039	0.800322419	-2.792552085	0.005229405	0.02096611
lcl|NC_001853.1_cds_NP_045516.1_1196	BB_H25	30.47678431	-1.561052859	0.518258182	-3.012114256	0.002594349	0.011876819
lcl|NC_001853.1_cds_NP_045517.1_1197	BB_H26	1195.446691	-1.591603607	0.4611763	-3.451182569	0.000558136	0.003277605
lcl|NC_001855.1_cds_NP_045596.1_1233	BB_K22	260.1823499	-1.082818368	0.34636474	-3.126237301	0.001770586	0.008639852
lcl|NC_001855.1_cds_NP_045597.1_1234	BB_K23	2453.836139	-1.76010754	0.38683042	-4.550075304	5.36E-06	6.09E-05
lcl|NC_001855.1_cds_NP_045598.1_1235	BB_K24	377.5769784	-1.774945995	0.437272966	-4.05912584	4.93E-05	0.000405238
lcl|NC_001855.1_cds_YP_004940636.1_1236	BB_K54	131.6013456	-1.957517875	0.652698517	-2.999114942	0.002707651	0.01236228
lcl|NC_001855.1_cds_NP_045605.1_1237	BB_K32	213.6510062	-1.873429774	0.489655712	-3.826014332	0.000130235	0.000943786
lcl|NC_001855.1_cds_NP_045606.1_1238	BB_K33	37.62541137	-1.379270088	0.444039976	-3.106184497	0.001895184	0.009169031
lcl|NC_001855.1_cds_NP_045607.1_1239	BB_K34	221.9972259	-1.173040964	0.21313803	-5.503668033	3.72E-08	7.45E-07
lcl|NC_001855.1_cds_YP_004940637.1_1241	BB_K0058	51.97230439	-1.399070285	0.544398454	-2.569938021	0.010171671	0.035977936
lcl|NC_001855.1_cds_NP_045612.1_1242	BB_K40	3811.74482	-1.359892512	0.231723983	-5.868587679	4.40E-09	1.10E-07
lcl|NC_001855.1_cds_NP_045618.1_1246	BB_K47	2505.671645	1.249316306	0.317102255	3.939790037	8.16E-05	0.000631294
lcl|NC_001855.1_cds_NP_045620.1_1248	BB_K49	1392.93963	1.08561374	0.354606502	3.061460336	0.002202602	0.010333418
lcl|NC_001855.1_cds_NP_045623.1_1251	BB_K52	62.12418253	-1.650671092	0.586187285	-2.815944895	0.004863401	0.020054168
lcl|NC_001855.1_cds_NP_045624.1_1252	BB_K53	359.8395733	-1.319843975	0.48424027	-2.725597306	0.006418525	0.024508405
lcl|NC_001856.1_cds_NP_045633.1_1254	BB_J09	24951.96368	1.809636522	0.37729147	4.796388644	1.62E-06	2.07E-05
lcl|NC_001856.1_cds_NP_045643.1_1260	BB_J19	8721.994493	-1.226737445	0.357426229	-3.43214164	0.000598835	0.00346876
lcl|NC_001856.1_cds_NP_045647.2_1263	BB_J23	37.83775028	-2.452974412	0.487590759	-5.030805791	4.88E-07	7.31E-06
lcl|NC_001856.1_cds_NP_045648.1_1264	BB_J24	96.33456458	-1.30740669	0.376420695	-3.473259329	0.000514178	0.003045537
lcl|NC_001856.1_cds_NP_045650.1_1266	BB_J26	38.55968829	-1.054612052	0.379750298	-2.777119751	0.005484297	0.021850826
lcl|NC_001856.1_cds_NP_045667.1_1276	BB_J43	43.39693922	-1.947979145	0.670370858	-2.905823132	0.003662884	0.015712574
lcl|NC_001857.2_cds_NP_045676.1_1287	BB_A03	12443.29488	2.136543909	0.336492459	6.349455542	2.16E-10	6.46E-09
lcl|NC_001857.2_cds_NP_045698.1_1304	dbpB	89.46809468	-2.646999244	0.558325827	-4.740957908	2.13E-06	2.70E-05
lcl|NC_001857.2_cds_NP_045703.1_1305	BB_A30	869.6149934	1.35254358	0.216997662	6.23298689	4.58E-10	1.34E-08
lcl|NC_001857.2_cds_NP_045704.1_1306	BB_A31	693.6564221	1.565907435	0.26914935	5.817987062	5.96E-09	1.42E-07
lcl|NC_001857.2_cds_NP_045707.1_1309	BB_A34	190.0838372	-2.164189119	0.577886296	-3.745008546	0.000180388	0.001253343
lcl|NC_001857.2_cds_NP_045709.2_1310	BB_A36	46.52933338	-2.130484898	0.582127955	-3.659822345	0.00025239	0.00168557
lcl|NC_001857.2_cds_NP_045710.1_1311	BB_A37	93.94411642	-1.91040809	0.652530567	-2.927691339	0.003414889	0.01477655
lcl|NC_001857.2_cds_NP_045725.1_1323	BB_A52	1613.061442	1.01788004	0.312733352	3.254785695	0.00113478	0.005964598
lcl|NC_001857.2_cds_NP_045726.1_1324	BB_A53	375.6912082	1.346830886	0.375697753	3.584878732	0.000337235	0.002183692
lcl|NC_001857.2_cds_NP_045727.1_1325	BB_A54	599.9624458	1.900543585	0.367018587	5.178330612	2.24E-07	3.60E-06
lcl|NC_001857.2_cds_NP_045731.1_1327	BB_A58	8350.064089	1.298241485	0.296675316	4.375967316	1.21E-05	0.000123284
lcl|NC_001857.2_cds_NP_045733.1_1329	BB_A60	1031.73146	1.00641711	0.222117345	4.531015402	5.87E-06	6.58E-05
lcl|NC_001857.2_cds_NP_045738.1_1333	BB_A65	185.7509576	-1.571198149	0.496762792	-3.162874059	0.001562199	0.007733793
lcl|NC_001857.2_cds_YP_004940408.1_1337	BB_A0078	99.0563379	-1.7817888	0.581545432	-3.063885815	0.002184823	0.010300298
lcl|NC_001857.2_cds_NP_045746.1_1338	BB_A73	233.2233394	-1.829096851	0.424452475	-4.309308956	1.64E-05	0.000159853
lcl|NC_001857.2_cds_NP_045747.1_1339	osm28	12273.60539	2.234165789	0.394208008	5.667479461	1.45E-08	3.16E-07
lcl|NC_000956.1_cds_NP_051489.1_1361	BB_Q27	184.692011	1.478250023	0.447598087	3.302628105	0.000957833	0.005142466
lcl|NC_000956.1_cds_NP_051502.1_1373	BB_Q40	481.1703282	1.463624848	0.367880474	3.978533659	6.93E-05	0.000541691
lcl|NC_000956.1_cds_NP_051504.1_1374	bdrV	634.196218	2.699534338	0.317216535	8.510068181	1.74E-17	1.18E-15
lcl|NC_000956.1_cds_NP_051521.1_1385	BB_Q62	20.48709362	2.091457406	0.544783424	3.839062122	0.000123505	0.000902701
lcl|NC_000956.1_cds_NP_051533.1_1388	BB_Q85	31.08243243	-1.094553278	0.428260926	-2.555809345	0.010594115	0.037122999

The known or proposed functions of the DE ORFs support the hypothesis that CsrA controls a diverse regulon. Deletion of *csrA* negatively affected transcripts for several outer surface proteins that are involved with transmission from ticks and with survival within the vertebrate host, including *ospC* (down 2.7-fold), *dbpB* (down 6.3-fold), *vlsE* (down 5.3-fold), and *arp* (down 4.0-fold) [54–63]. In the remainder of this section, we focus on examples of transcripts that were affected only by the *csrA* mutation, while subsequent sections present information on transcripts that were impacted by *csrA* and one or more other mutations (i.e. regulome overlaps).

**Fig 3:**
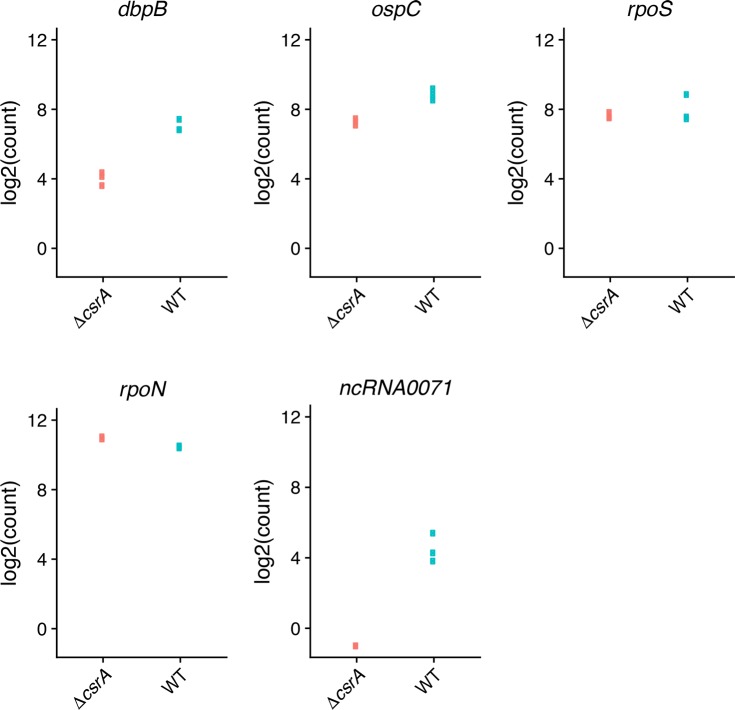
Log2 transformed counts of RNA-Seq results of select transcripts in *csrA* mutant and wild-type *B*. *burgdorferi*. Three replicates were assessed. Some values were essentially identical, and so appear to be single dots in the figures.

As noted above, previous studies of *csrA* mutant *B*. *burgdorferi* produced varied results, with some showing significant impacts on *rpoS* or RpoS-affected transcripts, while others did not observe such effects [24, 28]. Under the growth conditions employed in our studies, deletion of *csrA* did not significantly change levels of either the *rpoN* or *rpoS* mRNAs (Fig 3). However, the mutant did demonstrate significant changes in levels of several transcripts that were previously seen to be altered in some *rpoN* or *rpoS* mutants [5, 6]. For example, both *ospC* and *dbpB* were expressed at significantly lower levels in the *csrA* mutant than in the wild-type (Table 1). Those results suggest that at least some members of the previously-described RpoS regulon are also controlled through RpoS-independent mechanisms (see below).

Although deletion of *csrA* did not have any significant effects on either the *rpoN* or *rpoS* mRNAs, there was a significant, 25.7-fold decrease in the level of antisense RNA *ncRNA0071*, which is transcribed within the *rpoN* ORF (Fig 3) [30]. Please note that this report uses the ncRNA nomenclature of Arnold et al., 2016 [30], which was the first published description of the *B*. *burgdorferi* ncRNA transcriptome. Among other notable observations, numerous transcripts that encode outer-surface lipoproteins that play roles in vertebrate infection were present at lower amounts in the mutant. These included the fibronectin-binding protein-encoding *bbk32* and several members of the *erp* family [64–66].

Multiple transcripts encoding key nutrient scavenging proteins were present at higher levels in the mutant, including *guaA*, *BB_B23*, and *BB_B29*. GuaA and the *BB_B23*-encoded protein are involved in purine salvage and the uptake of hypoxanthine, respectively. GuaA is essential for the borrelial infectious cycle [67], and *BB_B23* mutants are defective in vertebrate infection [68]. ORF *BB_B29* encodes a putative glucose transporter and, while not absolutely essential for vertebrate infection, mutants are significantly defective for growth on certain carbohydrate sources [69]. Together, these data support the hypothesis that CsrA controls a range of systems that are important for survival in both the tick and vertebrate hosts.

### BadR controls transcripts of genes associated with catabolite uptake and utilization

Consistent with the previous array-based study of BadR [16], levels of a large number of transcripts were altered by deletion of *badR*. A number of these transcripts encode proteins involved in the uptake of catabolites from the extracellular milieu. Under the studied growth conditions, a total of 234 transcripts were DE in the *badR* mutant: 134 decreased and 100 increased (Fig 1B, Table 2 and S3 and S6 Tables). Similar to what was observed for the *csrA* mutant, approximately one third of DE transcripts were putative ncRNAs (69/234 or 29.5%). Of the 100 transcripts that increased in the *badR* mutant, only 14 (14%) were putative ncRNAs, whereas 55/134 (41.0%) of the reduced transcripts were putative ncRNAs. The ratio of affected transcripts was slightly skewed towards the plasmids, with 129 (55.1%) transcripts originating from the small native replicons. Transcripts in elevated abundance reflected a plasmid vs. chromosome bias consistent with the overall trend, with 65/100 (65%), whereas the bias was not maintained for the transcripts of reduced abundance 64/134 (47.8%). RNA-Seq results were validated by qRT-PCR analyses of select transcripts (Fig 2). A number of these transcripts were affected only in the *badR* mutant, while many were also altered in the *csrA* mutant. Examples of transcripts affected only by Δ*badR* are presented below, and those that overlapped with Δ*csrA* are presented in the subsequent section. We note, however, that deletion of *badR* did not affect levels of *csrA* transcript, indicating that regulon overlaps were due to convergence, rather than one protein operating through the other.

**Table 2:** Differentially expressed transcripts when comparing the badR mutant to wild-type, listed in order of genome reference number. The included transcripts met the criteria of >1 log2 fold-change and an adjusted p-value (padj) when comparing the *badR* mutant to wild-type. A total of 234 transcripts were differentially regulated, not including the mutated gene, by the mutation. The first column contains the CDS/custom transcript ID which is the transcript ID for all coding sequences obtained from the NCBI Gene file format file or the transcript ID given to ncRNAs. RefSeq entries are further separated by the character “_”. The first portion gives the genetic element from which it is derived, the second describes the type of element (CDS), the third provides RefSeq ID, and the fourth provides a number indicating the particular entries ordered number in the RefSeq entry. The second column is the gene information, for the ncRNAs it contains the location relative to other genes and for predicted or known genes it contains gene name. The remaining columns describe the various metrics of expression of each impacted transcript including, base mean (average library size normalized counts across all samples), log2FC (Fold change estimate), lfcSE (uncertainty of the log fold change estimate), stat (Wald statistic), pvalue, padj (pvalue following Benjamini-Hochberg adjustment). ORFs and ncRNAs are identified according to the names or numbers assigned to genes and transcripts by the initial genome sequencing of *B*. *burgdorferi* strain B31 [18, 31] or from our previous analyses of that strain’s ncRNA transcriptome [30].

RefSeq CDS/Custom Transcript ID	Gene Name	baseMean	log2FoldChange	lfcSE	stat	pvalue	padj
ncRNA0001	PI-(BB_0003,BB_0003/BB_0004)	968.8208685	-1.091121118	0.280770351	-3.886169302	0.000101838	0.00069651
ncRNA0002	AI-(BB_0004,BB_0004/BB_0005)	3041.858416	-1.466630978	0.248955372	-5.891140104	3.84E-09	6.87E-08
ncRNA0003	AA-(BB_0005,BB_0006)	1243.576562	-1.402633207	0.311544968	-4.5021854	6.73E-06	5.78E-05
ncRNA0006	A-(BB_0013)	3033.894978	-1.651261635	0.339717919	-4.860684529	1.17E-06	1.18E-05
ncRNA0007	A-(BB_0014)	214.8526993	-1.496435951	0.482793757	-3.099534591	0.001938249	0.00909322
ncRNA0014	A-(BB_0084)	380.5460997	-1.168956757	0.38865126	-3.007726663	0.002632098	0.01162608
ncRNA0031	AA-(BB_0198,BB_0199)	400.7134034	-1.254495741	0.373991555	-3.354342427	0.000795538	0.004384473
ncRNA0035	A-(BB_0208)	510.9105757	-1.367867121	0.292134086	-4.682326331	2.84E-06	2.64E-05
ncRNA0037	A-(BB_0211)	1238.76926	-1.499844735	0.314759246	-4.76505378	1.89E-06	1.81E-05
ncRNA0042	A-(BB_0240)	2021.458735	-1.868463425	0.349137347	-5.351657282	8.72E-08	1.10E-06
ncRNA0043	A-(BB_0244)	577.795707	-2.193831378	0.40473779	-5.420376925	5.95E-08	7.73E-07
ncRNA0050	AIA-(BB_0269,BB_0269/BB_0270,BB_0270)	1285.298201	-1.970983739	0.556213454	-3.543574368	0.000394742	0.002392333
ncRNA0057	A-(BB_0347)	34.51259723	-1.771846181	0.548621557	-3.22963281	0.001239493	0.006263669
ncRNA0061	A-(BB_0374)	321.0807142	-1.221514521	0.292759095	-4.172422107	3.01E-05	0.0002261
ncRNA0063	A-(BB_0381)	391.0552137	-1.499645362	0.433017783	-3.463241977	0.000533708	0.003123385
ncRNA0070	A-(BB_0446)	529.3172145	-2.031253639	0.313402561	-6.481292412	9.09E-11	2.12E-09
ncRNA0071	A-(BB_0450)	65.28090439	-4.497370774	0.703386199	-6.39388544	1.62E-10	3.67E-09
ncRNA0072	A-(BB_0454)	396.651084	2.955889742	0.659489457	4.48208794	7.39E-06	6.29E-05
ncRNA0073	I-(BB_t06/BB_0461)	2784.933991	1.60500746	0.229931003	6.980387322	2.94E-12	8.09E-11
ncRNA0076	AA-(BB_0465,BB_0466)	1466.200068	-1.173752479	0.258622838	-4.538471893	5.67E-06	5.00E-05
ncRNA0080	p-(BB_0522)	879.8130905	-1.911466532	0.236932979	-8.067541052	7.17E-16	2.81E-14
ncRNA0083	AI-(BB_0556,BB_0556/BB_0557)	349.181935	-1.071796374	0.268731567	-3.988353087	6.65E-05	0.000470152
ncRNA0084	A-(BB_0581)	87.52936081	-2.554026659	0.470127152	-5.43262955	5.55E-08	7.33E-07
ncRNA0087	A-(BB_0588)	1536.156421	-2.232166273	0.709217961	-3.147362865	0.001647504	0.007948155
ncRNA0099	A-(BB_0633)	150.8640122	-4.912156619	0.640176561	-7.673127875	1.68E-14	6.08E-13
ncRNA0105	A-(BB_0660)	99.2736016	-2.451368375	0.757036542	-3.23811103	0.00120324	0.006116768
ncRNA0110	A-(BB_0697)	1608.781804	-1.363019634	0.256463472	-5.314673554	1.07E-07	1.30E-06
ncRNA0117	A-(BB_0747)	270.2800642	-1.177533684	0.473249509	-2.488187864	0.012839589	0.043805776
ncRNA0125	AIA-(BB_0794,BB_0794/BB_0795,BB_0795)	2242.036294	-1.573260538	0.260394058	-6.041844998	1.52E-09	2.98E-08
ncRNA0132	pI-(BB_0845a,BB_0845a/BB_0845b)	211.2546476	-2.25854887	0.535976359	-4.213896437	2.51E-05	0.000192548
ncRNA0133	A-(BB_B03)	471.6283693	-1.626316186	0.246864331	-6.587894579	4.46E-11	1.10E-09
ncRNA0134	AI-(BB_B03,BB_B03/BB_B04)	130.5038159	2.104778754	0.376423997	5.591510561	2.25E-08	3.24E-07
ncRNA0135	I-(BB_B03/BB_B04)	1218.809536	3.522740679	0.296249536	11.89112641	1.32E-32	1.49E-30
ncRNA0136	AI-(BB_B09,BB_B09/BB_B10)	476.369844	-2.090544907	0.354339634	-5.899833676	3.64E-09	6.59E-08
ncRNA0148	A-(BB_P21)	93.09625772	-1.906327102	0.42831528	-4.450756704	8.56E-06	7.25E-05
ncRNA0150	I-(BB_P29/BB_P30)	412.4991435	1.151296559	0.448344914	2.567881385	0.010232216	0.036685188
ncRNA0152	IA-(BB_P32/BB_P33,BB_P33)	276.3515173	2.117378095	0.365073141	5.799873664	6.64E-09	1.13E-07
ncRNA0154	A-(BB_S11)	104.5257947	-1.477242221	0.477036608	-3.096706199	0.001956837	0.00913012
ncRNA0185	I-(BB_O29/BB_O30)	1573.01516	1.545721747	0.477736479	3.235511242	0.001214251	0.006154374
ncRNA0186	AIA-(BB_O32,BB_O32/BB_O33,BB_O33)	90.75800452	1.658067092	0.325706168	5.090683735	3.57E-07	3.92E-06
ncRNA0188	A-(BB_O36)	23.57876286	-1.934664257	0.731373306	-2.64524866	0.008163091	0.030756069
ncRNA0191	A-(BB_O44)	8.708649435	-1.952083338	0.79304848	-2.461493072	0.013836007	0.0464051
ncRNA0200	IA-(BB_L29/BB_L30,BB_L30)	867.0447963	-2.254648069	0.353335763	-6.381035557	1.76E-10	3.89E-09
ncRNA0205	A-(BB_L36)	27.97464525	-1.847277027	0.738413586	-2.501683421	0.01236044	0.042697421
ncRNA0218	IA-(BB_N32/BB_N33,BB_N33)	29.8614237	2.012668676	0.517685749	3.887819354	0.000101149	0.000695772
ncRNA0225	I-(BB_D04/BB_D05a)	86.57888532	-2.205735552	0.542257921	-4.067687105	4.75E-05	0.00034119
ncRNA0226	P-(BB_D05a)	56.8286166	-4.172467124	0.631313043	-6.60918885	3.86E-11	9.68E-10
ncRNA0229	I-(BB_D18/BB_D20)	117.8530042	-1.351834939	0.345411029	-3.913699406	9.09E-05	0.000631798
ncRNA0233	p-(BB_D23)	46.44975772	-2.519658462	0.750954393	-3.355274949	0.000792861	0.004383906
ncRNA0239	A-(BB_E09)	1464.223554	-2.309739158	0.428228105	-5.393712208	6.90E-08	8.84E-07
ncRNA0240	A-(BB_E09)	74.83749	-2.662732492	0.853298616	-3.120516594	0.001805341	0.008612034
ncRNA0242	I-(BB_E23b/BB_E29a)	107.0500482	-1.634381518	0.591753121	-2.761931385	0.005746055	0.022704251
ncRNA0245	I-(BB_E31/BB_E33)	314.7614859	-1.445505713	0.484755182	-2.98192937	0.002864381	0.012380813
ncRNA0257	PI-(BB_G05,BB_G05/BB_G06)	56.23393737	-3.935288751	0.795248019	-4.948504938	7.48E-07	8.01E-06
ncRNA0259	AA-(BB_G07,BB_G08)	88.149747	1.10442889	0.263139801	4.197118353	2.70E-05	0.000205525
ncRNA0281	IpI-(BB_K09/BB_K10,BB_K10,BB_K10/BB_K12)	166.2483876	-1.374538803	0.505265076	-2.720431054	0.006519687	0.025291632
ncRNA0284	A-(BB_K17)	123.8271122	-2.104304342	0.296306341	-7.101786398	1.23E-12	3.62E-11
ncRNA0286	A-(BB_K19)	219.9417934	-1.262032589	0.497593607	-2.536271711	0.011203977	0.039528405
ncRNA0297	A-(BB_J18)	206.5236032	-4.034115008	0.717156855	-5.625150172	1.85E-08	2.74E-07
ncRNA0299	I-(BB_J20/BB_J0058)	119.1266624	-1.620207512	0.322869489	-5.018149959	5.22E-07	5.62E-06
ncRNA0304	I-(BB_J37/BB_J41)	103.2796554	-1.378344172	0.48298017	-2.853831809	0.004319539	0.017514704
ncRNA0308	I-(BB_J50/BB_J51)	893.058206	-5.20269521	0.494508761	-10.52093637	6.92E-26	4.91E-24
ncRNA0311	A-(BB_A04)	361.981407	2.135911713	0.411274034	5.19340278	2.06E-07	2.38E-06
ncRNA0318	IP-(BB_A16/BB_A18,BB_A18)	1800.37672	-1.63311484	0.56659464	-2.882333724	0.003947414	0.016316617
ncRNA0322	I-(BB_A37/BB_A38)	2507.453992	-1.827061965	0.464565418	-3.932841091	8.39E-05	0.000588325
ncRNA0326	I-(BB_A66/BB_A68)	456.2996811	1.581317957	0.367259293	4.305726189	1.66E-05	0.000133701
ncRNA0327	I-(BB_A68/BB_A69)	288.5694033	1.264266587	0.411522547	3.072168452	0.002125098	0.009754828
ncRNA0330	p-(BB_Q04)	720.3616324	-1.024359045	0.398542035	-2.570266009	0.010162045	0.036572852
ncRNA0337	I-(BB_Q37/BB_Q38)	248.2040394	1.382053001	0.42404404	3.259220435	0.001117188	0.005696322
rna11	tRNA-Ile-1	3677.251289	-1.070763646	0.431861739	-2.479413086	0.01315988	0.044643975
lcl|NC_001318.1_cds_NP_212138.2_3	BB_0004	3213.612708	-1.161277569	0.350246068	-3.315604869	0.00091445	0.004806509
lcl|NC_001318.1_cds_NP_212142.2_7	cdaA	904.7017596	-1.110337438	0.151425193	-7.332580652	2.26E-13	6.99E-12
lcl|NC_001318.1_cds_NP_212144.1_9	BB_0010	909.552351	-1.051581313	0.245404371	-4.285096096	1.83E-05	0.00014536
lcl|NC_001318.1_cds_NP_212156.1_20	ruvB	2074.85845	-1.615201563	0.202585957	-7.972919689	1.55E-15	5.86E-14
lcl|NC_001318.1_cds_NP_212157.1_21	ruvA	1097.97296	-1.273090208	0.151883284	-8.382029754	5.20E-17	2.39E-15
lcl|NC_001318.1_cds_NP_212160.1_24	BB_0026	1860.316044	-1.010075684	0.181995954	-5.549989787	2.86E-08	4.02E-07
lcl|NC_001318.1_cds_NP_212161.1_25	BB_0027	9710.085949	-2.082180896	0.337466351	-6.170040036	6.83E-10	1.45E-08
lcl|NC_001318.1_cds_NP_212169.1_33	BB_0035	2717.907721	1.16654414	0.214712484	5.433052237	5.54E-08	7.33E-07
lcl|NC_001318.1_cds_NP_212174.1_38	cheR	2192.918259	-1.271798562	0.187794352	-6.772293995	1.27E-11	3.32E-10
lcl|NC_001318.1_cds_NP_212300.1_155	malQ	848.9299321	1.252229151	0.320511286	3.906973652	9.35E-05	0.000646997
lcl|NC_001318.1_cds_NP_212306.2_161	BB_0172	2887.764892	-1.261054254	0.123970769	-10.17219033	2.64E-24	1.61E-22
lcl|NC_001318.1_cds_NP_212374.1_225	glpF	2634.820779	1.096128043	0.214494113	5.110294302	3.22E-07	3.58E-06
lcl|NC_001318.1_cds_NP_212375.1_226	glpK	5175.056984	2.392006594	0.337870592	7.079653128	1.45E-12	4.17E-11
lcl|NC_001318.1_cds_NP_212376.1_227	BB_0242	990.1464708	2.168283941	0.419660091	5.166762312	2.38E-07	2.70E-06
lcl|NC_001318.1_cds_NP_212377.1_228	glpD	6087.037737	2.007251927	0.236394298	8.491118202	2.05E-17	9.68E-16
lcl|NC_001318.1_cds_NP_212419.2_270	BB_0285	4139.079468	-1.005966128	0.317608	-3.16731987	0.00153851	0.00755067
lcl|NC_001318.1_cds_NP_212464.1_313	BB_0330	9233.475103	1.246831348	0.219007994	5.693086005	1.25E-08	1.95E-07
lcl|NC_001318.1_cds_NP_212465.1_314	BB_0331	360.8537922	1.056494139	0.349446428	3.023336497	0.002500041	0.011233693
lcl|NC_001318.1_cds_NP_212477.1_326	gatC	285.3009861	-1.21149949	0.216711395	-5.590382031	2.27E-08	3.24E-07
lcl|NC_001318.1_cds_NP_212498.1_345	mgsA	3019.346339	1.88485369	0.164726999	11.44228752	2.57E-30	2.57E-28
lcl|NC_001318.1_cds_NP_212499.1_346	la7	8401.745644	1.980278708	0.282326106	7.014153714	2.31E-12	6.46E-11
lcl|NC_001318.1_cds_NP_212510.1_357	metK	3414.267219	1.007169116	0.303249798	3.321252387	0.000896145	0.004796807
lcl|NC_001318.1_cds_NP_212530.1_377	rpmG	7700.523929	-1.014284963	0.225205091	-4.503827867	6.67E-06	5.78E-05
lcl|NC_001318.1_cds_NP_212542.1_389	fruA1	6219.62582	1.359858126	0.22210754	6.122521208	9.21E-10	1.84E-08
lcl|NC_001318.1_cds_NP_212549.1_394	BB_0415	1448.604328	-1.279087526	0.222055031	-5.760227649	8.40E-09	1.39E-07
lcl|NC_001318.1_cds_NP_212561.2_403	BB_0427	376.5915461	-1.245177776	0.398443638	-3.12510392	0.001777423	0.00850267
lcl|NC_001318.1_cds_NP_212562.1_404	BB_0428	2667.6549	-1.138481301	0.213578724	-5.330499604	9.79E-08	1.21E-06
lcl|NC_001318.1_cds_NP_212563.1_405	BB_0429	2866.185843	-1.045234832	0.205122092	-5.095671663	3.48E-07	3.84E-06
lcl|NC_001318.1_cds_NP_212564.2_406	BB_0430	199.0192104	-1.022890971	0.324696532	-3.150298416	0.001631038	0.007891072
lcl|NC_001318.1_cds_NP_212568.1_409	BB_0434	410.6547229	-1.042338737	0.306030686	-3.405994191	0.000659236	0.003717479
lcl|NC_001318.1_cds_NP_212574.1_415	rpmH	1315.252463	-1.117891999	0.181344863	-6.164453644	7.07E-10	1.49E-08
lcl|NC_001318.1_cds_NP_212585.1_426	BB_0451	435.9912764	1.007584914	0.20928468	4.814422702	1.48E-06	1.44E-05
lcl|NC_001318.1_cds_NP_212588.1_429	BB_0454	2730.888853	-1.031595834	0.18436356	-5.595443224	2.20E-08	3.21E-07
lcl|NC_001318.1_cds_NP_212599.1_440	BB_0465	2885.545854	-1.007502788	0.230478313	-4.37135613	1.23E-05	0.000100134
lcl|NC_001318.1_cds_NP_212643.1_482	BB_0509	8232.921912	-2.709765829	0.365717385	-7.409453136	1.27E-13	4.15E-12
lcl|NC_001318.1_cds_NP_212667.1_496	BB_0533	5644.777969	-1.062869351	0.106871435	-9.945308127	2.64E-23	1.55E-21
lcl|NC_001318.1_cds_NP_212671.1_500	BB_0537	1848.252264	1.055907202	0.23840089	4.429124408	9.46E-06	7.90E-05
lcl|NC_001318.1_cds_NP_212677.2_505	BB_0543	8778.761317	-1.019087913	0.163819468	-6.220798579	4.95E-10	1.07E-08
lcl|NC_001318.1_cds_NP_212696.1_524	BB_0562	1222.608908	1.053705746	0.14722859	7.156937018	8.25E-13	2.46E-11
lcl|NC_001318.1_cds_NP_212711.1_539	BB_0577	1867.115855	-1.516846079	0.22325663	-6.794181569	1.09E-11	2.90E-10
lcl|NC_001318.1_cds_NP_212722.1_550	BB_0588	1645.236707	-2.280846585	0.1324333	-17.2226063	1.80E-66	5.10E-64
lcl|NC_001318.1_cds_NP_212732.1_560	murB	2605.560826	-1.093452916	0.256548625	-4.262166346	2.02E-05	0.000160363
lcl|NC_001318.1_cds_NP_212737.1_565	p66	18788.96545	1.191518505	0.370846197	3.212972161	0.00131369	0.006618978
lcl|NC_001318.1_cds_NP_212760.1_587	rnmV	17091.35904	-1.622284749	0.410087902	-3.955943936	7.62E-05	0.000536466
lcl|NC_001318.1_cds_YP_008686584.1_588	BB_0627	1365.69883	-1.137219758	0.235678807	-4.825294946	1.40E-06	1.38E-05
lcl|NC_001318.1_cds_NP_212771.2_597	BB_0637	8048.197727	1.247308866	0.261337592	4.772787777	1.82E-06	1.75E-05
lcl|NC_001318.1_cds_NP_212772.1_598	BB_0638	3886.718713	1.374480998	0.209927381	6.547411741	5.85E-11	1.38E-09
lcl|NC_001318.1_cds_NP_212773.1_599	potD	1482.689456	1.111387661	0.287042753	3.871854103	0.000108011	0.000732837
lcl|NC_001318.1_cds_NP_212777.1_603	ylqF	699.0466979	-1.012270773	0.224680411	-4.505380641	6.63E-06	5.78E-05
lcl|NC_001318.1_cds_NP_212778.1_604	nanE	697.3884376	1.091911248	0.199286939	5.479090873	4.28E-08	5.82E-07
lcl|NC_001318.1_cds_NP_212812.1_638	BB_0678	5061.700522	1.130878673	0.184670586	6.123761744	9.14E-10	1.84E-08
lcl|NC_001318.1_cds_NP_212813.2_639	BB_0679	4281.290839	1.092892006	0.192500112	5.677357764	1.37E-08	2.12E-07
lcl|NC_001318.1_cds_NP_212815.2_641	BB_0681	1841.564157	1.132095989	0.198715125	5.697080115	1.22E-08	1.92E-07
lcl|NC_001318.1_cds_NP_212817.1_643	BB_0683	9215.747161	-1.342614394	0.200226812	-6.705467576	2.01E-11	5.18E-10
lcl|NC_001318.1_cds_NP_212818.2_644	fni	5419.350534	-1.360464659	0.310958576	-4.375067179	1.21E-05	9.89E-05
lcl|NC_001318.1_cds_NP_212828.2_653	ffh	14060.76269	-3.018553552	0.374236073	-8.065907513	7.27E-16	2.81E-14
lcl|NC_001318.1_cds_NP_212829.1_654	rpsP	1323.098993	-1.039395173	0.253757071	-4.096024477	4.20E-05	0.000307202
lcl|NC_001318.1_cds_NP_212830.1_655	BB_0696	1648.933208	-1.064165262	0.332285958	-3.202558632	0.001362126	0.006822647
lcl|NC_001318.1_cds_YP_008686588.1_680	cabP	3690.875669	-1.213430799	0.267574041	-4.534934676	5.76E-06	5.06E-05
lcl|NC_001318.1_cds_YP_008686589.1_694	BB_0739	1873.957841	-1.419653077	0.173023273	-8.204983358	2.31E-16	1.01E-14
lcl|NC_001318.1_cds_NP_212899.1_718	BB_0765	561.7600503	1.079796925	0.175287443	6.160149906	7.27E-10	1.51E-08
lcl|NC_001318.1_cds_NP_212900.1_719	cvpA	420.5109223	1.356521949	0.239595391	5.661719703	1.50E-08	2.30E-07
lcl|NC_001318.1_cds_NP_212901.1_720	murG	901.267597	1.560828217	0.281070942	5.553146858	2.81E-08	3.98E-07
lcl|NC_001318.1_cds_NP_212902.1_721	BB_0768	1156.42566	1.034548687	0.234654542	4.408815955	1.04E-05	8.59E-05
lcl|NC_001318.1_cds_NP_212903.1_722	BB_0769	1653.77636	1.261924875	0.240959955	5.237073006	1.63E-07	1.93E-06
lcl|NC_001318.1_cds_NP_212904.1_723	BB_0770	1364.054451	1.297246253	0.244604596	5.303441851	1.14E-07	1.37E-06
lcl|NC_001318.1_cds_NP_212907.1_727	BB_0773	467.79866	1.23579489	0.179319285	6.891589423	5.52E-12	1.49E-10
lcl|NC_001318.1_cds_NP_212919.1_739	spoVG	2664.703766	1.601558347	0.21191112	7.557689027	4.10E-14	1.46E-12
lcl|NC_001318.1_cds_YP_008686594.1_748	BB_0794	8844.212411	-1.00438858	0.174263856	-5.763608144	8.23E-09	1.37E-07
lcl|NC_001318.1_cds_NP_212932.1_752	BB_0798	412.7429471	-1.27472152	0.230756119	-5.524107118	3.31E-08	4.62E-07
lcl|NC_001318.1_cds_NP_212975.1_793	arcA	2690.756525	3.208277689	0.156554928	20.49298436	2.49E-93	1.06E-90
lcl|NC_001318.1_cds_NP_212976.2_794	arcB	2452.381364	2.753000269	0.247532876	11.12175607	9.83E-29	8.37E-27
lcl|NC_001318.1_cds_NP_212977.2_795	BB_0843	9954.801279	2.005127584	0.225005605	8.911456163	5.04E-19	2.68E-17
lcl|NC_001318.1_cds_NP_212985.1_797	BB_0852	258.4724191	-1.108790124	0.362781795	-3.056355468	0.002240455	0.010174651
lcl|NC_001903.1_cds_NP_046990.2_801	chbC	11469.16755	5.577232622	0.223856	24.91437634	5.20E-137	4.43E-134
lcl|NC_001903.1_cds_NP_046991.1_802	chbA	2551.066468	5.136410472	0.275411401	18.64995587	1.26E-77	4.30E-75
lcl|NC_001903.1_cds_NP_046992.2_803	chbB	1687.198719	6.041919219	0.262574866	23.01027252	3.68E-117	2.09E-114
lcl|NC_001903.1_cds_NP_046993.1_804	BB_B07	8409.809781	4.824049857	0.162670624	29.6553228	2.90E-193	4.93E-190
lcl|NC_001903.1_cds_NP_046996.1_806	BB_B10	3773.691125	-1.28185313	0.381829657	-3.357133493	0.000787551	0.004368726
lcl|NC_001903.1_cds_NP_047015.1_821	BB_B29	24118.646	-2.302950375	0.212949666	-10.81452915	2.94E-27	2.18E-25
lcl|NC_000948.1_cds_NP_051171.1_830	BB_P10	89.29313498	-1.80856563	0.568945229	-3.178804458	0.001478838	0.007321109
lcl|NC_000948.1_cds_NP_051176.1_835	BB_P15	57.75190122	-1.511845915	0.533207038	-2.83538252	0.004577085	0.018471031
lcl|NC_000948.1_cds_NP_051187.2_846	BB_P26	172.8371938	1.044255165	0.268808755	3.884751315	0.000102435	0.000697785
lcl|NC_000948.1_cds_NP_051192.1_851	BB_P31	319.4277675	1.343678557	0.331802576	4.04963268	5.13E-05	0.000367062
lcl|NC_000948.1_cds_NP_051193.1_852	BB_P32	812.8429087	1.854983089	0.23580194	7.866699848	3.64E-15	1.35E-13
lcl|NC_000948.1_cds_NP_051194.2_853	BB_P33	507.5530008	2.041103905	0.245742172	8.305875571	9.91E-17	4.44E-15
lcl|NC_000948.1_cds_NP_051195.1_854	bdrA	840.2082301	1.459685689	0.302686653	4.822431625	1.42E-06	1.40E-05
lcl|NC_000948.1_cds_NP_051200.1_859	erpB	7072.921801	1.016940813	0.344628397	2.950832906	0.003169183	0.013359205
lcl|NC_000949.1_cds_NP_051218.1_876	BB_S15	10.07082847	2.315424388	0.806609874	2.870562912	0.004097417	0.016733574
lcl|NC_000949.1_cds_NP_051229.2_886	BB_S26	135.8581982	1.00586234	0.349072328	2.881529873	0.003957497	0.016318687
lcl|NC_000949.1_cds_NP_051234.2_890	BB_S31	105.7674492	-1.268266915	0.504053967	-2.516133186	0.011865034	0.041491072
lcl|NC_000949.1_cds_NP_051238.1_893	BB_S35	265.7310316	1.022944148	0.241100842	4.242806206	2.21E-05	0.000171655
lcl|NC_000949.1_cds_NP_051240.1_894	bdrE	326.736536	1.384619643	0.242365624	5.712937421	1.11E-08	1.78E-07
lcl|NC_000950.1_cds_NP_051272.1_925	BB_R25	73.6903149	1.567845055	0.47257634	3.317654571	0.000907767	0.004806509
lcl|NC_000950.1_cds_NP_051274.2_927	bdrH	752.05999	-1.407429246	0.239268756	-5.882210729	4.05E-09	7.11E-08
lcl|NC_000950.1_cds_NP_051281.1_933	BB_R34	206.0163455	1.062124857	0.219253677	4.844273872	1.27E-06	1.27E-05
lcl|NC_000951.1_cds_NP_051316.1_965	BB_M25	73.66910533	1.56778483	0.472774075	3.316139594	0.000912702	0.004806509
lcl|NC_000952.1_cds_NP_051365.1_1013	BB_O32	381.8142121	1.017080674	0.245240622	4.147276529	3.36E-05	0.000251307
lcl|NC_000952.1_cds_NP_051368.1_1016	BB_O35	19.61631666	1.531544803	0.614609961	2.491897136	0.012706283	0.043592704
lcl|NC_000952.1_cds_NP_051373.1_1021	erpM	506.3123838	1.178636168	0.240991847	4.890771956	1.00E-06	1.03E-05
lcl|NC_000953.1_cds_NP_051387.1_1034	BB_L10	89.29313498	-1.80856563	0.568945229	-3.178804458	0.001478838	0.007321109
lcl|NC_000953.1_cds_NP_051392.1_1039	BB_L15	57.75190122	-1.511845915	0.533207038	-2.83538252	0.004577085	0.018471031
lcl|NC_000953.1_cds_NP_051417.1_1062	erpO	7072.829661	1.016942202	0.344623288	2.950880677	0.003168693	0.013359205
lcl|NC_000954.1_cds_NP_051443.1_1087	BB_N31	161.0855567	1.597055987	0.349207277	4.57337545	4.80E-06	4.30E-05
lcl|NC_000954.1_cds_NP_051444.1_1088	BB_N32	241.9969265	1.728633599	0.308943605	5.595304672	2.20E-08	3.21E-07
lcl|NC_000954.1_cds_NP_051445.1_1089	BB_N33	157.3216497	1.90141208	0.288793883	6.583976291	4.58E-11	1.11E-09
lcl|NC_000954.1_cds_NP_051446.1_1090	bdrQ	227.796319	1.584969312	0.264083749	6.001767683	1.95E-09	3.69E-08
lcl|NC_000954.1_cds_NP_051450.1_1094	erpQ	1968.305688	1.240874035	0.360078608	3.446119836	0.000568698	0.003260919
lcl|NC_001849.2_cds_NP_045397.1_1115	BB_D13	908.2099786	-1.446791877	0.304769578	-4.747166331	2.06E-06	1.94E-05
lcl|NC_001849.2_cds_NP_045398.1_1116	BB_D14	3487.083186	-1.95219011	0.326963037	-5.970675236	2.36E-09	4.39E-08
lcl|NC_001849.2_cds_NP_045399.2_1117	BB_D15	1008.771523	-1.197583135	0.198847084	-6.022633637	1.72E-09	3.32E-08
lcl|NC_001849.2_cds_NP_045404.1_1119	BB_D21	967.6859329	-1.852887349	0.440068742	-4.210449803	2.55E-05	0.000194633
lcl|NC_001849.2_cds_NP_045405.1_1120	BB_D22	128.5625749	-1.396085804	0.318107631	-4.388721519	1.14E-05	9.38E-05
lcl|NC_001849.2_cds_YP_004940417.1_112	BB_D0031	25.13306541	-2.332828324	0.700631412	-3.329608525	0.000869682	0.004731846
lcl|NC_001851.2_cds_NP_045439.1_1144	repU	189.0840177	1.753939584	0.35530882	4.936380643	7.96E-07	8.47E-06
lcl|NC_001851.2_cds_NP_045456.1_1151	BB_F23	367.9227399	2.20505297	0.256947467	8.581726827	9.35E-18	4.55E-16
lcl|NC_001851.2_cds_NP_045458.1_1153	BB_F25	156.0679977	1.059224925	0.435755106	2.430780295	0.015066347	0.04943736
lcl|NC_001852.1_cds_NP_045472.1_1163	BB_G12	81.95387397	-2.20532043	0.670605133	-3.288552864	0.001007039	0.00519693
lcl|NC_001852.1_cds_NP_045482.1_1174	BB_G22	64.42227065	-3.125608056	0.641815831	-4.869945401	1.12E-06	1.13E-05
lcl|NC_001852.1_cds_NP_045484.2_1176	BB_G24	50.67291727	-1.450408152	0.561382788	-2.583634878	0.009776523	0.035651861
lcl|NC_001852.1_cds_NP_045485.1_1177	BB_G25	3.961622236	-2.090912702	0.854820598	-2.446025174	0.014444091	0.047594109
lcl|NC_001852.1_cds_NP_045489.1_1181	BB_G29	357.369219	-1.009105265	0.377296002	-2.674571849	0.007482474	0.028443423
lcl|NC_001852.1_cds_NP_045491.2_1183	BB_G31	41.00949741	-1.490324525	0.469590481	-3.173668518	0.001505255	0.007430287
lcl|NC_001853.1_cds_NP_045510.1_1195	BB_H17	10.29555978	-2.148674895	0.800721139	-2.683424715	0.007287238	0.027825485
lcl|NC_001853.1_cds_NP_045517.1_1197	BB_H26	1195.446691	-1.144943567	0.461056964	-2.483301754	0.013017077	0.044247668
lcl|NC_001855.1_cds_NP_045575.1_1225	BB_K01	1354.450581	1.456025551	0.334102075	4.358026067	1.31E-05	0.000105926
lcl|NC_001855.1_cds_NP_045597.1_1234	BB_K23	2453.836139	-1.425251086	0.386783132	-3.684884288	0.000228807	0.001481588
lcl|NC_001855.1_cds_NP_045598.1_1235	BB_K24	377.5769784	-1.452422742	0.43695597	-3.323956741	0.0008875	0.004792717
lcl|NC_001855.1_cds_NP_045606.1_1238	BB_K33	37.62541137	-1.381941183	0.444818197	-3.106755056	0.001891531	0.008898554
lcl|NC_001855.1_cds_NP_045612.1_1242	BB_K40	3811.74482	-1.258348118	0.231710329	-5.430694971	5.61E-08	7.35E-07
lcl|NC_001855.1_cds_NP_045618.1_1246	BB_K47	2505.671645	1.677219339	0.317042319	5.290206506	1.22E-07	1.47E-06
lcl|NC_001855.1_cds_NP_045620.1_1248	BB_K49	1392.93963	1.632033985	0.354485887	4.603946289	4.15E-06	3.76E-05
lcl|NC_001855.1_cds_NP_045624.1_1252	BB_K53	359.8395733	-1.240359481	0.484313307	-2.561068345	0.010435083	0.037333919
lcl|NC_001856.1_cds_NP_045633.1_1254	BB_J09	24951.96368	1.396908601	0.377302455	3.70235757	0.000213605	0.001388434
lcl|NC_001856.1_cds_NP_045642.1_1259	BB_J18	652.4014099	-1.21353758	0.493482874	-2.459128057	0.013927493	0.046598272
lcl|NC_001856.1_cds_NP_045643.1_1260	BB_J19	8721.994493	-1.178524274	0.357433879	-3.297181218	0.000976605	0.005055191
lcl|NC_001856.1_cds_NP_045648.1_1264	BB_J24	96.33456458	-1.446657884	0.37915883	-3.815440313	0.00013594	0.000911442
lcl|NC_001856.1_cds_NP_045660.1_1272	BB_J36	1399.444538	-1.03853811	0.235436708	-4.411113785	1.03E-05	8.54E-05
lcl|NC_001856.1_cds_NP_045661.2_1273	BB_J37	15.94581387	-1.809580589	0.694226935	-2.606612476	0.009144279	0.033489691
lcl|NC_001856.1_cds_NP_045667.1_1276	BB_J43	43.39693922	-1.683539357	0.669523881	-2.514532201	0.011919042	0.041513166
lcl|NC_001856.1_cds_NP_045669.1_1277	BB_J45	396.4427063	-1.389596423	0.387960567	-3.5817981	0.000341237	0.002120903
lcl|NC_001857.2_cds_NP_045676.1_1287	BB_A03	12443.29488	2.365156998	0.336486898	7.028972045	2.08E-12	5.91E-11
lcl|NC_001857.2_cds_NP_045677.1_1288	BB_A04	322.0576528	1.186683617	0.439379821	2.700815014	0.00691698	0.026711149
lcl|NC_001857.2_cds_NP_045696.2_1302	BB_A23	697.3262104	-1.509462463	0.587335071	-2.570019291	0.010169285	0.036572852
lcl|NC_001857.2_cds_NP_045697.1_1303	dbpA	424.4061803	-1.294172188	0.532360559	-2.431006891	0.015056928	0.04943736
lcl|NC_001857.2_cds_NP_045703.1_1305	BB_A30	869.6149934	1.582224773	0.216857972	7.296133785	2.96E-13	9.01E-12
lcl|NC_001857.2_cds_NP_045704.1_1306	BB_A31	693.6564221	1.993259822	0.268904006	7.412533024	1.24E-13	4.14E-12
lcl|NC_001857.2_cds_NP_045705.1_1307	BB_A32	110.7421883	1.110559517	0.324430366	3.42310595	0.000619099	0.003514421
lcl|NC_001857.2_cds_NP_045707.1_1309	BB_A34	190.0838372	-1.75905884	0.577174012	-3.047709707	0.002305926	0.010416423
lcl|NC_001857.2_cds_NP_045710.1_1311	BB_A37	93.94411642	-1.86769252	0.652655129	-2.861683662	0.004213973	0.017127436
lcl|NC_001857.2_cds_NP_045725.1_1323	BB_A52	1613.061442	1.078698677	0.31275508	3.449020483	0.000562624	0.003259009
lcl|NC_001857.2_cds_NP_045726.1_1324	BB_A53	375.6912082	1.380553161	0.375841734	3.673230076	0.000239504	0.001533364
lcl|NC_001857.2_cds_NP_045727.1_1325	BB_A54	599.9624458	1.952102454	0.367103617	5.317578918	1.05E-07	1.29E-06
lcl|NC_001857.2_cds_NP_045731.1_1327	BB_A58	8350.064089	1.319104334	0.296677938	4.446250175	8.74E-06	7.37E-05
lcl|NC_001857.2_cds_NP_045733.1_1329	BB_A60	1031.73146	1.630327073	0.221726805	7.352864153	1.94E-13	6.12E-12
lcl|NC_001857.2_cds_NP_045739.1_1334	BB_A66	625.4863294	1.009382205	0.338610772	2.980951255	0.002873545	0.012388981
lcl|NC_001857.2_cds_NP_045747.1_1339	osm28	12273.60539	2.102644849	0.394215493	5.333744837	9.62E-08	1.20E-06
lcl|NC_000956.1_cds_NP_051469.1_1342	BB_Q05	100.9367965	1.796704482	0.595838392	3.015422481	0.002566215	0.011410613
lcl|NC_000956.1_cds_NP_051489.1_1361	BB_Q27	184.692011	2.143457033	0.446777313	4.797595967	1.61E-06	1.56E-05
lcl|NC_000956.1_cds_NP_051491.1_1363	BB_Q29	93.20450698	1.487257023	0.362277235	4.105300802	4.04E-05	0.000296402
lcl|NC_000956.1_cds_NP_051494.1_1366	BB_Q32	73.6825877	1.568087947	0.471959354	3.322506342	0.000892126	0.004792717
lcl|NC_000956.1_cds_NP_051502.1_1373	BB_Q40	481.1703282	1.262062785	0.368213247	3.427532266	0.000609094	0.003469187
lcl|NC_000956.1_cds_NP_051504.1_1374	bdrV	634.196218	2.372377131	0.317623917	7.469138837	8.07E-14	2.81E-12
lcl|NC_000956.1_cds_NP_051521.1_1385	BB_Q62	20.48709362	3.14255226	0.535260529	5.871070419	4.33E-09	7.52E-08

**Fig 4:**
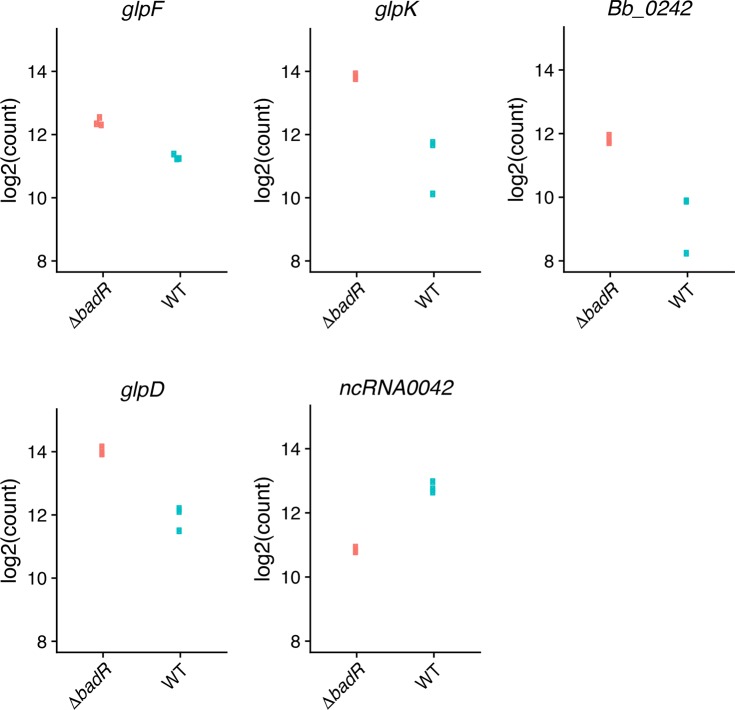
RNA-Seq analyses of expression of the *glpFKD* operon in the *badR* mutant. Log2 transformed counts of transcripts encoded from the *glpFKD* operon and the anti-*glpF* small ncRNA (*ncRNA0042*) in *badR* mutant and wild-type. Three replicates were assessed. Some values were essentially identical, and so appear to be single dots in the figures.

Glycerol is a key nutrient for *B*. *burgdorferi* during tick colonization, and mutants unable to metabolize that carbohydrate are significantly impaired [70]. The operon encoding import and catabolism of glycerol consists of four genes *glpF*, *glpK*, an ORF of unknown function (ORF *BB_0242*), and *glpD* [18, 70, 71]. The *glpFKD* operon appears to be under complex control, being reported to be impacted by several regulatory factors, including RpoS, SpoVG, cyclic-di-GMP, and ppGpp [70, 72–74]. In addition, an antisense RNA that is transcribed within the *glpF* ORF, *ncRNA0042*, was recently identified in two independent studies [30, 50]. Upon deletion of *badR*, *glpFKD* operon transcript levels increased 2- to 4-fold, and *ncRNA0042* was reduced 3.6-fold (Fig 4). Thus, both BadR and *ncRNA0042* need to be considered along with the other proteins and small molecules in future studies on the mechanism controlling borrelial glycerol utilization.

**Fig 5:**
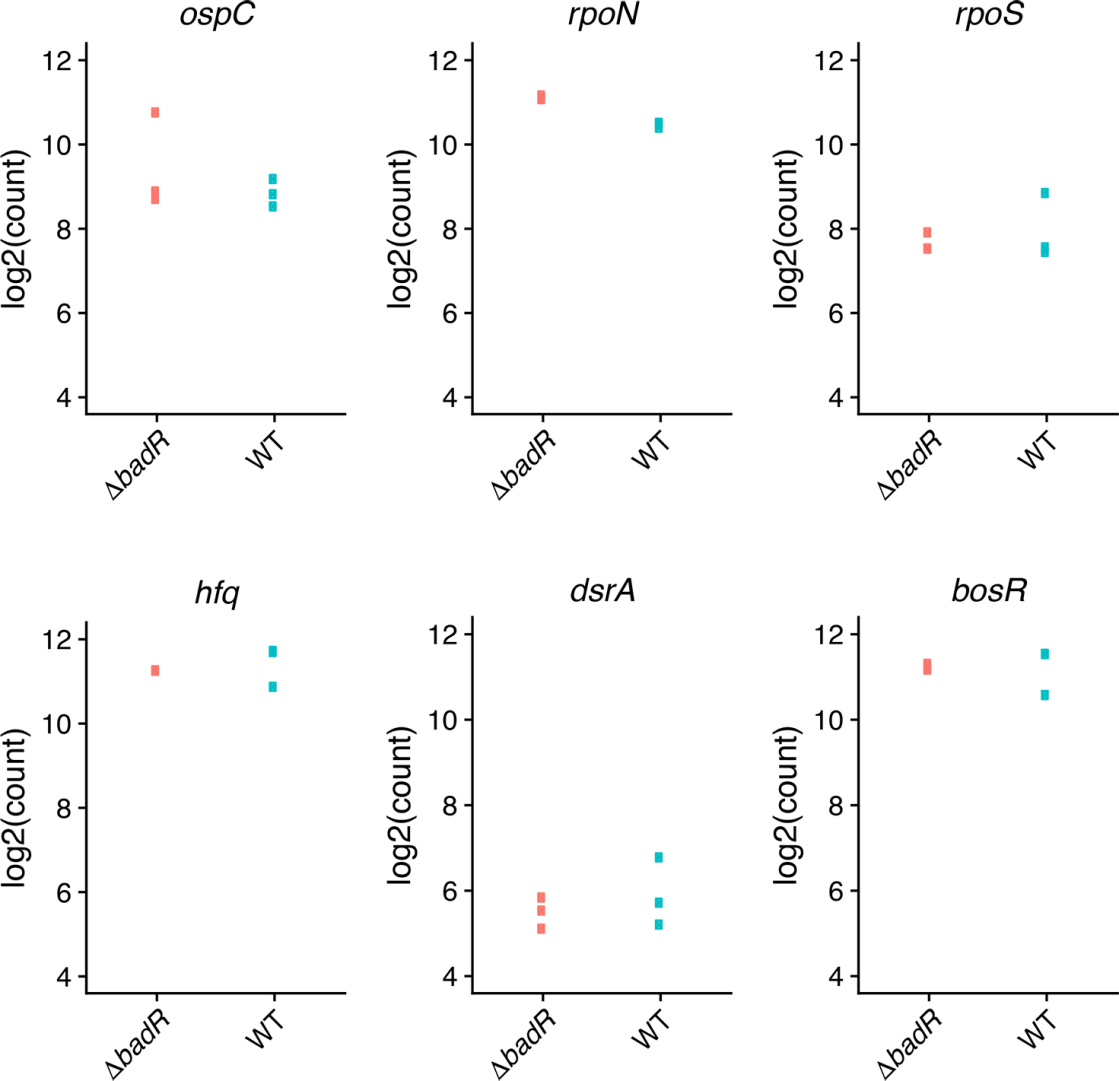
Log2 transformed counts of RNA-Seq results of select transcripts in *badR* mutant and wild-type *B*. *burgdorferi*. Three replicates were assessed. Some values were essentially identical, and so appear to be single dots in the figures.

A previous study found that *badR* deletion had a significant effect upon RpoS expression [16]. In contrast, the conditions utilized in our studies did not show any significant differences in *rpoS* mRNA levels between the *badR* mutant and its wild-type parent. Transcripts of *rpoS* were readily detected in both strains. Additionally, there were no significant changes in the expression levels of any of the known regulators of *rpoS* (*dsrA*, *hfq*, *bosR* or *rpoN*) or RpoS-affected transcripts such as *ospC* (Fig 5). Possible reasons for these results are discussed below.

### The CsrA and BadR regulons share substantial overlap

**Fig 6:**
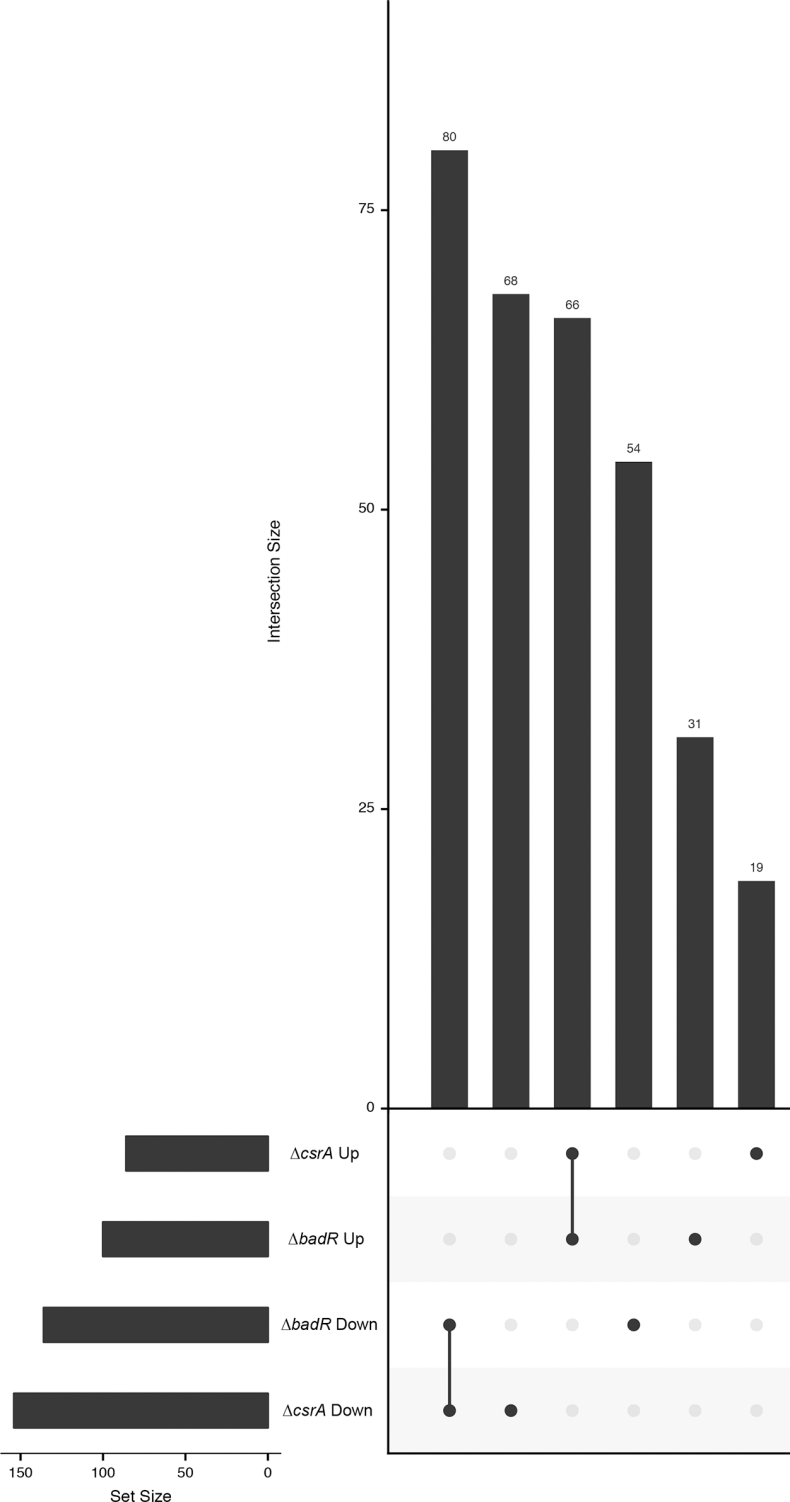
Set analysis of differentially expressed gene sets. The differentially expressed transcripts from either the *csrA* or *badR* mutant vs. wild-type parent were compared for any overlap in identity using the UpsetR package. Affected transcript sets were first filtered by higher or lower abundance to create 8 possible sets (Four regulators, at higher or lower abundance). The horizontal bars on the lower left side indicate the number of transcripts in each set (e.g. 153 transcripts were significantly reduced in the *csrA* mutant). Dark dots indicate each set, and dark dots connected by a line indicate paired sets (e.g. the leftmost dots and line refer to the paired set of transcripts that were reduced in both the *csrA* and *badR* mutants). The vertical bars indicate the number of transcripts in each set or paired set (e.g. 80 transcripts were reduced in both the *csrA* and *badR* mutants, 68 transcripts were reduced only in the *badR* mutant, 66 transcripts were elevated in both the *csrA* and *badR* mutants, etc.).

**Fig 7:**
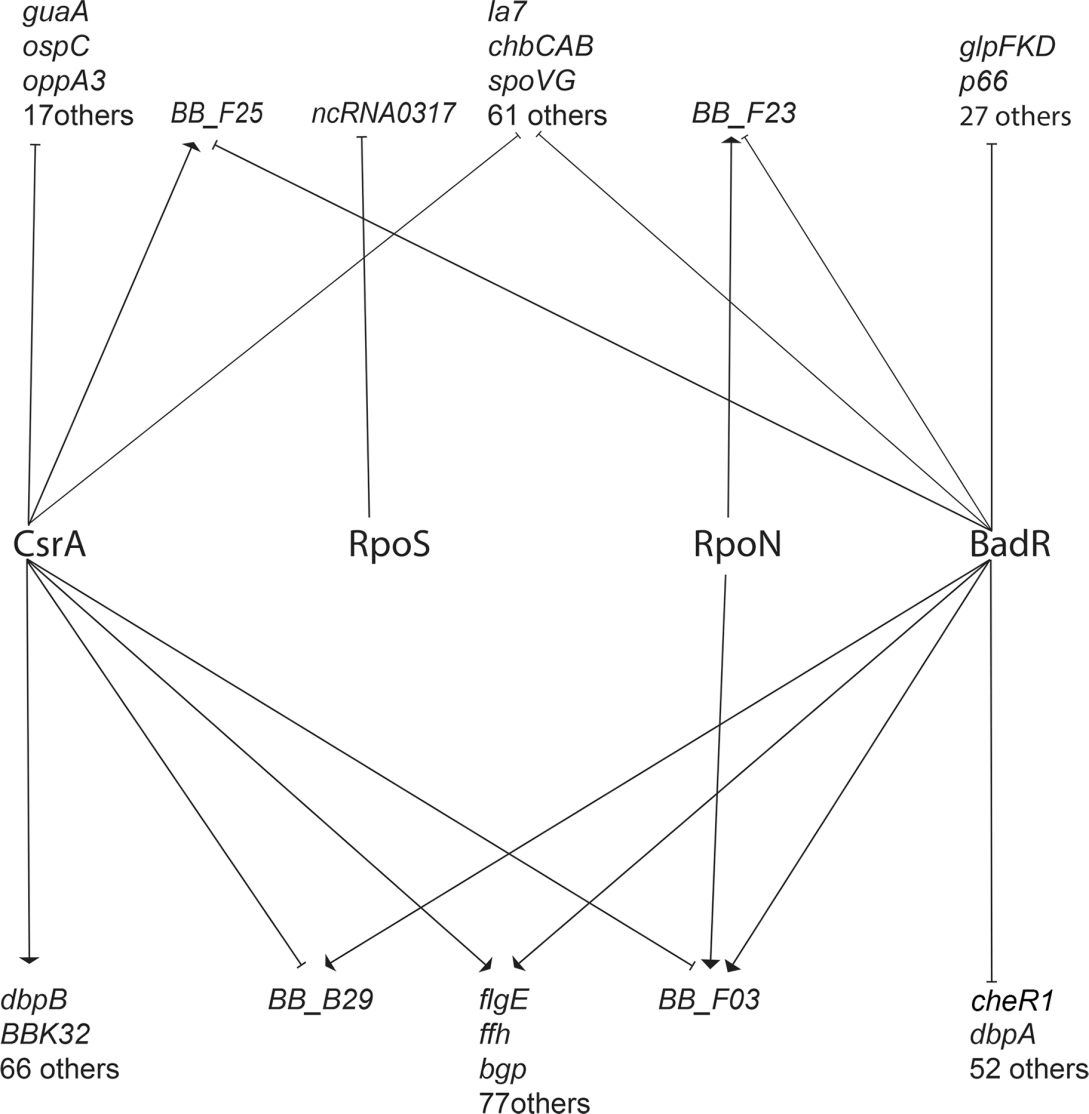
Interaction network of four essential regulatory factors. An interaction network was generated using Adobe Illustrator using the data in S5–S8 Tables. Transcripts that were found in higher abundance by the deletion of a particular factor are indicated by a blocked line stretching from the regulator to the transcript as the regulatory factor would be expected to naturally lower transcript abundance. Those that were found in lower abundance follow the same scheme but are indicated by arrows as the regulatory factor would be expected to naturally increase transcript abundance.

To better understand how these two regulatory factors interact, we analyzed their differentially-expressed transcript sets for intersection and divergence (Figs 6 and 7 and S4 Table). As noted above, deletion of either regulatory factor did not have any detectable impact on expression of the other. Compared with the wild-type parent, the Δ*csrA* and Δ*badR* mutants showed significant changes in the same 150 transcripts (S4 Table). Of these, 80 transcripts were reduced in both mutants, 66 were increased in both mutants, 1 was reduced in the *rpoN*, *csrA*, and *badR* mutants, and only 3 were affected in opposite directions (i.e. reduced in the *badR* mutant while increased in the *csrA* mutant, or vice versa), 1 of which was also at reduced abundance in the *rpoN* mutant (S4 Table). In contrast, the set of transcripts affected by CsrA alone consisted of 19 increased members and 68 reduced transcripts (Tables 1 and 2 and S4 Table), and, of the transcripts impacted by BadR alone, 53 were reduced and 31 were increased (Tables 3 and 4 and S4 Table).

**Table 3:** Differentially expressed transcript when comparing the rpoS mutant to wild-type. The included transcript met the criteria of >1 log2 fold-change and an adjusted p-value (padj) when comparing the *rpoS* mutant to wild-type. One transcript was impacted, not including the mutated gene, by the mutation. The first column contains the CDS/custom transcript ID which is the transcript ID for all coding sequences obtained from the NCBI Gene file format file or the transcript ID given to ncRNAs. RefSeq entries are further separated by the character “_”. The first portion gives the genetic element from which it is derived, the second describes the type of element (CDS), the third provides RefSeq ID, and the fourth provides a number indicating the particular entries ordered number in the RefSeq entry. The second column is the gene information, for the ncRNAs it contains the location relative to other genes and for predicted or known genes it contains gene name. The remaining columns describe the various metrics of expression of each impacted transcript including, base mean (average library size normalized counts across all samples), log2FC (Fold change estimate), lfcSE (uncertainty of the log fold change estimate), stat (Wald statistic), pvalue, padj (pvalue following Benjamini-Hochberg adjustment). The ncRNA is listed according to the nomenclature the previous analyses of the strain B31 ncRNA transcriptome [30].

RefSeq CDS/Custom Transcript ID	Gene Name	baseMean	log2FoldChange	lfcSE	stat	pvalue	padj
ncRNA0317	I-(BB_A16/BB_A18)	934.0496735	1.303422865	0.279366013	4.665645805	3.08E-06	0.002619633

**Table 4:** Differentially expressed transcripts when comparing the rpoN mutant to wild-type. The included transcripts met the criteria of >1 log2 fold-change and an adjusted p-value (padj) when comparing the *rpoN* mutant to wild-type. A total of 6 transcripts were differentially regulated, not including the mutated gene, by the mutation. The first column contains the CDS/custom transcript ID which is the transcript ID for all coding sequences obtained from the NCBI Gene file format file or the transcript ID given to ncRNAs. RefSeq entries are further separated by the character “_”. The first portion gives the genetic element from which it is derived, the second describes the type of element (CDS), the third provides RefSeq ID, and the fourth provides a number indicating the particular entries ordered number in the RefSeq entry. The second column is the gene information, for the ncRNAs it contains the location relative to other genes and for predicted or known genes it contains gene name. The remaining columns describe the various metrics of expression of each impacted transcript including, base mean (average library size normalized counts across all samples), log2FC (Fold change estimate), lfcSE (uncertainty of the log fold change estimate), stat (Wald statistic), pvalue, padj (pvalue following Benjamini-Hochberg adjustment). ORFs and ncRNAs are identified according to the names or numbers assigned to genes and transcripts by the initial genome sequencing of *B*. *burgdorferi* strain B31 [18, 31] or from our previous analyses of that strain’s ncRNA transcriptome [30].

RefSeq CDS/Custom Transcript ID	Gene Name	baseMean	log2FoldChange	lfcSE	stat	pvalue	padj
ncRNA0247	A-(BB_F03)	1699.002338	-1.505899416	0.295702788	-5.09261149	3.53E-07	4.82E-05
ncRNA0251	I-(BB_F11a/BB_F12)	727.7453318	-1.186871298	0.186813665	-6.353235984	2.11E-10	3.46E-08
lcl|NC_001318.1_cds_NP_212583.1_424	BB_0449	973.8721962	1.053792918	0.264341728	3.986479644	6.71E-05	0.006869549
lcl|NC_001851.2_cds_NP_045439.1_114	repU	189.0840177	-1.381674328	0.377685357	-3.658268196	0.000253925	0.0226871
lcl|NC_001851.2_cds_NP_045453.2_115	BB_F20	136.227553	-1.216027769	0.244677301	-4.969924738	6.70E-07	8.44E-05
lcl|NC_001851.2_cds_NP_045456.1_115	BB_F23	367.9227399	-1.211569926	0.247841487	-4.888487162	1.02E-06	0.000118961

Of the 80 transcripts that were decreased in both mutants, there was a slight bias towards the plasmids, with 56.3% of transcripts being plasmid encoded. Approximately half of the dually reduced transcripts (52.5%) were putative ncRNAs. Conversely, the vast majority of the 67 dually increased transcripts were transcripts encoding ORFs, with only 10 putative ncRNAs (14.9%). The bias for plasmid/chromosomal origin was similar to the ratio for reduced transcripts, with 62.6% of increased transcripts originating from the plasmids.

BadR and CsrA are known nucleic acid-binding proteins and could mediate all of the DE transcript changes directly. However, both mutants exhibited altered expression of *spoVG*, which encodes a regulatory protein with site-specific DNA-/RNA-binding activity [74–76]. The mRNA encoding SpoVG was significantly increased by 3-fold in the *badR* mutant and 2.3-fold in the *csrA* mutant. It is possible that some of the BadR- and/or CsrA-affected ORFs of unknown function also encode nucleic acid-binding proteins.

**Fig 8:**
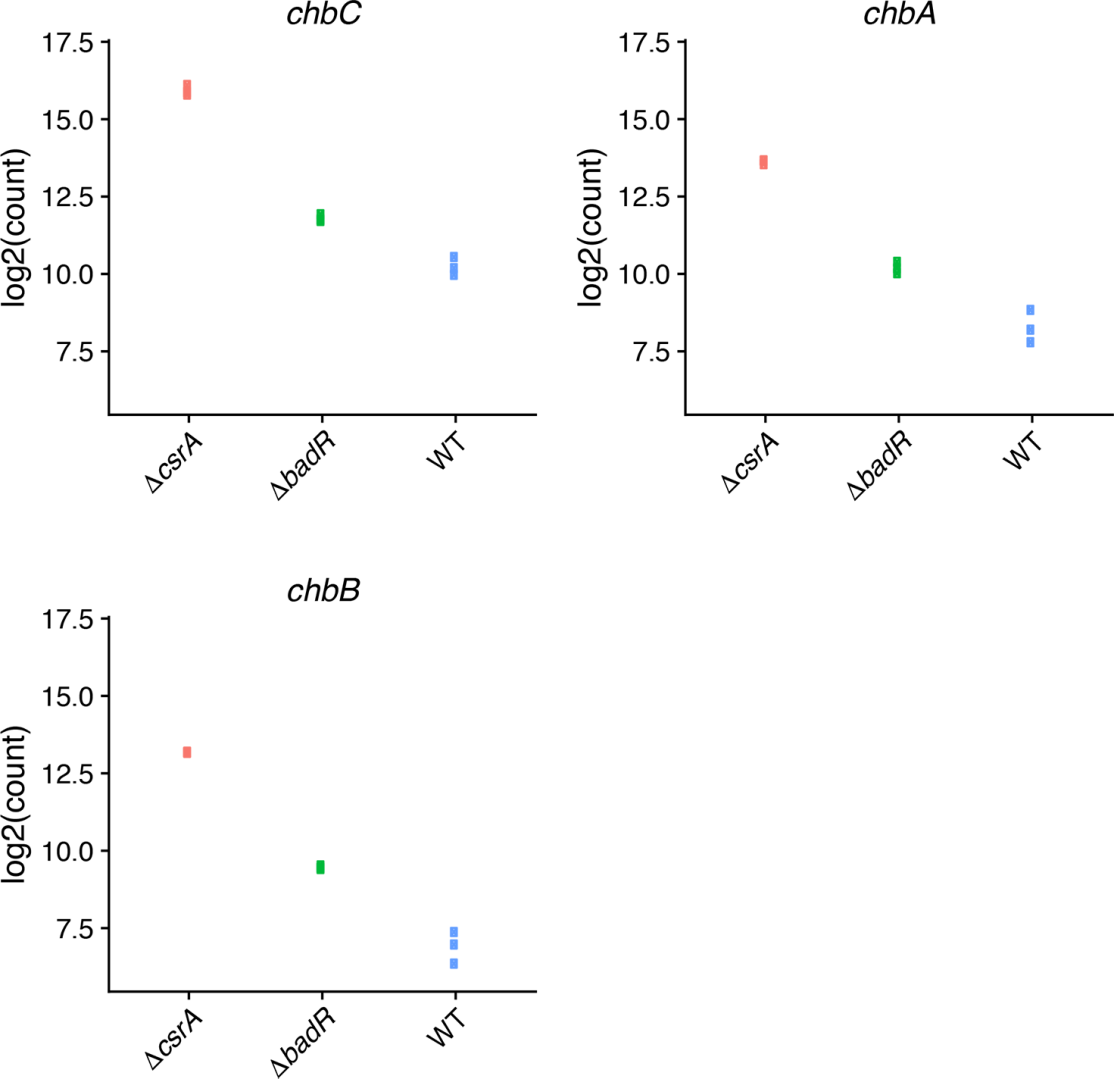
Expression of chitobiose metabolism transcripts affected in *badR* and *csrA* mutants. Log2 transformed counts of transcripts encoded from the *chbA* and *chbB* loci in the *badR*, *csrA*, and wild-type strains. Three replicates were assessed. Some values were essentially identical, and so appear to be single dots in the figures.

Transcripts encoding proteins that are involved in chitobiose uptake (*chbCAB*) were significantly enhanced by 35- to 65-fold in the *badR* mutant, as was previously observed [16]. Moreover, those transcripts were among the most highly increased transcripts in that data set (Fig 8). Deletion of *csrA* also increased the expression of *chbCAB*, by 2.8- to 5.2-fold. Chitobiose is a dimer of N-acetylglucosamine that can be used as an energy source. It is also required for peptidoglycan synthesis [77, 78]. Transcripts encoding other proteins involved with cell wall synthesis were also affected by deletion of either *csrA* or *badR*. The *nanE* transcript, encoding the epimerase that converts N-acetylmannosamine-6-phosphate into GlcNAc-6P, was increased 2.1-fold by the absence of *badR*. MurG is a key enzyme involved in the formation of the peptidoglycan cell wall, transferring GlcNAc moieties from lipid intermediate I to lipid intermediate II; *murG* transcript levels were increased in both the *badR* and *csrA* mutants, by 3-fold and 2.6-fold, respectively.

Other carbohydrate-utilization pathways affected by both the *badR* and *csrA* mutants include increased levels of transcripts encoding a putative hexose transporter IIABC component (*BB_0408*) (2.5-fold and 2.8-fold, respectively) and a subunit of another putative hexose/pentose ABC transporter (*BB_0678*) (2.2-fold and 2.9-fold, respectively).

Several transcripts involved in the uptake and catabolism of polyamines were significantly increased by deletion of either *badR* or *csrA*. Polyamines are cationic organic bases that are present in significant levels within vertebrate hosts, can affect a wide variety of biological processes, and are often involved in stress and osmotic responses. The polyamine uptake system in *B*. *burgdorferi*, and the polyamines spermine and spermidine, are important for control of bacterial growth and expression of infection-associated proteins [79, 80]. Polyamines can be imported through the PotABCD transporter or produced de novo from arginine. *B*. *burgdorferi* possess part of the arginine deaminase pathway, consisting of the enzymes ArcA to convert arginine to citrulline and ArcB to convert citrulline to ornithine and carbamoyl-phosphate [18, 80]. The *badR* and *csrA* mutants both exhibited increased expression of *arcA* (9.2-fold and 2-fold, respectively), *arcB* (6.7-fold and 2.2-fold, respectively), and *potD* (2.1-fold and 2.3-fold, respectively).

**Fig 9:**
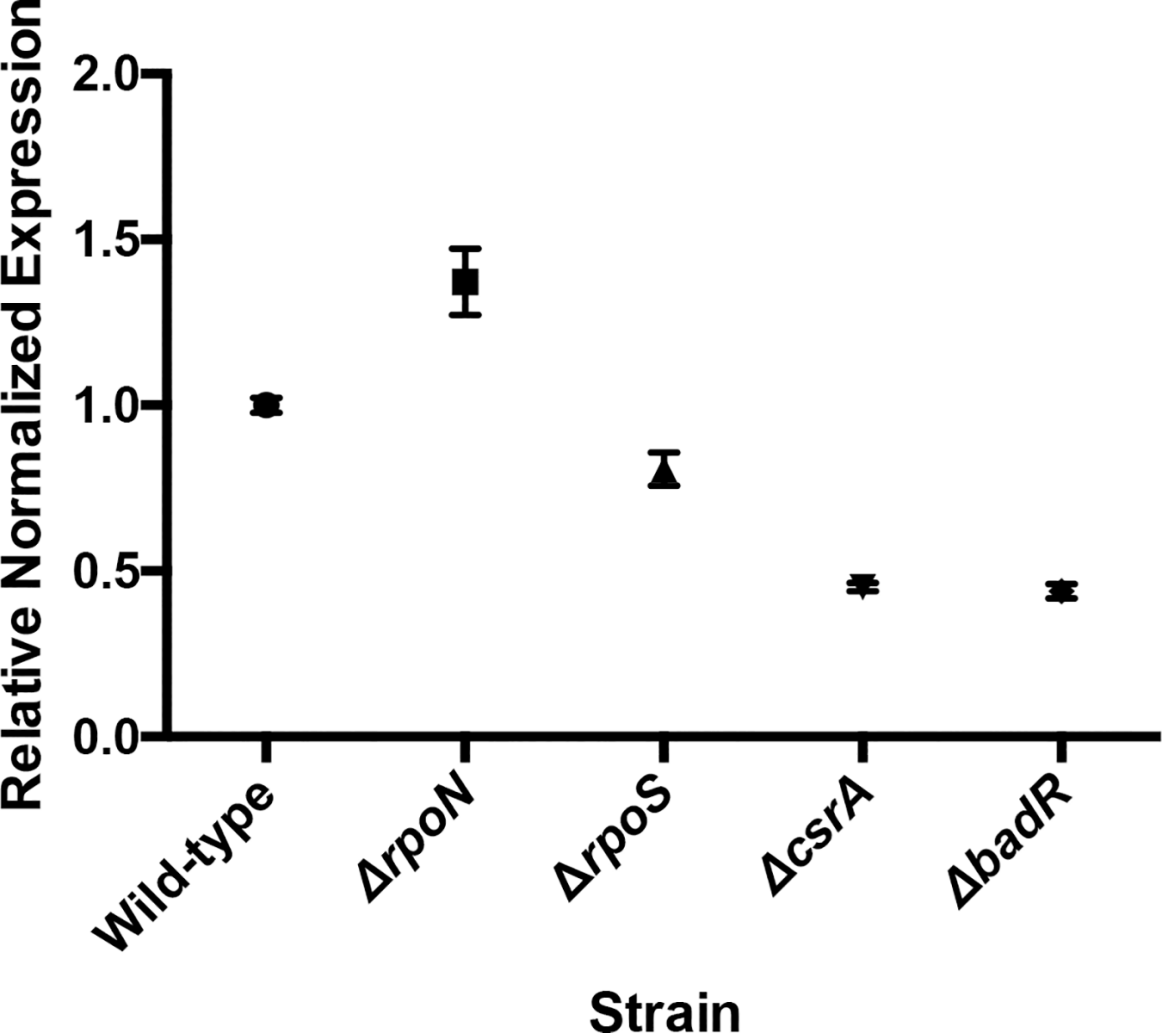
Relative plasmid copy number as assayed by qPCR from mutant and wild-type strains. Genomic DNA was isolated from all five strains and qPCR was performed targeting the plasmid lp28-4 and the chromosome. Three biological replicates of each strain were assayed. Relative copy number of lp28-4 per chromosome was determined using the ΔCt method.

Most transcripts from lp28-4 appeared to be decreased in both the *badR* and *csrA* mutants. To further examine these observations, we isolated genomic DNA from all 5 strains, then used qPCR to examine the copy numbers of lp28-4 relative to the chromosome. We found that the ratio of lp28-4 to chromosome was reduced in the *csrA* (0.45:1) and *badR* (0.43:1) mutants, but that relative abundance also fluctuated in the *rpoN* (1.37:1) and *rpoS* (0.81:1) mutants (Fig 9). *B*. *burgdorferi* has one of the most complex known bacterial genomes, and smaller replicons/plasmids may occasionally be lost during cultivation. As was performed for all of the strains used in the current studies, it is common practice to examine the replicon profile of *B*. *burgdorferi* strains by use of plasmid-specific PCR. At least three hypotheses could explain the results on lp28-4 transcript levels: either those initially-clonal cultures now contain a mixture of bacteria with and without the plasmid, many transcripts from lp28-4 are under the control of BadR and CsrA, or BadR and CsrA have an influence on lp28-4 copy number. Considering the apparent variation in plasmid copy number we opted not to make any further inferences regarding transcripts from genes on lp28-4. Plasmid lp28-4 is not necessary for mammalian infection, although it has some role in tick colonization, so further investigation of these results is warranted [32, 81, 82]. No other borrelial replicon exhibited such a broad expanse of DE transcripts, so the current study did not evaluate copy numbers of the other naturally-occurring plasmids.

### *ospC* and *dbpBA* can be transcribed independently of either alternative sigma factor

RpoN and RpoS are the only alternative sigma factors of *B*. *burgdorferi*, and both are essential for transmission from ticks to vertebrates and for establishment of vertebrate infection [6, 9]. For this reason, we chose deletion mutants in these two sigma factors as an early step in dissecting the gene regulatory networks important for pathogenesis. It is well established that many regulatory factors of *B*. *burgdorferi* are controlled by environmental stimuli [3, 4, 36, 83–88]. In order to control for such potential effects, we performed transcriptome analysis of a single, specific culture condition: mid-exponential phase at 34°C. Both *rpoS* and *rpoN* mRNAs were readily detected at significant levels in the wild-type parental strain under these growth conditions.

Previous immunoblot- or array-based analyses of *rpoS* mutants, cultured under conditions that otherwise induce high-level expression of RpoS, observed that levels of numerous transcripts, such as *ospC* and *dbpBA*, were substantially affected by the *rpoS* mutation [5, 6, 8]. This led to hypotheses that RpoS-RNA polymerase holoenzyme might directly transcribe all of those genes. However, under the current culture conditions, *ospC*, *dbpBA*, and other transcripts were produced at detectable levels in the *rpoS* mutant. Moreover, all of those transcripts were expressed at approximately equivalent amounts in wild-type and *rpoS* mutant samples. These results, combined with those presented below for the *rpoN* mutant, indicate that the *ospC*, *dbpB*, *dbpA*, and many other genes can be transcribed by RNA polymerase using the “housekeeping” sigma, RpoD. We validated these studies with qRT-PCR on samples harvested from independent cultures, which also readily detected *ospC* and *dbpBA* transcripts in the *rpoS* mutant.

Overall, almost no transcripts were detectably impacted by deletion of *rpoS* under the studied culture conditions. Only 1 transcript, *ncRNA0317*, was detected as DE (Fig 1C and Table 3). This transcript is a putative small RNA that is encoded downstream of the *ospB* ORF, in an intergenic location. While RpoS protein production is also controlled post-transcriptionally, none of the transcripts of known regulators DsrA, Hfq, or BBD18, were observed to be affected in either the *rpoS* deletion mutant or any of the other examined mutants.

Deletion of *rpoN* resulted in very few significant changes (Fig 1D and Table 4). Transcript levels of *BB_0449*, the gene that is divergently-transcribed from *rpoN*, were approximately twice as high in the mutant, possibly due to transcriptional read-through from the inserted antibiotic resistance gene. The only other significant difference was the reduced expression of several transcripts on lp28-1, including *BB_F23*, which encodes a putative partition protein. Considering these data, it is possible that *rpoN* could affect the replication of lp28-1. Notably, none of the transcripts previously hypothesized to require RpoS, such as *ospC* or *dbpBA*, were affected by deletion of *rpoN*.

**Fig 10:**
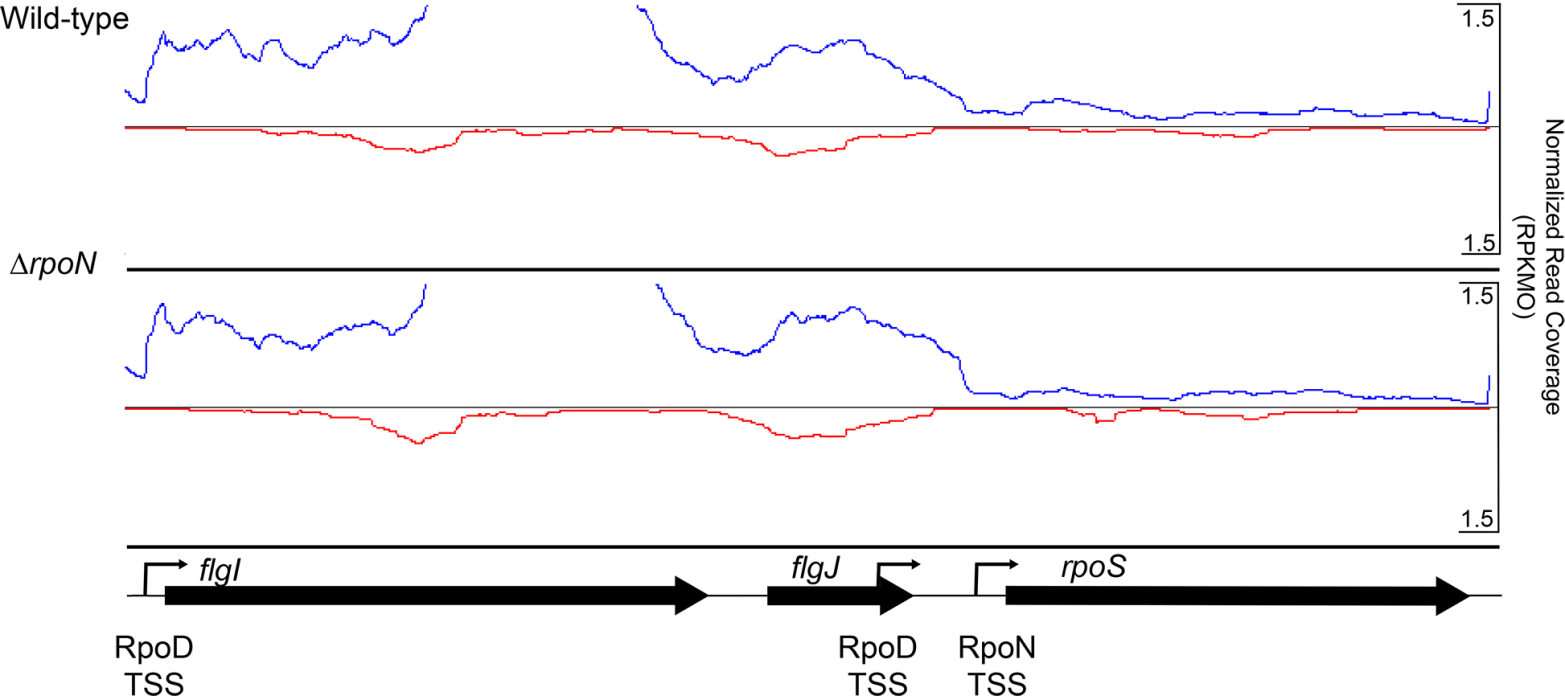
Promoter utilization of *rpoS* in the Δ*rpoN* mutant. Read coverage histograms of *rpoS* locus and upstream genomic region in wild-type and Δ*rpoN* strains. Abundance plots represent the merged normalized expression from three independent biological replicates. Blue lines indicate relative transcript abundance from left to right (the coding strand of *flgI*, *flgJ* and *rpoS*) and red lines indicate relative transcript abundance from the opposite strand (right to left), and reside above (+) and below (-) the central axis. Open reading frames are indicated below coverage plots and direction of transcription is given by arrows at the ends of genes. Transcriptional start sites of the two previously mapped promoters are indicated by “TSS” and arrows. Normalized read coverage of each strand is given as RPKMO (reads per kb of gene per million reads aligning to annotated ORFs) is given on Y-axis on the left. The RpoN- and RpoD-dependent transcriptional start sites were previously identified [1, 11, 90]. Figures were generated in the Artemis Genome Viewer and edited in Adobe Illustrator.

The *rpoS* locus has previously been shown to have both an RpoN- and an RpoD-dependent promoter [1, 11, 89], leading us to investigate read coverage plots for the region around *rpoS* in the wild-type and *rpoN* mutants (Fig 10). These analyses highlighted that transcription initiating from the *flgI* and *flgJ* genes that are directly 5’ of *rpoS* appears to continue into the *rpoS* ORF [30, 90]. Consistent with that observation, the previously-mapped RpoD-dependent promoter of *rpoS* is located within the *flgJ* ORF [11]. Altogether, these results indicate that essentially all transcription of *rpoS* under the assayed culture conditions resulted from RNA polymerase-RpoD holoenzyme using the previously-mapped promoter within *flgJ* and/or by read-through from the promoter 5’ of *flgIJ*. The read coverage plots indicated that substantial amounts of transcripts from *flgJ* were terminated in the space between the *flgJ* and *rpoS* ORFs (Fig 10). No intrinsic (Rho-independent) terminators are present in this region [30], implying that some other type of transcriptional regulatory element exists immediately 5’ of the *rpoS* ORF.

## Discussion

In an effort to determine whether there are overlaps between borrelial regulons, we simultaneously examined wild-type and pairs of mutant *B*. *burgdorferi* that were grown under a single, specific condition, so that each batch of RNA-Seq data sets could be compared with each other. Several important conclusions can be drawn from these results: Both CsrA and BadR affect levels of numerous transcripts, evidently independently of RpoS; the CsrA and BadR regulons include transcripts affected by only one of those proteins, and also include a substantial number of transcripts that were affected by both nucleic acid-binding proteins; neither CsrA nor BadR affected each other’s transcript levels; CsrA can alter levels of transcripts such as *ospC* and *dbpBA* without affecting *rpoS*; transcription from the promoters of *ospC* and *dbpBA* do not require RpoS, but can be transcribed by the housekeeping sigma, RpoD; and, under the conditions examined by these studies, *rpoS* was transcribed from only its RpoD-dependent promoters.

None of the regulons defined by the current studies are likely to be complete. Had we chosen another growth condition, those stimuli could affect regulatory networks to the extent that additional members of these regulons might have been detected, while other transcripts might have been obscured by the effects of competing factors. For example, variations in culture conditions, such as acid stress, temperature, or osmolarity, can have significant effects on cellular levels of RpoS, explaining the substantial differences previously found between different analyses of *rpoS* mutant *B*. *burgdorferi* [6, 8, 10, 91–96]. We note also that the majority of previous studies on borrelial RpoS function have focused on bacteria cultured under conditions that caused high-level RpoS expression. We opted not to replicate such analyses because the large differences in RpoS content between wild-type bacteria that express high levels of the protein and an *rpoS* mutant can lead one to overlook subtleties. For example, prior array-based studies reported that levels of *ospC* and *dbpBA* were found to be greatly diminished in *rpoS* mutants in some instances, indicating that RpoS plays a positive role in their expression, but the low levels of *ospC*, *dbpBA*, etc. in *rpoS* mutants led to assumptions that RpoS is essential for their transcription [5, 7]. However, under the studied culture conditions, *rpoS*, *ospC*, and *dbpBA* were produced at significantly detectable levels in all strains, and neither *ospC* nor *dbpBA* were affected by deletion of *rpoS*. This demonstrates that both *ospC* and *dbpBA* are transcribed by RpoD-directed RNA polymerase. The previously-reported effects of RpoS enhancing expression of *ospC* and *dbpBA* indicate that either their promoters can also be recognized by RpoS-containing holoenzyme, or RpoS controls production of one or more factors that affect *ospC* and *dbpBA* transcript levels (e.g. DNA-binding proteins that stimulate transcription) [5, 97]. Regarding the first hypothesis, while elevated RpoS content can correlate with increased transcription of *ospC*, there is no strong evidence that RpoS-RNA polymerase holoenzyme directly transcribes the *ospC* promoter. Studies have been performed on the *ospC* promoter in the unrelated bacterium *E*. *coli*, but those studies determined that the two species’ RNA polymerases recognize different DNA sequences, so firm conclusions cannot be drawn from that report [97]. In support of the latter hypothesis, DNA sequences adjacent to the *ospC* promoter are required for maximal transcription [98–101], and a recent study provided evidence for at least one RpoS-controlled DNA-binding protein [73]. In addition, this can explain how *ospC* is repressed early during mammalian infection, while *dbpBA* and *rpoS* continue to be expressed [59, 102, 103]. Clearly, much remains to be learned about the mechanisms by which *B*. *burgdorferi* controls transcription of *ospC* and other virulence factors.

In addition, only a portion of the *badR*-affected transcripts observed in the current RNA-Seq study were also observed to be affected by *badR* in a previous, array-based study[16]. As a caveat, arrays measure only transcripts that hybridize with a probe derived from a segment of each gene, and hybridization efficiency is sensitive to temperature, pH, salt concentrations, and other experimental conditions. In addition, all prior array-based analyses studied only mRNAs, without considering intergenic or antisense ncRNAs. Even with those caveats, the data suggest that some of the transcripts affected by *badR* in the current study were influenced by additional regulatory factors that had little-to-no effect on them under the conditions of the previous transcriptome analysis. These might include other regulatory proteins, or, since BadR function is dependent upon cellular carbohydrate contents, differences in metabolic status or nutrient composition between batches of culture media might have contributed to results. These variations reinforce the hypothesis that *B*. *burgdorferi* uses multiple factors in a complex network of overlapping regulons, such that fluctuations in levels of a single regulatory factor may have significant impacts on some targets but not on others. Indeed, all intensively studied operons of *B*. *burgdorferi* are controlled by multiple factors. For example, transcription of the *erp* operons is directly regulated by the BpaB repressor, BpuR co-repressor, and EbfC antirepressor proteins, each of which also regulates other transcripts in various ways [104, 105]. Data from the current study further aids dissection of the regulatory interplay of *B*. *burgdorferi*.

Prior studies detected two promoters that drive transcription of *rpoS*, one of which is dependent upon RpoN, and the other upon RpoD [11, 15, 16, 89] (Fig 10). The current study demonstrates that transcription initiating from the RpoD-dependent promoter(s) 5’ of the upstream *flgI* and *flgJ* ORFs [90] probably also contributes to the expression of *rpoS* (Fig 10). The previously-identified RpoD-dependent promoter of *rpoS* lies within the *flgJ* ORF [11], so it is unlikely that a feature at the end of *flgJ* could terminate transcription that arose from one RpoD-dependent promoter but not the other. Further studies are required to determine whether the upstream promoters are regulated by *B*. *burgdorferi*, and how use of each promoter affects the others. It is also notable that all *rpoS* transcripts in the *rpoN* mutant originated from the RpoD-dependent promoters, serving as a reminder that the RpoD promoters must always be considered when studying conditions and regulatory factors that affect borrelial RpoS levels. These analyses also indicated considerable diminishment of transcription between *flgJ* and *rpoS*, suggestive of a regulatory mechanism operating in that area. The sequence does not contain an obvious intrinsic terminator [30]. Several proteins are known to bind DNA in this region, including BadR [15, 17]. While prior research on those factors has focused on the RpoN-dependent promoter, it would be worthwhile to examine their effects on transcription from the RpoD-dependent promoters.

In conclusion, these studies expanded knowledge of the *B*. *burgdorferi* CsrA, BadR, RpoS, and RpoN regulons. Under the examined growth conditions, none of these regulatory proteins were observed to have impacts on any of the other three, indicating that effects of two proteins on a single transcript were due to converging regulatory pathways. This lack of impact on one another was true when considering both adjusted and non-adjusted p-values. Substantial convergence was observed between the *csrA* and *badR* mutant transcriptomes, as well as evidence that each regulates a distinct set of transcripts. CsrA exerted significant impacts upon numerous transcripts, such as *ospC* and *dbpBA*, through mechanisms that appear to be independent of RpoS, further advancing understanding of these infection-associated regulons.

## Supporting information

S1 Fig Principal component analysis of RNA-Seq samples. Principle component analysis was performed for all 19 samples examined in this study and the results are plotted above. “WT-1” and “WT-2” indicate data from the two sets of wild-type cultures.
(PDF)

S1 Table Primers used in these studies. Contains all primers used within these studies for qPCR and qRT-PCR. Name and nucleotide sequence (5’-3’) is given for each.
(DOCX)

S2 Table Differentially expressed transcripts when comparing the *csrA* mutant to wild-type, listed in order of fold change. The included transcripts met the criteria of >1 log2 fold-change and an adjusted p-value (padj) when comparing the *csrA* mutant to wild-type sorted by fold change. A total of 239 transcripts were differentially regulated, not including the mutated gene, by the mutation. The first column contains the CDS/custom transcript ID which is the transcript ID for all coding sequences obtained from the NCBI Gene file format file or the transcript ID given to ncRNAs. RefSeq entries are further separated by the character “_”. The first portion gives the genetic element from which it is derived, the second describes the type of element (CDS), the third provides RefSeq ID, and the fourth provides a number indicating the particular entries ordered number in the RefSeq entry. The second column is the gene information, for the ncRNAs it contains the location relative to other genes and for predicted or known genes it contains gene name. The remaining columns describe the various metrics of expression of each impacted transcript including, base mean (average library size normalized counts across all samples), log2FC (Fold change estimate), lfcSE (uncertainty of the log fold change estimate), stat (Wald statistic), pvalue, padj (pvalue following Benjamini-Hochberg adjustment). ORFs and ncRNAs are identified according to the names or numbers assigned to genes and transcripts by the initial genome sequencing of *B*. *burgdorferi* strain B31 [18, 31] or from our previous analyses of that strain’s ncRNA transcriptome [30].
(DOCX)

S3 Table Differentially expressed transcripts when comparing the *badR* mutant to wild-type, listed in order of fold change. The included transcripts met the criteria of >1 log2 fold-change and an adjusted p-value (padj) when comparing the *badR* mutant to wild-type sorted by fold change. A total of 234 transcripts were differentially regulated, not including the mutated gene, by the mutation. The first column contains the CDS/custom transcript ID which is the transcript ID for all coding sequences obtained from the NCBI Gene file format file or the transcript ID given to ncRNAs. RefSeq entries are further separated by the character “_”. The first portion gives the genetic element from which it is derived, the second describes the type of element (CDS), the third provides RefSeq ID, and the fourth provides a number indicating the particular entries ordered number in the RefSeq entry. The second column is the gene information, for the ncRNAs it contains the location relative to other genes and for predicted or known genes it contains gene name. The remaining columns describe the various metrics of expression of each impacted transcript including, base mean (average library size normalized counts across all samples), log2FC (Fold change estimate), lfcSE (uncertainty of the log fold change estimate), stat (Wald statistic), pvalue, padj (pvalue following Benjamini-Hochberg adjustment). ORFs and ncRNAs are identified according to the names or numbers assigned to genes and transcripts by the initial genome sequencing of *B*. *burgdorferi* strain B31 [18, 31] or from our previous analyses of that strain’s ncRNA transcriptome [30].
(DOCX)

S4 Table Intersection table of all transcripts that differentially expressed across all mutants. Contains the entire set of transcripts that were differentially expressed under any condition and what condition they were impacted by. A total of 331 transcripts, including those mutated, were differentially expressed across our total data set. The first column is the gene information, for the ncRNAs it contains the location relative to other genes and for predicted or known genes it contains gene name. The second column contains the CDS/custom transcript ID which is the transcript ID for all coding sequences obtained from the NCBI Gene file format file or the transcript ID given to ncRNAs. RefSeq entries are further separated by the character “_”. The first portion gives the genetic element from which it is derived, the second describes the type of element (CDS), the third provides RefSeq ID, and the fourth provides a number indicating the particular entries ordered number in the RefSeq entry. The following 8 columns are given as each mutant and increased abundance or decreased abundance. If a transcript was differentially expressed in a condition it’s cell value is given as TRUE. Empty cells indicate that a transcript was not impacted by a given condition. ORFs and ncRNAs are listed according to the numerical order assigned to genes and replicons by the initial genome sequencing of *B*. *burgdorferi* strain B31 (13, 69).
(XLSX)

S5 Table Total differential expression testing table for the *csrA* mutant. Contains the differential expression testing results for the *csrA* mutant compared to wild-type. The first column is the gene information, for the ncRNAs it contains the location relative to other genes and for predicted or known genes it contains gene name. The second column contains the CDS/custom transcript ID which is the transcript ID for all coding sequences obtained from the NCBI Gene file format file or the transcript ID given to ncRNAs. RefSeq entries are further separated by the character “_”. The first portion gives the genetic element from which it is derived, the second describes the type of element (CDS), the third provides RefSeq ID, and the fourth provides a number indicating the particular entries ordered number in the RefSeq entry. The remaining columns describe the various metrics of expression of each impacted transcript including, base mean (average library size normalized counts across all samples), log2FC (Fold change estimate), lfcSE (uncertainty of the log fold change estimate), stat (Wald statistic), pvalue, padj (pvalue following Benjamini-Hochberg adjustment). ORFs and ncRNAs are listed according to the numerical order assigned to genes and replicons by the initial genome sequencing of *B*. *burgdorferi* strain B31 (13, 69).
(XLSX)

S6 Table Total differential expression testing table for the *badR* mutant. Contains the differential expression testing results for the *badR* mutant compared to wild-type. The first column is the gene information, for the ncRNAs it contains the location relative to other genes and for predicted or known genes it contains gene name. The second column contains the CDS/custom transcript ID which is the transcript ID for all coding sequences obtained from the NCBI Gene file format file or the transcript ID given to ncRNAs. RefSeq entries are further separated by the character “_”. The first portion gives the genetic element from which it is derived, the second describes the type of element (CDS), the third provides RefSeq ID, and the fourth provides a number indicating the particular entries ordered number in the RefSeq entry. The remaining columns describe the various metrics of expression of each impacted transcript including, base mean (average library size normalized counts across all samples), log2FC (Fold change estimate), lfcSE (uncertainty of the log fold change estimate), stat (Wald statistic), pvalue, padj (pvalue following Benjamini-Hochberg adjustment). ORFs and ncRNAs are listed according to the numerical order assigned to genes and replicons by the initial genome sequencing of *B*. *burgdorferi* strain B31 (13, 69).
(XLSX)

S7 Table Total differential expression testing table for the *rpoS* mutant. Contains the differential expression testing results for the *rpoS* mutant compared to wild-type. The first column is the gene information, for the ncRNAs it contains the location relative to other genes and for predicted or known genes it contains gene name. The second column contains the CDS/custom transcript ID which is the transcript ID for all coding sequences obtained from the NCBI Gene file format file or the transcript ID given to ncRNAs. RefSeq entries are further separated by the character “_”. The first portion gives the genetic element from which it is derived, the second describes the type of element (CDS), the third provides RefSeq ID, and the fourth provides a number indicating the particular entries ordered number in the RefSeq entry. The remaining columns describe the various metrics of expression of each impacted transcript including, base mean (average library size normalized counts across all samples), log2FC (Fold change estimate), lfcSE (uncertainty of the log fold change estimate), stat (Wald statistic), pvalue, padj (pvalue following Benjamini-Hochberg adjustment). ORFs and ncRNAs are listed according to the numerical order assigned to genes and replicons by the initial genome sequencing of *B*. *burgdorferi* strain B31 (13, 69).
(XLSX)

S8 Table Total differential expression testing table for the *rpoN*mutant. Contains the differential expression testing results for the *rpoN* mutant compared to wild-type. The first column is the gene information, for the ncRNAs it contains the location relative to other genes and for predicted or known genes it contains gene name. The second column contains the CDS/custom transcript ID which is the transcript ID for all coding sequences obtained from the NCBI Gene file format file or the transcript ID given to ncRNAs. RefSeq entries are further separated by the character “_”. The first portion gives the genetic element from which it is derived, the second describes the type of element (CDS), the third provides RefSeq ID, and the fourth provides a number indicating the particular entries ordered number in the RefSeq entry. The remaining columns describe the various metrics of expression of each impacted transcript including, base mean (average library size normalized counts across all samples), log2FC (Fold change estimate), lfcSE (uncertainty of the log fold change estimate), stat (Wald statistic), pvalue, padj (pvalue following Benjamini-Hochberg adjustment). ORFs and ncRNAs are listed according to the numerical order assigned to genes and replicons by the initial genome sequencing of *B*. *burgdorferi* strain B31 (13, 69).
(XLSX)
