# Supplementary material for: Transcriptomic insights on the virulence-controlling CsrA, BadR, RpoN, and RpoS regulatory networks in the Lyme disease spirochete
Source: PLoS One. 2018 Aug 30;13(8):e0203286. doi: 10.1371/journal.pone.0203286 (PMC6117026; doi:10.1371/journal.pone.0203286)
Supplement: S1 Fig — Principle component analysis was performed for all 19 samples examined in this study and the results are plotted above. “WT-1” and “WT-2” indicate data from the two sets of wild-type cultures. (PDF) [file pone.0203286.s001.pdf]

Figure S1: Principle component plot of RNA-Seq samples

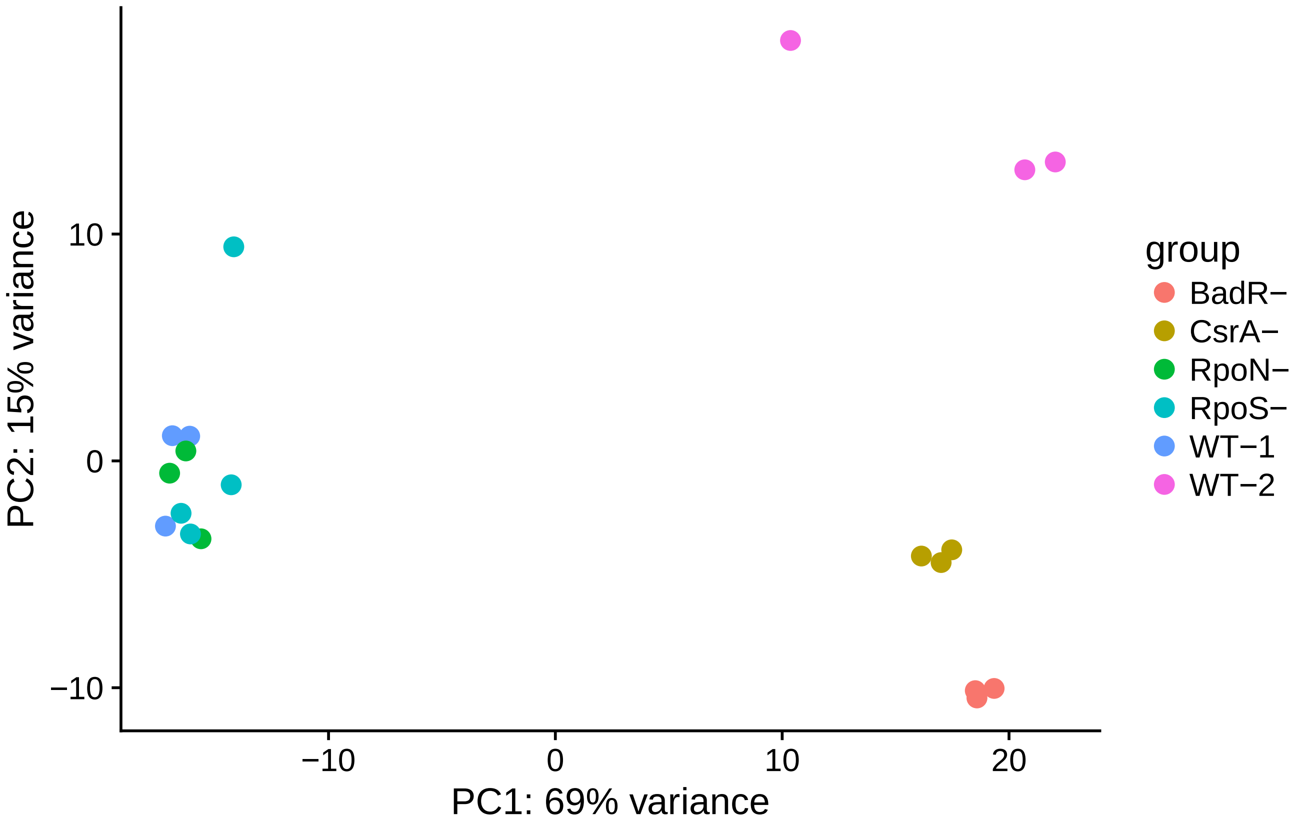

Principle component analysis was performed for all 19 samples examined in this study and the results are plotted above.
