# Supplementary material for: Transcriptomic insights on the virulence-controlling CsrA, BadR, RpoN, and RpoS regulatory networks in the Lyme disease spirochete
Source: PLoS One. 2018 Aug 30;13(8):e0203286. doi: 10.1371/journal.pone.0203286 (PMC6117026; doi:10.1371/journal.pone.0203286)
Supplement: S1 Table — Contains all primers used within these studies for qPCR and qRT-PCR. Name and nucleotide sequence (5’-3’) is given for each. (DOCX) [file pone.0203286.s002.docx]

| **Table S1: Primers used in these studies** | |
| --- | --- |
| **Primer Name** | **Sequence (5' -> 3')** |
| *ospC* F | CTTGCTGTGAAAGAGGTTGAAG |
| *ospC* R | CTCCCGCTAACAATGATCCA |
| *dbpA* F | GCTGCTCTTAAGGGCGTAAA |
| *dbpA* R | CTACTGTAGTAGCTCGCACTTT |
| *glpF* F | CAGGGAAAGTTGCCATTAT |
| *glpF* R | CAGGGAAAGTTGCCATTAT |
| *glpK* F | TTTATTGGAGGACCTGTAATC |
| *glpK* R | TGTTGAGCCTCTTGTTATTC |
| *glpD F* | GGCATTGCCCTTAACTATAC |
| *glpD* R | CTACCTTGGGAAGGTTTAATG |
| *spoVG* F | GCGATGCCTAACAGAAGAACTA |
| *spoVG* R | AGTTCAAGATCGGCTGGATTT |
| *bbk32* F | TTCCCTTAGCGGTGAAAGTG |
| *bbk32* R | GTAAGGAACTCTTTGGCCTTAAATC |
| *sodA* F | TGTCCTGAGAGTGGCCTTA |
| *sodA* R | GCATGCTCCCAAACATCAATAC |
| *bpuR* F | GGCTCTTCTGCAAGGCATAAT |
| *bpuR* R | GCCCGCCTGATAAATGAGATT |
| *bosR* F | TGCAATGCCCTGAGTAAATGA |
| *bosR* R | TGCAATCAAGTCCACCCTATTC |
| lp28-4 F | GCGTATAGTTCGTTGGCTGTA |
| lp28-4 R | GCAGTGGGTCTAGGCATATTAC |
| *dnaA* F | CCAAFTCCAACTCCACCATAAA |
| *dnaA* R | GGGCCAAATAATAAACTTGCTTACA |
| *cdaA F* | CTCTTCACGATGGAGCTGTAAT |
| *cdaA* R | GTCCTGCTCTATGTCTTGTTCC |
