# Supplementary material for: Transcriptomic insights on the virulence-controlling CsrA, BadR, RpoN, and RpoS regulatory networks in the Lyme disease spirochete
Source: PLoS One. 2018 Aug 30;13(8):e0203286. doi: 10.1371/journal.pone.0203286 (PMC6117026; doi:10.1371/journal.pone.0203286)
Supplement: S2 Table — The included transcripts met the criteria of >1 log2 fold-change and an adjusted p-value (padj) when comparing the csrA mutant to wild-type sorted by fold change. A total of 239 transcripts were differentially regulated, not including the mutated gene, by the mutation. The first column contains the CDS/custom transcript ID which is the transcript ID for all coding sequences obtained from the NCBI Gene file format file or the transcript ID given to ncRNAs. RefSeq entries are further separated by the character “_”. The first portion gives the genetic element from which it is derived, the second describes the type of element (CDS), the third provides RefSeq ID, and the fourth provides a number indicating the particular entries ordered number in the RefSeq entry. The second column is the gene information, for the ncRNAs it contains the location relative to other genes and for predicted or known genes it contains gene name. The remaining columns describe the various metrics of expression of each impacted transcript including, base mean (average library size normalized counts across all samples), log2FC (Fold change estimate), lfcSE (uncertainty of the log fold change estimate), stat (Wald statistic), pvalue, padj (pvalue following Benjamini-Hochberg adjustment). ORFs and ncRNAs are identified according to the names or numbers assigned to genes and transcripts by the initial genome sequencing of B. burgdorferi strain B31 [18, 31] or from our previous analyses of that strain’s ncRNA transcriptome [30]. (DOCX) [file pone.0203286.s003.docx]

**Table S3: Differentially expressed transcripts when comparing ∆csrA mutant to wild-type, listed in order of fold change.** The included transcripts met the criteria of >1 log2 fold-change and an adjusted p-value (padj) when comparing the ∆*csrA* mutant to wild-type sorted by fold change. A total of 239 transcripts were differentially regulated, not including the mutated gene, by the mutation. The first column contains the CDS/custom transcript ID which is the transcript ID for all coding sequences obtained from the NCBI Gene file format file or the transcript ID given to ncRNAs. RefSeq entries are further separated by the character “_”. The first portion gives the genetic element from which it is derived, the second describes the type of element (CDS), the third provides RefSeq ID, and the fourth provides a number indicating the particular entries ordered number in the RefSeq entry. The second column is the gene information, for the ncRNAs it contains the location relative to other genes and for predicted or known genes it contains gene name. The remaining columns describe the various metrics of expression of each impacted transcript including, base mean (average library size normalized counts across all samples), log2FC (Fold change estimate), lfcSE (uncertainty of the log fold change estimate), stat (Wald statistic), pvalue, padj (pvalue following Benjamini-Hochberg adjustment). ORFs and ncRNAs are identified according to the names or numbers assigned to genes and transcripts by the initial genome sequencing of *B. burgdorferi* strain B31 [18, 31] or from our previous analyses of that strain’s ncRNA transcriptome [30].

| **RefSeq CDS/Custom Transcript ID** | **Gene Name** | **baseMean** | **log2FoldChange** | **lfcSE** | **stat** | **pvalue** | **padj** |
| --- | --- | --- | --- | --- | --- | --- | --- |
| ncRNA0072 | A-(BB_0454) | 396.651084 | 2.956000331 | 0.659455311 | 4.482487719 | 7.38E-06 | 8.16E-05 |
| lcl\|NC_000956.1_cds_NP_051504.1_1374 | bdrV | 634.196218 | 2.699534338 | 0.317216535 | 8.510068181 | 1.74E-17 | 1.18E-15 |
| lcl\|NC_001903.1_cds_NP_046992.2_803 | chbB | 1687.198719 | 2.399229955 | 0.263942445 | 9.089973967 | 9.91E-20 | 8.88E-18 |
| lcl\|NC_001857.2_cds_NP_045747.1_1339 | osm28 | 12273.60539 | 2.234165789 | 0.394208008 | 5.667479461 | 1.45E-08 | 3.16E-07 |
| ncRNA0152 | IA-(BB_P32/BB_P33,BB_P33) | 276.3515173 | 2.167303442 | 0.364938587 | 5.938816884 | 2.87E-09 | 7.64E-08 |
| lcl\|NC_001857.2_cds_NP_045676.1_1287 | BB_A03 | 12443.29488 | 2.136543909 | 0.336492459 | 6.349455542 | 2.16E-10 | 6.46E-09 |
| lcl\|NC_000948.1_cds_NP_051194.2_853 | BB_P33 | 507.5530008 | 2.135405263 | 0.245441907 | 8.700247189 | 3.31E-18 | 2.45E-16 |
| lcl\|NC_000956.1_cds_NP_051521.1_1385 | BB_Q62 | 20.48709362 | 2.091457406 | 0.544783424 | 3.839062122 | 0.000123505 | 0.000902701 |
| ncRNA0218 | IA-(BB_N32/BB_N33,BB_N33) | 29.8614237 | 2.060586205 | 0.516857045 | 3.986762348 | 6.70E-05 | 0.000525662 |
| ncRNA0311 | A-(BB_A04) | 361.981407 | 1.92915261 | 0.411397747 | 4.689263914 | 2.74E-06 | 3.36E-05 |
| lcl\|NC_000948.1_cds_NP_051193.1_852 | BB_P32 | 812.8429087 | 1.909156521 | 0.235635362 | 8.102164722 | 5.40E-16 | 3.06E-14 |
| lcl\|NC_001857.2_cds_NP_045727.1_1325 | BB_A54 | 599.9624458 | 1.900543585 | 0.367018587 | 5.178330612 | 2.24E-07 | 3.60E-06 |
| lcl\|NC_000954.1_cds_NP_051445.1_1089 | BB_N33 | 157.3216497 | 1.888712116 | 0.288264846 | 6.552002924 | 5.68E-11 | 2.10E-09 |
| lcl\|NC_001318.1_cds_NP_212499.1_346 | la7 | 8401.745644 | 1.813485579 | 0.28233191 | 6.423239871 | 1.33E-10 | 4.09E-09 |
| lcl\|NC_001856.1_cds_NP_045633.1_1254 | BB_J09 | 24951.96368 | 1.809636522 | 0.37729147 | 4.796388644 | 1.62E-06 | 2.07E-05 |
| lcl\|NC_001903.1_cds_NP_046991.1_802 | chbA | 2551.066468 | 1.802467823 | 0.276127156 | 6.527673154 | 6.68E-11 | 2.42E-09 |
| lcl\|NC_001318.1_cds_NP_212498.1_345 | mgsA | 3019.346339 | 1.742561091 | 0.164753604 | 10.57677071 | 3.82E-26 | 5.42E-24 |
| lcl\|NC_000954.1_cds_NP_051446.1_1090 | bdrQ | 227.796319 | 1.701845715 | 0.263258846 | 6.464533837 | 1.02E-10 | 3.46E-09 |
| lcl\|NC_000954.1_cds_NP_051443.1_1087 | BB_N31 | 161.0855567 | 1.627410115 | 0.348823424 | 4.665426693 | 3.08E-06 | 3.69E-05 |
| lcl\|NC_000954.1_cds_NP_051444.1_1088 | BB_N32 | 241.9969265 | 1.586427412 | 0.308950619 | 5.134889897 | 2.82E-07 | 4.41E-06 |
| lcl\|NC_001857.2_cds_NP_045704.1_1306 | BB_A31 | 693.6564221 | 1.565907435 | 0.26914935 | 5.817987062 | 5.96E-09 | 1.42E-07 |
| ncRNA0186 | AIA-(BB_O32,BB_O32/BB_O33,BB_O33) | 90.75800452 | 1.563150832 | 0.325703169 | 4.799311092 | 1.59E-06 | 2.06E-05 |
| ncRNA0185 | I-(BB_O29/BB_O30) | 1573.01516 | 1.543988845 | 0.477729846 | 3.231928796 | 0.001229577 | 0.006403576 |
| lcl\|NC_001903.1_cds_NP_046990.2_801 | chbC | 11469.16755 | 1.518292428 | 0.224234778 | 6.77099442 | 1.28E-11 | 5.58E-10 |
| ncRNA0259 | AA-(BB_G07,BB_G08) | 88.149747 | 1.50709705 | 0.260690153 | 5.781181338 | 7.42E-09 | 1.71E-07 |
| lcl\|NC_001318.1_cds_NP_212542.1_389 | fruA1 | 6219.62582 | 1.501253133 | 0.222065333 | 6.760411964 | 1.38E-11 | 5.86E-10 |
| lcl\|NC_000956.1_cds_NP_051489.1_1361 | BB_Q27 | 184.692011 | 1.478250023 | 0.447598087 | 3.302628105 | 0.000957833 | 0.005142466 |
| lcl\|NC_000956.1_cds_NP_051502.1_1373 | BB_Q40 | 481.1703282 | 1.463624848 | 0.367880474 | 3.978533659 | 6.93E-05 | 0.000541691 |
| lcl\|NC_000954.1_cds_NP_051450.1_1094 | erpQ | 1968.305688 | 1.458291354 | 0.359888349 | 4.052066028 | 5.08E-05 | 0.000415658 |
| lcl\|NC_000948.1_cds_NP_051192.1_851 | BB_P31 | 319.4277675 | 1.450607612 | 0.331481902 | 4.37612915 | 1.21E-05 | 0.000123284 |
| lcl\|NC_001903.1_cds_NP_047015.1_821 | BB_B29 | 24118.646 | 1.407250063 | 0.212691935 | 6.616377174 | 3.68E-11 | 1.42E-09 |
| ncRNA0326 | I-(BB_A66/BB_A68) | 456.2996811 | 1.397904593 | 0.367271858 | 3.806184882 | 0.000141127 | 0.001014089 |
| lcl\|NC_001903.1_cds_NP_046987.2_798 | BB_B01 | 877.5381411 | 1.388320735 | 0.215561895 | 6.440473802 | 1.19E-10 | 3.76E-09 |
| lcl\|NC_001903.1_cds_NP_046993.1_804 | BB_B07 | 8409.809781 | 1.379859483 | 0.163157096 | 8.457244702 | 2.74E-17 | 1.79E-15 |
| lcl\|NC_001852.1_cds_NP_045464.1_1159 | BB_G02 | 182.6516298 | 1.367896406 | 0.397186284 | 3.443966874 | 0.000573246 | 0.00334328 |
| lcl\|NC_001318.1_cds_NP_212901.1_720 | murG | 901.267597 | 1.366187686 | 0.281129643 | 4.859635828 | 1.18E-06 | 1.62E-05 |
| lcl\|NC_000949.1_cds_NP_051240.1_894 | bdrE | 326.736536 | 1.358376963 | 0.242081995 | 5.611226741 | 2.01E-08 | 4.28E-07 |
| lcl\|NC_001857.2_cds_NP_045703.1_1305 | BB_A30 | 869.6149934 | 1.35254358 | 0.216997662 | 6.23298689 | 4.58E-10 | 1.34E-08 |
| lcl\|NC_001318.1_cds_NP_212319.1_174 | BB_0185 | 1345.323777 | 1.349770078 | 0.218738922 | 6.17069 | 6.80E-10 | 1.93E-08 |
| lcl\|NC_001857.2_cds_NP_045726.1_1324 | BB_A53 | 375.6912082 | 1.346830886 | 0.375697753 | 3.584878732 | 0.000337235 | 0.002183692 |
| lcl\|NC_000948.1_cds_NP_051195.1_854 | bdrA | 840.2082301 | 1.327617956 | 0.302692249 | 4.386032214 | 1.15E-05 | 0.000119145 |
| lcl\|NC_000949.1_cds_NP_051238.1_893 | BB_S35 | 265.7310316 | 1.325298003 | 0.239761792 | 5.52756131 | 3.25E-08 | 6.66E-07 |
| lcl\|NC_001318.1_cds_NP_212904.1_723 | BB_0770 | 1364.054451 | 1.318173253 | 0.24452395 | 5.390773603 | 7.02E-08 | 1.32E-06 |
| ncRNA0073 | I-(BB_t06/BB_0461) | 2784.933991 | 1.305078491 | 0.230041096 | 5.673240628 | 1.40E-08 | 3.10E-07 |
| lcl\|NC_001857.2_cds_NP_045731.1_1327 | BB_A58 | 8350.064089 | 1.298241485 | 0.296675316 | 4.375967316 | 1.21E-05 | 0.000123284 |
| lcl\|NC_001318.1_cds_NP_212907.1_727 | BB_0773 | 467.79866 | 1.273917781 | 0.178834561 | 7.123442872 | 1.05E-12 | 4.84E-11 |
| lcl\|NC_001903.1_cds_NP_047014.1_820 | BB_B28 | 4249.142152 | 1.266728604 | 0.143683797 | 8.816085248 | 1.19E-18 | 9.18E-17 |
| lcl\|NC_001318.1_cds_NP_212919.1_739 | spoVG | 2664.703766 | 1.261401239 | 0.211981895 | 5.950514032 | 2.67E-09 | 7.23E-08 |
| lcl\|NC_001318.1_cds_NP_212773.1_599 | potD | 1482.689456 | 1.254211323 | 0.286927391 | 4.371180168 | 1.24E-05 | 0.000125269 |
| lcl\|NC_001318.1_cds_NP_212903.1_722 | BB_0769 | 1653.77636 | 1.253523799 | 0.240916322 | 5.203150152 | 1.96E-07 | 3.18E-06 |
| lcl\|NC_001855.1_cds_NP_045618.1_1246 | BB_K47 | 2505.671645 | 1.249316306 | 0.317102255 | 3.939790037 | 8.16E-05 | 0.000631294 |
| lcl\|NC_001318.1_cds_NP_212671.1_500 | BB_0537 | 1848.252264 | 1.232384605 | 0.238276054 | 5.172087516 | 2.31E-07 | 3.65E-06 |
| lcl\|NC_001318.1_cds_NP_212696.1_524 | BB_0562 | 1222.608908 | 1.195586657 | 0.147028901 | 8.13164381 | 4.24E-16 | 2.49E-14 |
| lcl\|NC_001903.1_cds_NP_047009.2_815 | BB_B23 | 2583.013976 | 1.194275661 | 0.153920788 | 7.759027729 | 8.56E-15 | 4.29E-13 |
| lcl\|NC_001318.1_cds_NP_212812.1_638 | BB_0678 | 5061.700522 | 1.19084516 | 0.184625608 | 6.450054089 | 1.12E-10 | 3.59E-09 |
| lcl\|NC_001318.1_cds_NP_212541.1_388 | manA | 2887.103188 | 1.179754844 | 0.149300098 | 7.901902686 | 2.75E-15 | 1.42E-13 |
| lcl\|NC_001318.1_cds_NP_212652.1_488 | dnaK | 5264.983523 | 1.178215406 | 0.19904193 | 5.919433176 | 3.23E-09 | 8.21E-08 |
| lcl\|NC_001318.1_cds_NP_212976.2_794 | arcB | 2452.381364 | 1.161259482 | 0.247934672 | 4.6837317 | 2.82E-06 | 3.43E-05 |
| lcl\|NC_000950.1_cds_NP_051281.1_933 | BB_R34 | 206.0163455 | 1.161125002 | 0.218429448 | 5.315789667 | 1.06E-07 | 1.85E-06 |
| lcl\|NC_001318.1_cds_NP_212900.1_719 | cvpA | 420.5109223 | 1.157542839 | 0.239778975 | 4.827541022 | 1.38E-06 | 1.86E-05 |
| lcl\|NC_001318.1_cds_NP_212464.1_313 | BB_0330 | 9233.475103 | 1.149087729 | 0.219007371 | 5.246799329 | 1.55E-07 | 2.56E-06 |
| ncRNA0327 | I-(BB_A68/BB_A69) | 288.5694033 | 1.134754485 | 0.411545698 | 2.75729886 | 0.005828106 | 0.022764367 |
| lcl\|NC_001903.1_cds_NP_047004.2_812 | guaA | 11235.00282 | 1.122969516 | 0.136736824 | 8.212634214 | 2.16E-16 | 1.32E-14 |
| lcl\|NC_000949.1_cds_NP_051237.1_892 | BB_S34 | 208.7781932 | 1.122702036 | 0.372157025 | 3.016742832 | 0.002555065 | 0.011824118 |
| lcl\|NC_001318.1_cds_NP_212672.1_501 | BB_0538 | 967.7451027 | 1.121459917 | 0.239070239 | 4.690922314 | 2.72E-06 | 3.36E-05 |
| lcl\|NC_001318.1_cds_NP_212169.1_33 | BB_0035 | 2717.907721 | 1.110406753 | 0.214694041 | 5.172042722 | 2.32E-07 | 3.65E-06 |
| lcl\|NC_001318.1_cds_NP_212772.1_598 | BB_0638 | 3886.718713 | 1.105574398 | 0.209984884 | 5.265018978 | 1.40E-07 | 2.34E-06 |
| lcl\|NC_001318.1_cds_NP_212771.2_597 | BB_0637 | 8048.197727 | 1.095157563 | 0.261344496 | 4.190474945 | 2.78E-05 | 0.00025591 |
| lcl\|NC_001903.1_cds_NP_046988.1_799 | BB_B02 | 2128.099072 | 1.093811634 | 0.196510568 | 5.566172052 | 2.60E-08 | 5.47E-07 |
| lcl\|NC_001903.1_cds_NP_047013.1_819 | BB_B27 | 1716.86092 | 1.09085916 | 0.184033083 | 5.927516626 | 3.08E-09 | 7.94E-08 |
| lcl\|NC_001318.1_cds_NP_212813.2_639 | BB_0679 | 4281.290839 | 1.087255066 | 0.192459876 | 5.649255778 | 1.61E-08 | 3.47E-07 |
| lcl\|NC_001855.1_cds_NP_045620.1_1248 | BB_K49 | 1392.93963 | 1.08561374 | 0.354606502 | 3.061460336 | 0.002202602 | 0.010333418 |
| lcl\|NC_001318.1_cds_NP_212778.1_604 | nanE | 697.3884376 | 1.073611768 | 0.19917336 | 5.39033818 | 7.03E-08 | 1.32E-06 |
| lcl\|NC_000950.1_cds_NP_051280.1_932 | BB_R33 | 295.7891493 | 1.070632049 | 0.254705484 | 4.203411853 | 2.63E-05 | 0.000246019 |
| lcl\|NC_001318.1_cds_NP_212585.1_426 | BB_0451 | 435.9912764 | 1.065285948 | 0.208990807 | 5.097286147 | 3.45E-07 | 5.33E-06 |
| lcl\|NC_001318.1_cds_NP_212468.1_317 | BB_0334 | 3335.870455 | 1.049316796 | 0.157717762 | 6.653130124 | 2.87E-11 | 1.14E-09 |
| ncRNA0306 | I-(BB_J37/BB_J41) | 3515.789368 | 1.039600627 | 0.355673147 | 2.922910082 | 0.003467766 | 0.014950902 |
| ncRNA0307 | IA-(BB_J37/BB_J41,BB_J41) | 1187.105717 | 1.028436646 | 0.375322809 | 2.740138948 | 0.006141322 | 0.023823851 |
| lcl\|NC_000952.1_cds_NP_051365.1_1013 | BB_O32 | 381.8142121 | 1.026021902 | 0.245024295 | 4.187429255 | 2.82E-05 | 0.00025591 |
| lcl\|NC_001857.2_cds_NP_045725.1_1323 | BB_A52 | 1613.061442 | 1.01788004 | 0.312733352 | 3.254785695 | 0.00113478 | 0.005964598 |
| lcl\|NC_001318.1_cds_NP_212751.1_578 | BB_0617 | 777.7214187 | 1.015625323 | 0.226394111 | 4.486094267 | 7.25E-06 | 8.07E-05 |
| lcl\|NC_001318.1_cds_NP_212902.1_721 | BB_0768 | 1156.42566 | 1.01241342 | 0.234590479 | 4.315662868 | 1.59E-05 | 0.000156641 |
| lcl\|NC_001318.1_cds_NP_212975.1_793 | arcA | 2690.756525 | 1.011615231 | 0.157525181 | 6.421927127 | 1.35E-10 | 4.09E-09 |
| lcl\|NC_000952.1_cds_NP_051366.1_1014 | BB_O33 | 262.1173124 | 1.010347941 | 0.210338316 | 4.803442187 | 1.56E-06 | 2.04E-05 |
| lcl\|NC_001318.1_cds_NP_212620.2_459 | rpmC | 492.1829109 | 1.007744941 | 0.202337379 | 4.980517913 | 6.34E-07 | 9.31E-06 |
| lcl\|NC_001857.2_cds_NP_045733.1_1329 | BB_A60 | 1031.73146 | 1.00641711 | 0.222117345 | 4.531015402 | 5.87E-06 | 6.58E-05 |
| lcl\|NC_001318.1_cds_NP_212138.2_3 | BB_0004 | 3213.612708 | -1.011843497 | 0.350191279 | -2.889402332 | 0.003859749 | 0.016310552 |
| ncRNA0133 | A-(BB_B03) | 471.6283693 | -1.017684262 | 0.245633747 | -4.143096275 | 3.43E-05 | 0.000299246 |
| lcl\|NC_000950.1_cds_NP_051274.2_927 | bdrH | 752.05999 | -1.019325728 | 0.238699916 | -4.270322938 | 1.95E-05 | 0.000186747 |
| lcl\|NC_001318.1_cds_YP_008686594.1_748 | BB_0794 | 8844.212411 | -1.020839036 | 0.17423885 | -5.858848551 | 4.66E-09 | 1.15E-07 |
| lcl\|NC_001318.1_cds_NP_212568.1_409 | BB_0434 | 410.6547229 | -1.026236311 | 0.305759261 | -3.356353976 | 0.000789774 | 0.004409788 |
| lcl\|NC_001856.1_cds_NP_045650.1_1266 | BB_J26 | 38.55968829 | -1.054612052 | 0.379750298 | -2.777119751 | 0.005484297 | 0.021850826 |
| ncRNA0148 | A-(BB_P21) | 93.09625772 | -1.057974939 | 0.420039394 | -2.518751701 | 0.011777167 | 0.040193419 |
| ncRNA0035 | A-(BB_0208) | 510.9105757 | -1.073378765 | 0.291108995 | -3.687205759 | 0.00022673 | 0.001526171 |
| lcl\|NC_001318.1_cds_NP_212156.1_20 | ruvB | 2074.85845 | -1.076565311 | 0.202076814 | -5.327505365 | 9.96E-08 | 1.75E-06 |
| lcl\|NC_001855.1_cds_NP_045596.1_1233 | BB_K22 | 260.1823499 | -1.082818368 | 0.34636474 | -3.126237301 | 0.001770586 | 0.008639852 |
| ncRNA0232 | I-(BB_D22/BB_D23) | 141.5006657 | -1.092538093 | 0.252136531 | -4.333120985 | 1.47E-05 | 0.000146408 |
| lcl\|NC_001849.2_cds_NP_045388.1_1111 | BB_D04 | 31.08243243 | -1.094553278 | 0.428260926 | -2.555809345 | 0.010594115 | 0.037122999 |
| lcl\|NC_000956.1_cds_NP_051533.1_1388 | BB_Q85 | 31.08243243 | -1.094553278 | 0.428260926 | -2.555809345 | 0.010594115 | 0.037122999 |
| lcl\|NC_000952.1_cds_NP_051362.1_1010 | BB_O29 | 207.6325904 | -1.106695965 | 0.441931172 | -2.50422698 | 0.012271928 | 0.041402801 |
| lcl\|NC_000950.1_cds_NP_051278.1_930 | BB_R31 | 1579.32556 | -1.113009695 | 0.443119222 | -2.51176126 | 0.012013033 | 0.040672355 |
| lcl\|NC_001850.1_cds_NP_045416.1_1133 | BB_E09 | 524.595921 | -1.11887635 | 0.396427462 | -2.82239869 | 0.004766588 | 0.019798779 |
| lcl\|NC_001850.1_cds_NP_045428.1_1139 | BB_E21 | 8358.69302 | -1.131514218 | 0.253371318 | -4.465833882 | 7.98E-06 | 8.76E-05 |
| lcl\|NC_001318.1_cds_YP_008686588.1_680 | cabP | 3690.875669 | -1.146485517 | 0.267498556 | -4.285950297 | 1.82E-05 | 0.000176067 |
| lcl\|NC_001318.1_cds_NP_212419.2_270 | BB_0285 | 4139.079468 | -1.154948662 | 0.317610837 | -3.636364149 | 0.000276513 | 0.001839462 |
| lcl\|NC_001855.1_cds_NP_045607.1_1239 | BB_K34 | 221.9972259 | -1.173040964 | 0.21313803 | -5.503668033 | 3.72E-08 | 7.45E-07 |
| ncRNA0007 | A-(BB_0014) | 214.8526993 | -1.215649307 | 0.481870969 | -2.522769338 | 0.011643475 | 0.040193419 |
| lcl\|NC_001318.1_cds_NP_212306.2_161 | BB_0172 | 2887.764892 | -1.217319862 | 0.123742701 | -9.837508402 | 7.76E-23 | 8.81E-21 |
| lcl\|NC_001851.2_cds_YP_004940410.1_1147 | BB_F0034 | 131.6802735 | -1.220309664 | 0.498446076 | -2.448228046 | 0.014356076 | 0.047564976 |
| lcl\|NC_001856.1_cds_NP_045643.1_1260 | BB_J19 | 8721.994493 | -1.226737445 | 0.357426229 | -3.43214164 | 0.000598835 | 0.00346876 |
| lcl\|NC_001318.1_cds_NP_212549.1_394 | BB_0415 | 1448.604328 | -1.231446613 | 0.221932825 | -5.548735809 | 2.88E-08 | 5.98E-07 |
| lcl\|NC_001849.2_cds_NP_045405.1_1120 | BB_D22 | 128.5625749 | -1.23419393 | 0.316273386 | -3.902300932 | 9.53E-05 | 0.000721183 |
| lcl\|NC_001318.1_cds_NP_212760.1_587 | rnmV | 17091.35904 | -1.237327813 | 0.410056749 | -3.017455062 | 0.002549068 | 0.011824118 |
| lcl\|NC_000951.1_cds_NP_051329.2_978 | erpK | 89.22936299 | -1.238045803 | 0.471227478 | -2.627278462 | 0.008607087 | 0.031253451 |
| lcl\|NC_001851.2_cds_NP_045444.1_1146 | BB_F08 | 152.5453178 | -1.253124952 | 0.415636758 | -3.014952189 | 0.002570198 | 0.011848455 |
| ncRNA0043 | A-(BB_0244) | 577.795707 | -1.256653342 | 0.403868558 | -3.111540418 | 0.00186114 | 0.009029975 |
| lcl\|NC_000952.1_cds_NP_051338.1_986 | BB_O05 | 56.56386104 | -1.292207756 | 0.465504454 | -2.775929952 | 0.005504407 | 0.021850826 |
| lcl\|NC_001856.1_cds_NP_045648.1_1264 | BB_J24 | 96.33456458 | -1.30740669 | 0.376420695 | -3.473259329 | 0.000514178 | 0.003045537 |
| ncRNA0231 | p-(BB_D20) | 3348.603578 | -1.31009764 | 0.387511439 | -3.380797334 | 0.000722758 | 0.004089227 |
| ncRNA0136 | AI-(BB_B09,BB_B09/BB_B10) | 476.369844 | -1.314926159 | 0.352213017 | -3.733326407 | 0.000188967 | 0.001302881 |
| lcl\|NC_001855.1_cds_NP_045624.1_1252 | BB_K53 | 359.8395733 | -1.319843975 | 0.48424027 | -2.725597306 | 0.006418525 | 0.024508405 |
| ncRNA0304 | I-(BB_J37/BB_J41) | 103.2796554 | -1.321009225 | 0.482436974 | -2.738200627 | 0.006177638 | 0.023910266 |
| ncRNA0031 | AA-(BB_0198,BB_0199) | 400.7134034 | -1.332232934 | 0.373955161 | -3.562547258 | 0.000367274 | 0.002360254 |
| ncRNA0168 | A-(BB_R43) | 73.05907492 | -1.339319655 | 0.46258358 | -2.89530306 | 0.003787926 | 0.016127096 |
| ncRNA0014 | A-(BB_0084) | 380.5460997 | -1.357896884 | 0.388753058 | -3.492954863 | 0.000477707 | 0.002884877 |
| lcl\|NC_001855.1_cds_NP_045612.1_1242 | BB_K40 | 3811.74482 | -1.359892512 | 0.231723983 | -5.868587679 | 4.40E-09 | 1.10E-07 |
| lcl\|NC_000951.1_cds_NP_051330.1_979 | BB_M39 | 174.8340308 | -1.364514032 | 0.518402401 | -2.632152222 | 0.008484584 | 0.031006966 |
| ncRNA0002 | AI-(BB_0004,BB_0004/BB_0005) | 3041.858416 | -1.36714353 | 0.248794138 | -5.495079342 | 3.91E-08 | 7.56E-07 |
| lcl\|NC_001318.1_cds_NP_212932.1_752 | BB_0798 | 412.7429471 | -1.367264622 | 0.230607112 | -5.928978559 | 3.05E-09 | 7.94E-08 |
| ncRNA0246 | I-(BB_E31/BB_E33) | 124.5161323 | -1.367831258 | 0.432304443 | -3.164046267 | 0.001555921 | 0.007725168 |
| ncRNA0037 | A-(BB_0211) | 1238.76926 | -1.369299269 | 0.314591832 | -4.352621806 | 1.35E-05 | 0.000134756 |
| lcl\|NC_001855.1_cds_NP_045606.1_1238 | BB_K33 | 37.62541137 | -1.379270088 | 0.444039976 | -3.106184497 | 0.001895184 | 0.009169031 |
| ncRNA0252 | Ip-(BB_F14/BB_F14a,BB_F14a) | 58.86305107 | -1.389686207 | 0.459183251 | -3.026430526 | 0.002474597 | 0.011514312 |
| lcl\|NC_001849.2_cds_NP_045397.1_1115 | BB_D13 | 908.2099786 | -1.394902701 | 0.304581916 | -4.57972922 | 4.66E-06 | 5.36E-05 |
| lcl\|NC_001855.1_cds_YP_004940637.1_1241 | BB_K0058 | 51.97230439 | -1.399070285 | 0.544398454 | -2.569938021 | 0.010171671 | 0.035977936 |
| lcl\|NC_000948.1_cds_NP_051196.1_855 | bppA | 59.0359037 | -1.406828985 | 0.558482981 | -2.519018542 | 0.011768246 | 0.040193419 |
| lcl\|NC_001318.1_cds_NP_212711.1_539 | BB_0577 | 1867.115855 | -1.438036835 | 0.222937044 | -6.450416729 | 1.12E-10 | 3.59E-09 |
| lcl\|NC_001903.1_cds_NP_047005.1_813 | ospC | 292.2425211 | -1.444790637 | 0.487067621 | -2.966304013 | 0.003014024 | 0.013507586 |
| ncRNA0229 | I-(BB_D18/BB_D20) | 117.8530042 | -1.450639654 | 0.345422551 | -4.199609001 | 2.67E-05 | 0.000248821 |
| ncRNA0353 | IA-(BB_Q85/BB_Q88,BB_Q88) | 66.89124586 | -1.49929536 | 0.616918906 | -2.430295692 | 0.015086509 | 0.049219013 |
| ncRNA0286 | A-(BB_K19) | 219.9417934 | -1.500183594 | 0.498015038 | -3.012325895 | 0.002592541 | 0.011876819 |
| lcl\|NC_000948.1_cds_NP_051197.1_856 | bppB | 16.41223164 | -1.500572432 | 0.595533131 | -2.519712763 | 0.011745063 | 0.040193419 |
| ncRNA0125 | AIA-(BB_0794,BB_0794/BB_0795,BB_0795) | 2242.036294 | -1.51380039 | 0.260264023 | -5.816402779 | 6.01E-09 | 1.42E-07 |
| ncRNA0325 | A-(BB_A66) | 457.9294428 | -1.540030266 | 0.339220102 | -4.539914515 | 5.63E-06 | 6.35E-05 |
| ncRNA0003 | AA-(BB_0005,BB_0006) | 1243.576562 | -1.545333022 | 0.311573702 | -4.959767184 | 7.06E-07 | 1.03E-05 |
| lcl\|NC_001853.1_cds_NP_045516.1_1196 | BB_H25 | 30.47678431 | -1.561052859 | 0.518258182 | -3.012114256 | 0.002594349 | 0.011876819 |
| lcl\|NC_001857.2_cds_NP_045738.1_1333 | BB_A65 | 185.7509576 | -1.571198149 | 0.496762792 | -3.162874059 | 0.001562199 | 0.007733793 |
| ncRNA0328 | I-(BB_A73/BB_A74) | 55.68358442 | -1.579146243 | 0.646463656 | -2.44274559 | 0.014576008 | 0.047920738 |
| lcl\|NC_001853.1_cds_NP_045517.1_1197 | BB_H26 | 1195.446691 | -1.591603607 | 0.4611763 | -3.451182569 | 0.000558136 | 0.003277605 |
| ncRNA0310 | Ip-(BB_J50/BB_J51,BB_J51) | 106.491945 | -1.600677163 | 0.607456387 | -2.635048699 | 0.008412521 | 0.030809728 |
| ncRNA0006 | A-(BB_0013) | 3033.894978 | -1.608698814 | 0.339632615 | -4.736585192 | 2.17E-06 | 2.74E-05 |
| lcl\|NC_000949.1_cds_NP_051234.2_890 | BB_S31 | 105.7674492 | -1.61576679 | 0.504778499 | -3.200942183 | 0.00136979 | 0.006963441 |
| ncRNA0300 | I-(BB_J20/BB_J0058) | 169.4427612 | -1.622166436 | 0.5479595 | -2.960376517 | 0.003072633 | 0.013686553 |
| ncRNA0063 | A-(BB_0381) | 391.0552137 | -1.635832191 | 0.433065836 | -3.777329118 | 0.000158519 | 0.001120158 |
| lcl\|NC_001855.1_cds_NP_045623.1_1251 | BB_K52 | 62.12418253 | -1.650671092 | 0.586187285 | -2.815944895 | 0.004863401 | 0.020054168 |
| lcl\|NC_000948.1_cds_NP_051171.1_830 | BB_P10 | 89.29313498 | -1.665579649 | 0.568149994 | -2.931584381 | 0.003372377 | 0.014650915 |
| lcl\|NC_000953.1_cds_NP_051387.1_1034 | BB_L10 | 89.29313498 | -1.665579649 | 0.568149994 | -2.931584381 | 0.003372377 | 0.014650915 |
| ncRNA0080 | p-(BB_0522) | 879.8130905 | -1.668711458 | 0.235879187 | -7.074432798 | 1.50E-12 | 6.73E-11 |
| ncRNA0263 | IA-(BB_G28/BB_G29,BB_G29) | 24.0789363 | -1.668736711 | 0.650242423 | -2.566330111 | 0.010278096 | 0.036239332 |
| ncRNA0281 | IpI-(BB_K09/BB_K10,BB_K10,BB_K10/BB_K12) | 166.2483876 | -1.672741729 | 0.505577971 | -3.308573206 | 0.000937727 | 0.005053634 |
| ncRNA0248 | AIP-(BB_F03,BB_F03/BB_F05,BB_F05) | 239.2275787 | -1.691341153 | 0.556097881 | -3.041445059 | 0.002354455 | 0.010985308 |
| ncRNA0050 | AIA-(BB_0269,BB_0269/BB_0270,BB_0270) | 1285.298201 | -1.696511444 | 0.556125738 | -3.050589691 | 0.002283925 | 0.010685504 |
| ncRNA0289 | AIA-(BB_K33,BB_K33/BB_K34,BB_K34) | 54.0182385 | -1.710605599 | 0.416989275 | -4.10227721 | 4.09E-05 | 0.000348352 |
| ncRNA0285 | A-(BB_K19) | 59.46915813 | -1.710662288 | 0.514990052 | -3.321738508 | 0.000894585 | 0.004882943 |
| ncRNA0299 | I-(BB_J20/BB_J0058) | 119.1266624 | -1.713102967 | 0.322707732 | -5.308527805 | 1.11E-07 | 1.90E-06 |
| lcl\|NC_001849.2_cds_NP_045398.1_1116 | BB_D14 | 3487.083186 | -1.744927691 | 0.326860939 | -5.338440546 | 9.37E-08 | 1.66E-06 |
| lcl\|NC_001855.1_cds_NP_045597.1_1234 | BB_K23 | 2453.836139 | -1.76010754 | 0.38683042 | -4.550075304 | 5.36E-06 | 6.09E-05 |

| ncRNA0200 | IA-(BB_L29/BB_L30,BB_L30) | 867.0447963 | -1.763677237 | 0.352133584 | -5.008545957 | 5.48E-07 | 8.12E-06 |
| --- | --- | --- | --- | --- | --- | --- | --- |
| lcl\|NC_001853.1_cds_NP_045498.1_1188 | BB_H04 | 146.0244758 | -1.764826058 | 0.517657817 | -3.40925221 | 0.000651412 | 0.003760526 |
| ncRNA0271 | P-(BB_H30) | 22.38915613 | -1.767197173 | 0.655615851 | -2.695476582 | 0.007028802 | 0.026365748 |
| lcl\|NC_001318.1_cds_NP_212722.1_550 | BB_0588 | 1645.236707 | -1.769614933 | 0.130872183 | -13.52170412 | 1.16E-41 | 3.31E-39 |
| lcl\|NC_001855.1_cds_NP_045598.1_1235 | BB_K24 | 377.5769784 | -1.774945995 | 0.437272966 | -4.05912584 | 4.93E-05 | 0.000405238 |
| lcl\|NC_001857.2_cds_YP_004940408.1_1337 | BB_A0078 | 99.0563379 | -1.7817888 | 0.581545432 | -3.063885815 | 0.002184823 | 0.010300298 |
| lcl\|NC_000951.1_cds_NP_051301.1_951 | BB_M10 | 26.57919292 | -1.783800995 | 0.552979602 | -3.225798903 | 0.001256216 | 0.00650254 |
| ncRNA0070 | A-(BB_0446) | 529.3172145 | -1.785175913 | 0.312674426 | -5.709376155 | 1.13E-08 | 2.54E-07 |
| lcl\|NC_001318.1_cds_NP_212161.1_25 | BB_0027 | 9710.085949 | -1.78806112 | 0.33743615 | -5.298961365 | 1.16E-07 | 1.96E-06 |
| ncRNA0187 | A-(BB_O36) | 30.42655709 | -1.803465374 | 0.720330268 | -2.503664573 | 0.01229145 | 0.041402801 |
| lcl\|NC_001857.2_cds_NP_045746.1_1338 | BB_A73 | 233.2233394 | -1.829096851 | 0.424452475 | -4.309308956 | 1.64E-05 | 0.000159853 |
| ncRNA0242 | I-(BB_E23b/BB_E29a) | 107.0500482 | -1.832009291 | 0.592483694 | -3.092083897 | 0.001987567 | 0.009534721 |
| ncRNA0084 | A-(BB_0581) | 87.52936081 | -1.86382887 | 0.465310745 | -4.005557342 | 6.19E-05 | 0.000490079 |
| lcl\|NC_001855.1_cds_NP_045605.1_1237 | BB_K32 | 213.6510062 | -1.873429774 | 0.489655712 | -3.826014332 | 0.000130235 | 0.000943786 |
| ncRNA0245 | I-(BB_E31/BB_E33) | 314.7614859 | -1.875521284 | 0.485353166 | -3.864240341 | 0.000111435 | 0.000825107 |
| lcl\|NC_001850.1_cds_NP_045436.1_1141 | BB_E31 | 93.10926862 | -1.879099504 | 0.381704541 | -4.922916288 | 8.53E-07 | 1.21E-05 |
| ncRNA0042 | A-(BB_0240) | 2021.458735 | -1.879991865 | 0.349110627 | -5.385089195 | 7.24E-08 | 1.33E-06 |
| ncRNA0132 | pI-(BB_0845a,BB_0845a/BB_0845b) | 211.2546476 | -1.882309932 | 0.534541277 | -3.521355622 | 0.000429346 | 0.002649192 |
| ncRNA0057 | A-(BB_0347) | 34.51259723 | -1.887737468 | 0.548599499 | -3.441012019 | 0.000579543 | 0.003368469 |
| lcl\|NC_001849.2_cds_NP_045404.1_1119 | BB_D21 | 967.6859329 | -1.895897547 | 0.440022711 | -4.308635668 | 1.64E-05 | 0.000159853 |
| lcl\|NC_001857.2_cds_NP_045710.1_1311 | BB_A37 | 93.94411642 | -1.91040809 | 0.652530567 | -2.927691339 | 0.003414889 | 0.01477655 |
| lcl\|NC_001852.1_cds_NP_045481.1_1172 | BB_G21 | 257.5363142 | -1.920188356 | 0.710774817 | -2.701542473 | 0.006901866 | 0.02611973 |
| lcl\|NC_000948.1_cds_NP_051190.1_849 | BB_P29 | 117.5902665 | -1.925349814 | 0.651102731 | -2.95705996 | 0.003105877 | 0.013738464 |
| ncRNA0322 | I-(BB_A37/BB_A38) | 2507.453992 | -1.934721258 | 0.464555498 | -4.164671965 | 3.12E-05 | 0.000276561 |
| lcl\|NC_001856.1_cds_NP_045667.1_1276 | BB_J43 | 43.39693922 | -1.947979145 | 0.670370858 | -2.905823132 | 0.003662884 | 0.015712574 |
| lcl\|NC_000952.1_cds_NP_051372.1_1020 | erpL | 12.95799511 | -1.949705703 | 0.71377829 | -2.731528444 | 0.00630413 | 0.02418003 |
| lcl\|NC_001855.1_cds_YP_004940636.1_1236 | BB_K54 | 131.6013456 | -1.957517875 | 0.652698517 | -2.999114942 | 0.002707651 | 0.01236228 |
| ncRNA0153 | AIA-(BB_P35,BB_P35/BB_P36,BB_P36) | 34.01766031 | -1.98207391 | 0.798737356 | -2.481508965 | 0.013082742 | 0.043686097 |
| ncRNA0087 | A-(BB_0588) | 1536.156421 | -1.983017812 | 0.709181109 | -2.796207891 | 0.005170613 | 0.020915805 |
| ncRNA0253 | pIp-(BB_F14a,BB_F14a/BB_F16,BB_F16) | 16.1459582 | -1.986715918 | 0.793616815 | -2.503369235 | 0.012301713 | 0.041402801 |
| lcl\|NC_001851.2_cds_NP_045458.1_1153 | BB_F25 | 156.0679977 | -1.989776392 | 0.451280505 | -4.409178704 | 1.04E-05 | 0.000109757 |
| lcl\|NC_001851.2_cds_YP_004940409.1_1142 | arp | 9.140034358 | -1.998405324 | 0.818669073 | -2.441041673 | 0.014644965 | 0.047971938 |
| ncRNA0287 | I-(BB_K55/BB_K56) | 86.5607051 | -2.023857127 | 0.572688859 | -3.53395582 | 0.00040939 | 0.0025632 |
| ncRNA0318 | IP-(BB_A16/BB_A18,BB_A18) | 1800.37672 | -2.047895874 | 0.566676993 | -3.613868038 | 0.000301663 | 0.001983519 |
| ncRNA0099 | A-(BB_0633) | 150.8640122 | -2.112142504 | 0.596785888 | -3.539196463 | 0.000401347 | 0.002531459 |
| lcl\|NC_001851.2_cds_NP_045457.1_1152 | BB_F24 | 283.2747783 | -2.12742045 | 0.368574807 | -5.772018074 | 7.83E-09 | 1.78E-07 |
| lcl\|NC_001857.2_cds_NP_045709.2_1310 | BB_A36 | 46.52933338 | -2.130484898 | 0.582127955 | -3.659822345 | 0.00025239 | 0.00168557 |
| lcl\|NC_001857.2_cds_NP_045707.1_1309 | BB_A34 | 190.0838372 | -2.164189119 | 0.577886296 | -3.745008546 | 0.000180388 | 0.001253343 |
| ncRNA0255 | I-(BB_F0040/BB_F32) | 49.42843423 | -2.200046006 | 0.637668726 | -3.450139414 | 0.000560297 | 0.00327899 |
| lcl\|NC_001853.1_cds_NP_045510.1_1195 | BB_H17 | 10.29555978 | -2.234942039 | 0.800322419 | -2.792552085 | 0.005229405 | 0.02096611 |
| lcl\|NC_001851.2_cds_NP_045442.1_1145 | BB_F06 | 11.18133266 | -2.261136123 | 0.827384713 | -2.732871528 | 0.006278482 | 0.024136014 |
| ncRNA0284 | A-(BB_K17) | 123.8271122 | -2.273935919 | 0.296721246 | -7.663542628 | 1.81E-14 | 8.80E-13 |
| lcl\|NC_001851.2_cds_NP_045439.1_1144 | repU | 189.0840177 | -2.286930833 | 0.378577934 | -6.040845561 | 1.53E-09 | 4.21E-08 |
| ncRNA0297 | A-(BB_J18) | 206.5236032 | -2.307138732 | 0.688145025 | -3.352692597 | 0.000800295 | 0.004439424 |
| lcl\|NC_001852.1_cds_NP_045472.1_1163 | BB_G12 | 81.95387397 | -2.359314711 | 0.670799011 | -3.517170827 | 0.000436173 | 0.002671952 |
| ncRNA0239 | A-(BB_E09) | 1464.223554 | -2.363199357 | 0.428192961 | -5.519005611 | 3.41E-08 | 6.91E-07 |
| ncRNA0249 | pI-(BB_F05,BB_F05/BB_F06) | 9.67900222 | -2.374420185 | 0.741158663 | -3.203659762 | 0.001356927 | 0.006931911 |
| lcl\|NC_001851.2_cds_YP_004940411.1_1149 | BB_F17 | 21.28080946 | -2.380548185 | 0.759226444 | -3.13549166 | 0.001715663 | 0.008444434 |
| lcl\|NC_001851.2_cds_YP_004940414.1_1157 | BB_F0041 | 431.5944656 | -2.393300878 | 0.516787972 | -4.63110794 | 3.64E-06 | 4.27E-05 |
| ncRNA0144 | AIA-(BB_P01,BB_P01/BB_P02,BB_P02) | 5.386579535 | -2.405247545 | 0.855087975 | -2.812865595 | 0.004910217 | 0.020149638 |
| lcl\|NC_000950.1_cds_NP_051282.1_934 | bppA | 20.6580321 | -2.419159274 | 0.672161892 | -3.599072338 | 0.000319354 | 0.002075803 |
| lcl\|NC_001856.1_cds_NP_045647.2_1263 | BB_J23 | 37.83775028 | -2.452974412 | 0.487590759 | -5.030805791 | 4.88E-07 | 7.31E-06 |
| ncRNA0344 | A-(BB_Q52) | 10.93288614 | -2.492065144 | 0.858939246 | -2.901328767 | 0.003715838 | 0.01585983 |
| lcl\|NC_000949.1_cds_NP_051241.2_895 | bppA | 41.41867394 | -2.507569395 | 0.644486353 | -3.890802937 | 9.99E-05 | 0.000752885 |
| lcl\|NC_001318.1_cds_NP_212828.2_653 | ffh | 14060.76269 | -2.511684041 | 0.374188652 | -6.71234691 | 1.92E-11 | 7.77E-10 |
| ncRNA0191 | A-(BB_O44) | 8.708649435 | -2.530781759 | 0.797617394 | -3.172926993 | 0.001509105 | 0.007514635 |
| ncRNA0233 | p-(BB_D23) | 46.44975772 | -2.615412768 | 0.750703561 | -3.483948797 | 0.000494074 | 0.002973173 |
| lcl\|NC_001857.2_cds_NP_045698.1_1304 | dbpB | 89.46809468 | -2.646999244 | 0.558325827 | -4.740957908 | 2.13E-06 | 2.70E-05 |
| lcl\|NC_001852.1_cds_NP_045482.1_1174 | BB_G22 | 64.42227065 | -2.664763491 | 0.638466233 | -4.17369526 | 3.00E-05 | 0.000268625 |
| lcl\|NC_001851.2_cds_NP_045459.1_1154 | BB_F26 | 1080.037774 | -2.756403353 | 0.444938874 | -6.195015798 | 5.83E-10 | 1.68E-08 |
| lcl\|NC_001849.2_cds_YP_004940417.1_1121 | BB_D0031 | 25.13306541 | -2.773679485 | 0.70351362 | -3.942609505 | 8.06E-05 | 0.000626765 |
| lcl\|NC_001851.2_cds_NP_045453.2_1150 | BB_F20 | 136.227553 | -2.849065131 | 0.295141639 | -9.653213099 | 4.76E-22 | 4.77E-20 |
| lcl\|NC_001318.1_cds_NP_212643.1_482 | BB_0509 | 8232.921912 | -2.925501491 | 0.365731686 | -7.999037549 | 1.25E-15 | 6.89E-14 |
| ncRNA0250 | PI-(BB_F11a,BB_F11a/BB_F12) | 15.60876515 | -2.95590859 | 0.733796314 | -4.028241261 | 5.62E-05 | 0.000453887 |
| ncRNA0240 | A-(BB_E09) | 74.83749 | -2.991500413 | 0.853438126 | -3.50523409 | 0.000456206 | 0.002764835 |
| ncRNA0247 | A-(BB_F03) | 1699.002338 | -3.043049689 | 0.293976557 | -10.35133453 | 4.13E-25 | 5.41E-23 |
| ncRNA0257 | PI-(BB_G05,BB_G05/BB_G06) | 56.23393737 | -3.085696694 | 0.788709911 | -3.912334118 | 9.14E-05 | 0.000694948 |
| lcl\|NC_001851.2_cds_NP_045449.2_1148 | BB_F14 | 47.65068541 | -3.144088867 | 0.736154608 | -4.270962693 | 1.95E-05 | 0.000186747 |
| ncRNA0251 | I-(BB_F11a/BB_F12) | 727.7453318 | -3.628950353 | 0.253865442 | -14.29477888 | 2.36E-46 | 1.00E-43 |
| ncRNA0071 | A-(BB_0450) | 65.28090439 | -4.683065312 | 0.697311108 | -6.715890878 | 1.87E-11 | 7.76E-10 |
| ncRNA0226 | P-(BB_D05a) | 56.8286166 | -5.381016157 | 0.67963523 | -7.917506215 | 2.42E-15 | 1.29E-13 |
| ncRNA0308 | I-(BB_J50/BB_J51) | 893.058206 | -5.421448561 | 0.494839696 | -10.95596938 | 6.22E-28 | 1.06E-25 |
