# Supplementary material for: Transcriptomic insights on the virulence-controlling CsrA, BadR, RpoN, and RpoS regulatory networks in the Lyme disease spirochete
Source: PLoS One. 2018 Aug 30;13(8):e0203286. doi: 10.1371/journal.pone.0203286 (PMC6117026; doi:10.1371/journal.pone.0203286)
Supplement: S3 Table — The included transcripts met the criteria of >1 log2 fold-change and an adjusted p-value (padj) when comparing the badR mutant to wild-type sorted by fold change. A total of 234 transcripts were differentially regulated, not including the mutated gene, by the mutation. The first column contains the CDS/custom transcript ID which is the transcript ID for all coding sequences obtained from the NCBI Gene file format file or the transcript ID given to ncRNAs. RefSeq entries are further separated by the character “_”. The first portion gives the genetic element from which it is derived, the second describes the type of element (CDS), the third provides RefSeq ID, and the fourth provides a number indicating the particular entries ordered number in the RefSeq entry. The second column is the gene information, for the ncRNAs it contains the location relative to other genes and for predicted or known genes it contains gene name. The remaining columns describe the various metrics of expression of each impacted transcript including, base mean (average library size normalized counts across all samples), log2FC (Fold change estimate), lfcSE (uncertainty of the log fold change estimate), stat (Wald statistic), pvalue, padj (pvalue following Benjamini-Hochberg adjustment). ORFs and ncRNAs are identified according to the names or numbers assigned to genes and transcripts by the initial genome sequencing of B. burgdorferi strain B31 [18, 31] or from our previous analyses of that strain’s ncRNA transcriptome [30]. (DOCX) [file pone.0203286.s004.docx]

**Table S4: Differentially expressed transcripts when comparing ∆badR mutant to wild-type, listed in order of fold change.** The included transcripts met the criteria of >1 log2 fold-change and an adjusted p-value (padj) when comparing the ∆*badR* mutant to wild-type sorted by fold change. A total of 234 transcripts were differentially regulated, not including the mutated gene, by the mutation. The first column contains the CDS/custom transcript ID which is the transcript ID for all coding sequences obtained from the NCBI Gene file format file or the transcript ID given to ncRNAs. RefSeq entries are further separated by the character “_”. The first portion gives the genetic element from which it is derived, the second describes the type of element (CDS), the third provides RefSeq ID, and the fourth provides a number indicating the particular entries ordered number in the RefSeq entry. The second column is the gene information, for the ncRNAs it contains the location relative to other genes and for predicted or known genes it contains gene name. The remaining columns describe the various metrics of expression of each impacted transcript including, base mean (average library size normalized counts across all samples), log2FC (Fold change estimate), lfcSE (uncertainty of the log fold change estimate), stat (Wald statistic), pvalue, padj (pvalue following Benjamini-Hochberg adjustment). ORFs and ncRNAs are identified according to the names or numbers assigned to genes and transcripts by the initial genome sequencing of *B. burgdorferi* strain B31 [18, 31] or from our previous analyses of that strain’s ncRNA transcriptome [30].

| **RefSeq CDS/Custom Transcript ID** | **Gene Name** | **baseMean** | **log2FoldChange** | **lfcSE** | **stat** | **pvalue** | **padj** |
| --- | --- | --- | --- | --- | --- | --- | --- |
| lcl\|NC_001903.1_cds_NP_046992.2_803 | chbB | 1687.198719 | 6.041919219 | 0.262574866 | 23.01027252 | 3.68E-117 | 2.09E-114 |
| lcl\|NC_001903.1_cds_NP_046990.2_801 | chbC | 11469.16755 | 5.577232622 | 0.223856 | 24.91437634 | 5.20E-137 | 4.43E-134 |
| lcl\|NC_001903.1_cds_NP_046991.1_802 | chbA | 2551.066468 | 5.136410472 | 0.275411401 | 18.64995587 | 1.26E-77 | 4.30E-75 |
| lcl\|NC_001903.1_cds_NP_046993.1_804 | BB_B07 | 8409.809781 | 4.824049857 | 0.162670624 | 29.6553228 | 2.90E-193 | 4.93E-190 |
| ncRNA0135 | I-(BB_B03/BB_B04) | 1218.809536 | 3.522740679 | 0.296249536 | 11.89112641 | 1.32E-32 | 1.49E-30 |
| lcl\|NC_001318.1_cds_NP_212975.1_793 | arcA | 2690.756525 | 3.208277689 | 0.156554928 | 20.49298436 | 2.49E-93 | 1.06E-90 |
| lcl\|NC_000956.1_cds_NP_051521.1_1385 | BB_Q62 | 20.48709362 | 3.14255226 | 0.535260529 | 5.871070419 | 4.33E-09 | 7.52E-08 |
| ncRNA0072 | A-(BB_0454) | 396.651084 | 2.955889742 | 0.659489457 | 4.48208794 | 7.39E-06 | 6.29E-05 |
| lcl\|NC_001318.1_cds_NP_212976.2_794 | arcB | 2452.381364 | 2.753000269 | 0.247532876 | 11.12175607 | 9.83E-29 | 8.37E-27 |
| lcl\|NC_001318.1_cds_NP_212375.1_226 | glpK | 5175.056984 | 2.392006594 | 0.337870592 | 7.079653128 | 1.45E-12 | 4.17E-11 |
| lcl\|NC_000956.1_cds_NP_051504.1_1374 | bdrV | 634.196218 | 2.372377131 | 0.317623917 | 7.469138837 | 8.07E-14 | 2.81E-12 |
| lcl\|NC_001857.2_cds_NP_045676.1_1287 | BB_A03 | 12443.29488 | 2.365156998 | 0.336486898 | 7.028972045 | 2.08E-12 | 5.91E-11 |
| lcl\|NC_000949.1_cds_NP_051218.1_876 | BB_S15 | 10.07082847 | 2.315424388 | 0.806609874 | 2.870562912 | 0.004097417 | 0.016733574 |
| lcl\|NC_001851.2_cds_NP_045456.1_1151 | BB_F23 | 367.9227399 | 2.20505297 | 0.256947467 | 8.581726827 | 9.35E-18 | 4.55E-16 |
| lcl\|NC_001318.1_cds_NP_212376.1_227 | BB_0242 | 990.1464708 | 2.168283941 | 0.419660091 | 5.166762312 | 2.38E-07 | 2.70E-06 |
| lcl\|NC_000956.1_cds_NP_051489.1_1361 | BB_Q27 | 184.692011 | 2.143457033 | 0.446777313 | 4.797595967 | 1.61E-06 | 1.56E-05 |
| ncRNA0311 | A-(BB_A04) | 361.981407 | 2.135911713 | 0.411274034 | 5.19340278 | 2.06E-07 | 2.38E-06 |
| ncRNA0152 | IA-(BB_P32/BB_P33,BB_P33) | 276.3515173 | 2.117378095 | 0.365073141 | 5.799873664 | 6.64E-09 | 1.13E-07 |
| ncRNA0134 | AI-(BB_B03,BB_B03/BB_B04) | 130.5038159 | 2.104778754 | 0.376423997 | 5.591510561 | 2.25E-08 | 3.24E-07 |
| lcl\|NC_001857.2_cds_NP_045747.1_1339 | osm28 | 12273.60539 | 2.102644849 | 0.394215493 | 5.333744837 | 9.62E-08 | 1.20E-06 |
| lcl\|NC_000948.1_cds_NP_051194.2_853 | BB_P33 | 507.5530008 | 2.041103905 | 0.245742172 | 8.305875571 | 9.91E-17 | 4.44E-15 |
| ncRNA0218 | IA-(BB_N32/BB_N33,BB_N33) | 29.8614237 | 2.012668676 | 0.517685749 | 3.887819354 | 0.000101149 | 0.000695772 |
| lcl\|NC_001318.1_cds_NP_212377.1_228 | glpD | 6087.037737 | 2.007251927 | 0.236394298 | 8.491118202 | 2.05E-17 | 9.68E-16 |
| lcl\|NC_001318.1_cds_NP_212977.2_795 | BB_0843 | 9954.801279 | 2.005127584 | 0.225005605 | 8.911456163 | 5.04E-19 | 2.68E-17 |
| lcl\|NC_001857.2_cds_NP_045704.1_1306 | BB_A31 | 693.6564221 | 1.993259822 | 0.268904006 | 7.412533024 | 1.24E-13 | 4.14E-12 |
| lcl\|NC_001318.1_cds_NP_212499.1_346 | la7 | 8401.745644 | 1.980278708 | 0.282326106 | 7.014153714 | 2.31E-12 | 6.46E-11 |
| lcl\|NC_001857.2_cds_NP_045727.1_1325 | BB_A54 | 599.9624458 | 1.952102454 | 0.367103617 | 5.317578918 | 1.05E-07 | 1.29E-06 |
| lcl\|NC_000954.1_cds_NP_051445.1_1089 | BB_N33 | 157.3216497 | 1.90141208 | 0.288793883 | 6.583976291 | 4.58E-11 | 1.11E-09 |
| lcl\|NC_001318.1_cds_NP_212498.1_345 | mgsA | 3019.346339 | 1.88485369 | 0.164726999 | 11.44228752 | 2.57E-30 | 2.57E-28 |
| lcl\|NC_000948.1_cds_NP_051193.1_852 | BB_P32 | 812.8429087 | 1.854983089 | 0.23580194 | 7.866699848 | 3.64E-15 | 1.35E-13 |
| lcl\|NC_000956.1_cds_NP_051469.1_1342 | BB_Q05 | 100.9367965 | 1.796704482 | 0.595838392 | 3.015422481 | 0.002566215 | 0.011410613 |
| lcl\|NC_001851.2_cds_NP_045439.1_1144 | repU | 189.0840177 | 1.753939584 | 0.35530882 | 4.936380643 | 7.96E-07 | 8.47E-06 |
| lcl\|NC_000954.1_cds_NP_051444.1_1088 | BB_N32 | 241.9969265 | 1.728633599 | 0.308943605 | 5.595304672 | 2.20E-08 | 3.21E-07 |
| lcl\|NC_001855.1_cds_NP_045618.1_1246 | BB_K47 | 2505.671645 | 1.677219339 | 0.317042319 | 5.290206506 | 1.22E-07 | 1.47E-06 |
| ncRNA0186 | AIA-(BB_O32,BB_O32/BB_O33,BB_O33) | 90.75800452 | 1.658067092 | 0.325706168 | 5.090683735 | 3.57E-07 | 3.92E-06 |
| lcl\|NC_001855.1_cds_NP_045620.1_1248 | BB_K49 | 1392.93963 | 1.632033985 | 0.354485887 | 4.603946289 | 4.15E-06 | 3.76E-05 |
| lcl\|NC_001857.2_cds_NP_045733.1_1329 | BB_A60 | 1031.73146 | 1.630327073 | 0.221726805 | 7.352864153 | 1.94E-13 | 6.12E-12 |
| ncRNA0073 | I-(BB_t06/BB_0461) | 2784.933991 | 1.60500746 | 0.229931003 | 6.980387322 | 2.94E-12 | 8.09E-11 |
| lcl\|NC_001318.1_cds_NP_212919.1_739 | spoVG | 2664.703766 | 1.601558347 | 0.21191112 | 7.557689027 | 4.10E-14 | 1.46E-12 |
| lcl\|NC_000954.1_cds_NP_051443.1_1087 | BB_N31 | 161.0855567 | 1.597055987 | 0.349207277 | 4.57337545 | 4.80E-06 | 4.30E-05 |
| lcl\|NC_000954.1_cds_NP_051446.1_1090 | bdrQ | 227.796319 | 1.584969312 | 0.264083749 | 6.001767683 | 1.95E-09 | 3.69E-08 |
| lcl\|NC_001857.2_cds_NP_045703.1_1305 | BB_A30 | 869.6149934 | 1.582224773 | 0.216857972 | 7.296133785 | 2.96E-13 | 9.01E-12 |
| ncRNA0326 | I-(BB_A66/BB_A68) | 456.2996811 | 1.581317957 | 0.367259293 | 4.305726189 | 1.66E-05 | 0.000133701 |
| lcl\|NC_000956.1_cds_NP_051494.1_1366 | BB_Q32 | 73.6825877 | 1.568087947 | 0.471959354 | 3.322506342 | 0.000892126 | 0.004792717 |
| lcl\|NC_000950.1_cds_NP_051272.1_925 | BB_R25 | 73.6903149 | 1.567845055 | 0.47257634 | 3.317654571 | 0.000907767 | 0.004806509 |
| lcl\|NC_000951.1_cds_NP_051316.1_965 | BB_M25 | 73.66910533 | 1.56778483 | 0.472774075 | 3.316139594 | 0.000912702 | 0.004806509 |
| lcl\|NC_001318.1_cds_NP_212901.1_720 | murG | 901.267597 | 1.560828217 | 0.281070942 | 5.553146858 | 2.81E-08 | 3.98E-07 |
| ncRNA0185 | I-(BB_O29/BB_O30) | 1573.01516 | 1.545721747 | 0.477736479 | 3.235511242 | 0.001214251 | 0.006154374 |
| lcl\|NC_000952.1_cds_NP_051368.1_1016 | BB_O35 | 19.61631666 | 1.531544803 | 0.614609961 | 2.491897136 | 0.012706283 | 0.043592704 |
| lcl\|NC_000956.1_cds_NP_051491.1_1363 | BB_Q29 | 93.20450698 | 1.487257023 | 0.362277235 | 4.105300802 | 4.04E-05 | 0.000296402 |
| lcl\|NC_000948.1_cds_NP_051195.1_854 | bdrA | 840.2082301 | 1.459685689 | 0.302686653 | 4.822431625 | 1.42E-06 | 1.40E-05 |
| lcl\|NC_001855.1_cds_NP_045575.1_1225 | BB_K01 | 1354.450581 | 1.456025551 | 0.334102075 | 4.358026067 | 1.31E-05 | 0.000105926 |
| lcl\|NC_001856.1_cds_NP_045633.1_1254 | BB_J09 | 24951.96368 | 1.396908601 | 0.377302455 | 3.70235757 | 0.000213605 | 0.001388434 |
| lcl\|NC_000949.1_cds_NP_051240.1_894 | bdrE | 326.736536 | 1.384619643 | 0.242365624 | 5.712937421 | 1.11E-08 | 1.78E-07 |
| ncRNA0337 | I-(BB_Q37/BB_Q38) | 248.2040394 | 1.382053001 | 0.42404404 | 3.259220435 | 0.001117188 | 0.005696322 |
| lcl\|NC_001857.2_cds_NP_045726.1_1324 | BB_A53 | 375.6912082 | 1.380553161 | 0.375841734 | 3.673230076 | 0.000239504 | 0.001533364 |
| lcl\|NC_001318.1_cds_NP_212772.1_598 | BB_0638 | 3886.718713 | 1.374480998 | 0.209927381 | 6.547411741 | 5.85E-11 | 1.38E-09 |
| lcl\|NC_001318.1_cds_NP_212542.1_389 | fruA1 | 6219.62582 | 1.359858126 | 0.22210754 | 6.122521208 | 9.21E-10 | 1.84E-08 |
| lcl\|NC_001318.1_cds_NP_212900.1_719 | cvpA | 420.5109223 | 1.356521949 | 0.239595391 | 5.661719703 | 1.50E-08 | 2.30E-07 |
| lcl\|NC_000948.1_cds_NP_051192.1_851 | BB_P31 | 319.4277675 | 1.343678557 | 0.331802576 | 4.04963268 | 5.13E-05 | 0.000367062 |
| lcl\|NC_001857.2_cds_NP_045731.1_1327 | BB_A58 | 8350.064089 | 1.319104334 | 0.296677938 | 4.446250175 | 8.74E-06 | 7.37E-05 |
| lcl\|NC_001318.1_cds_NP_212904.1_723 | BB_0770 | 1364.054451 | 1.297246253 | 0.244604596 | 5.303441851 | 1.14E-07 | 1.37E-06 |
| ncRNA0327 | I-(BB_A68/BB_A69) | 288.5694033 | 1.264266587 | 0.411522547 | 3.072168452 | 0.002125098 | 0.009754828 |
| lcl\|NC_000956.1_cds_NP_051502.1_1373 | BB_Q40 | 481.1703282 | 1.262062785 | 0.368213247 | 3.427532266 | 0.000609094 | 0.003469187 |
| lcl\|NC_001318.1_cds_NP_212903.1_722 | BB_0769 | 1653.77636 | 1.261924875 | 0.240959955 | 5.237073006 | 1.63E-07 | 1.93E-06 |
| lcl\|NC_001318.1_cds_NP_212300.1_155 | malQ | 848.9299321 | 1.252229151 | 0.320511286 | 3.906973652 | 9.35E-05 | 0.000646997 |
| lcl\|NC_001318.1_cds_NP_212771.2_597 | BB_0637 | 8048.197727 | 1.247308866 | 0.261337592 | 4.772787777 | 1.82E-06 | 1.75E-05 |
| lcl\|NC_001318.1_cds_NP_212464.1_313 | BB_0330 | 9233.475103 | 1.246831348 | 0.219007994 | 5.693086005 | 1.25E-08 | 1.95E-07 |
| lcl\|NC_000954.1_cds_NP_051450.1_1094 | erpQ | 1968.305688 | 1.240874035 | 0.360078608 | 3.446119836 | 0.000568698 | 0.003260919 |
| lcl\|NC_001318.1_cds_NP_212907.1_727 | BB_0773 | 467.79866 | 1.23579489 | 0.179319285 | 6.891589423 | 5.52E-12 | 1.49E-10 |
| lcl\|NC_001318.1_cds_NP_212737.1_565 | p66 | 18788.96545 | 1.191518505 | 0.370846197 | 3.212972161 | 0.00131369 | 0.006618978 |
| lcl\|NC_001857.2_cds_NP_045677.1_1288 | BB_A04 | 322.0576528 | 1.186683617 | 0.439379821 | 2.700815014 | 0.00691698 | 0.026711149 |
| lcl\|NC_000952.1_cds_NP_051373.1_1021 | erpM | 506.3123838 | 1.178636168 | 0.240991847 | 4.890771956 | 1.00E-06 | 1.03E-05 |
| lcl\|NC_001318.1_cds_NP_212169.1_33 | BB_0035 | 2717.907721 | 1.16654414 | 0.214712484 | 5.433052237 | 5.54E-08 | 7.33E-07 |
| ncRNA0150 | I-(BB_P29/BB_P30) | 412.4991435 | 1.151296559 | 0.448344914 | 2.567881385 | 0.010232216 | 0.036685188 |
| lcl\|NC_001318.1_cds_NP_212815.2_641 | BB_0681 | 1841.564157 | 1.132095989 | 0.198715125 | 5.697080115 | 1.22E-08 | 1.92E-07 |
| lcl\|NC_001318.1_cds_NP_212812.1_638 | BB_0678 | 5061.700522 | 1.130878673 | 0.184670586 | 6.123761744 | 9.14E-10 | 1.84E-08 |
| lcl\|NC_001318.1_cds_NP_212773.1_599 | potD | 1482.689456 | 1.111387661 | 0.287042753 | 3.871854103 | 0.000108011 | 0.000732837 |
| lcl\|NC_001857.2_cds_NP_045705.1_1307 | BB_A32 | 110.7421883 | 1.110559517 | 0.324430366 | 3.42310595 | 0.000619099 | 0.003514421 |
| ncRNA0259 | AA-(BB_G07,BB_G08) | 88.149747 | 1.10442889 | 0.263139801 | 4.197118353 | 2.70E-05 | 0.000205525 |
| lcl\|NC_001318.1_cds_NP_212374.1_225 | glpF | 2634.820779 | 1.096128043 | 0.214494113 | 5.110294302 | 3.22E-07 | 3.58E-06 |
| lcl\|NC_001318.1_cds_NP_212813.2_639 | BB_0679 | 4281.290839 | 1.092892006 | 0.192500112 | 5.677357764 | 1.37E-08 | 2.12E-07 |
| lcl\|NC_001318.1_cds_NP_212778.1_604 | nanE | 697.3884376 | 1.091911248 | 0.199286939 | 5.479090873 | 4.28E-08 | 5.82E-07 |
| lcl\|NC_001318.1_cds_NP_212899.1_718 | BB_0765 | 561.7600503 | 1.079796925 | 0.175287443 | 6.160149906 | 7.27E-10 | 1.51E-08 |
| lcl\|NC_001857.2_cds_NP_045725.1_1323 | BB_A52 | 1613.061442 | 1.078698677 | 0.31275508 | 3.449020483 | 0.000562624 | 0.003259009 |
| lcl\|NC_000950.1_cds_NP_051281.1_933 | BB_R34 | 206.0163455 | 1.062124857 | 0.219253677 | 4.844273872 | 1.27E-06 | 1.27E-05 |
| lcl\|NC_001851.2_cds_NP_045458.1_1153 | BB_F25 | 156.0679977 | 1.059224925 | 0.435755106 | 2.430780295 | 0.015066347 | 0.04943736 |
| lcl\|NC_001318.1_cds_NP_212465.1_314 | BB_0331 | 360.8537922 | 1.056494139 | 0.349446428 | 3.023336497 | 0.002500041 | 0.011233693 |
| lcl\|NC_001318.1_cds_NP_212671.1_500 | BB_0537 | 1848.252264 | 1.055907202 | 0.23840089 | 4.429124408 | 9.46E-06 | 7.90E-05 |
| lcl\|NC_001318.1_cds_NP_212696.1_524 | BB_0562 | 1222.608908 | 1.053705746 | 0.14722859 | 7.156937018 | 8.25E-13 | 2.46E-11 |
| lcl\|NC_000948.1_cds_NP_051187.2_846 | BB_P26 | 172.8371938 | 1.044255165 | 0.268808755 | 3.884751315 | 0.000102435 | 0.000697785 |
| lcl\|NC_001318.1_cds_NP_212902.1_721 | BB_0768 | 1156.42566 | 1.034548687 | 0.234654542 | 4.408815955 | 1.04E-05 | 8.59E-05 |
| lcl\|NC_000949.1_cds_NP_051238.1_893 | BB_S35 | 265.7310316 | 1.022944148 | 0.241100842 | 4.242806206 | 2.21E-05 | 0.000171655 |
| lcl\|NC_000952.1_cds_NP_051365.1_1013 | BB_O32 | 381.8142121 | 1.017080674 | 0.245240622 | 4.147276529 | 3.36E-05 | 0.000251307 |
| lcl\|NC_000953.1_cds_NP_051417.1_1062 | erpO | 7072.829661 | 1.016942202 | 0.344623288 | 2.950880677 | 0.003168693 | 0.013359205 |
| lcl\|NC_000948.1_cds_NP_051200.1_859 | erpB | 7072.921801 | 1.016940813 | 0.344628397 | 2.950832906 | 0.003169183 | 0.013359205 |
| lcl\|NC_001857.2_cds_NP_045739.1_1334 | BB_A66 | 625.4863294 | 1.009382205 | 0.338610772 | 2.980951255 | 0.002873545 | 0.012388981 |
| lcl\|NC_001318.1_cds_NP_212585.1_426 | BB_0451 | 435.9912764 | 1.007584914 | 0.20928468 | 4.814422702 | 1.48E-06 | 1.44E-05 |
| lcl\|NC_001318.1_cds_NP_212510.1_357 | metK | 3414.267219 | 1.007169116 | 0.303249798 | 3.321252387 | 0.000896145 | 0.004796807 |
| lcl\|NC_000949.1_cds_NP_051229.2_886 | BB_S26 | 135.8581982 | 1.00586234 | 0.349072328 | 2.881529873 | 0.003957497 | 0.016318687 |
| lcl\|NC_001318.1_cds_YP_008686594.1_748 | BB_0794 | 8844.212411 | -1.00438858 | 0.174263856 | -5.763608144 | 8.23E-09 | 1.37E-07 |
| lcl\|NC_001318.1_cds_NP_212419.2_270 | BB_0285 | 4139.079468 | -1.005966128 | 0.317608 | -3.16731987 | 0.00153851 | 0.00755067 |
| lcl\|NC_001318.1_cds_NP_212599.1_440 | BB_0465 | 2885.545854 | -1.007502788 | 0.230478313 | -4.37135613 | 1.23E-05 | 0.000100134 |
| lcl\|NC_001852.1_cds_NP_045489.1_1181 | BB_G29 | 357.369219 | -1.009105265 | 0.377296002 | -2.674571849 | 0.007482474 | 0.028443423 |
| lcl\|NC_001318.1_cds_NP_212160.1_24 | BB_0026 | 1860.316044 | -1.010075684 | 0.181995954 | -5.549989787 | 2.86E-08 | 4.02E-07 |
| lcl\|NC_001318.1_cds_NP_212777.1_603 | ylqF | 699.0466979 | -1.012270773 | 0.224680411 | -4.505380641 | 6.63E-06 | 5.78E-05 |
| lcl\|NC_001318.1_cds_NP_212530.1_377 | rpmG | 7700.523929 | -1.014284963 | 0.225205091 | -4.503827867 | 6.67E-06 | 5.78E-05 |
| lcl\|NC_001318.1_cds_NP_212677.2_505 | BB_0543 | 8778.761317 | -1.019087913 | 0.163819468 | -6.220798579 | 4.95E-10 | 1.07E-08 |
| lcl\|NC_001318.1_cds_NP_212564.2_406 | BB_0430 | 199.0192104 | -1.022890971 | 0.324696532 | -3.150298416 | 0.001631038 | 0.007891072 |
| ncRNA0330 | p-(BB_Q04) | 720.3616324 | -1.024359045 | 0.398542035 | -2.570266009 | 0.010162045 | 0.036572852 |
| lcl\|NC_001318.1_cds_NP_212588.1_429 | BB_0454 | 2730.888853 | -1.031595834 | 0.18436356 | -5.595443224 | 2.20E-08 | 3.21E-07 |
| lcl\|NC_001856.1_cds_NP_045660.1_1272 | BB_J36 | 1399.444538 | -1.03853811 | 0.235436708 | -4.411113785 | 1.03E-05 | 8.54E-05 |
| lcl\|NC_001318.1_cds_NP_212829.1_654 | rpsP | 1323.098993 | -1.039395173 | 0.253757071 | -4.096024477 | 4.20E-05 | 0.000307202 |
| lcl\|NC_001318.1_cds_NP_212568.1_409 | BB_0434 | 410.6547229 | -1.042338737 | 0.306030686 | -3.405994191 | 0.000659236 | 0.003717479 |
| lcl\|NC_001318.1_cds_NP_212563.1_405 | BB_0429 | 2866.185843 | -1.045234832 | 0.205122092 | -5.095671663 | 3.48E-07 | 3.84E-06 |
| lcl\|NC_001318.1_cds_NP_212144.1_9 | BB_0010 | 909.552351 | -1.051581313 | 0.245404371 | -4.285096096 | 1.83E-05 | 0.00014536 |
| lcl\|NC_001318.1_cds_NP_212667.1_496 | BB_0533 | 5644.777969 | -1.062869351 | 0.106871435 | -9.945308127 | 2.64E-23 | 1.55E-21 |
| lcl\|NC_001318.1_cds_NP_212830.1_655 | BB_0696 | 1648.933208 | -1.064165262 | 0.332285958 | -3.202558632 | 0.001362126 | 0.006822647 |
| rna11 | tRNA-Ile-1 | 3677.251289 | -1.070763646 | 0.431861739 | -2.479413086 | 0.01315988 | 0.044643975 |
| ncRNA0083 | AI-(BB_0556,BB_0556/BB_0557) | 349.181935 | -1.071796374 | 0.268731567 | -3.988353087 | 6.65E-05 | 0.000470152 |
| ncRNA0001 | PI-(BB_0003,BB_0003/BB_0004) | 968.8208685 | -1.091121118 | 0.280770351 | -3.886169302 | 0.000101838 | 0.00069651 |
| lcl\|NC_001318.1_cds_NP_212732.1_560 | murB | 2605.560826 | -1.093452916 | 0.256548625 | -4.262166346 | 2.02E-05 | 0.000160363 |
| lcl\|NC_001318.1_cds_NP_212985.1_797 | BB_0852 | 258.4724191 | -1.108790124 | 0.362781795 | -3.056355468 | 0.002240455 | 0.010174651 |
| lcl\|NC_001318.1_cds_NP_212142.2_7 | cdaA | 904.7017596 | -1.110337438 | 0.151425193 | -7.332580652 | 2.26E-13 | 6.99E-12 |
| lcl\|NC_001318.1_cds_NP_212574.1_415 | rpmH | 1315.252463 | -1.117891999 | 0.181344863 | -6.164453644 | 7.07E-10 | 1.49E-08 |
| lcl\|NC_001318.1_cds_YP_008686584.1_588 | BB_0627 | 1365.69883 | -1.137219758 | 0.235678807 | -4.825294946 | 1.40E-06 | 1.38E-05 |
| lcl\|NC_001318.1_cds_NP_212562.1_404 | BB_0428 | 2667.6549 | -1.138481301 | 0.213578724 | -5.330499604 | 9.79E-08 | 1.21E-06 |
| lcl\|NC_001853.1_cds_NP_045517.1_1197 | BB_H26 | 1195.446691 | -1.144943567 | 0.461056964 | -2.483301754 | 0.013017077 | 0.044247668 |
| lcl\|NC_001318.1_cds_NP_212138.2_3 | BB_0004 | 3213.612708 | -1.161277569 | 0.350246068 | -3.315604869 | 0.00091445 | 0.004806509 |
| ncRNA0014 | A-(BB_0084) | 380.5460997 | -1.168956757 | 0.38865126 | -3.007726663 | 0.002632098 | 0.01162608 |
| ncRNA0076 | AA-(BB_0465,BB_0466) | 1466.200068 | -1.173752479 | 0.258622838 | -4.538471893 | 5.67E-06 | 5.00E-05 |
| ncRNA0117 | A-(BB_0747) | 270.2800642 | -1.177533684 | 0.473249509 | -2.488187864 | 0.012839589 | 0.043805776 |
| lcl\|NC_001856.1_cds_NP_045643.1_1260 | BB_J19 | 8721.994493 | -1.178524274 | 0.357433879 | -3.297181218 | 0.000976605 | 0.005055191 |
| lcl\|NC_001849.2_cds_NP_045399.2_1117 | BB_D15 | 1008.771523 | -1.197583135 | 0.198847084 | -6.022633637 | 1.72E-09 | 3.32E-08 |
| lcl\|NC_001318.1_cds_NP_212477.1_326 | gatC | 285.3009861 | -1.21149949 | 0.216711395 | -5.590382031 | 2.27E-08 | 3.24E-07 |
| lcl\|NC_001318.1_cds_YP_008686588.1_680 | cabP | 3690.875669 | -1.213430799 | 0.267574041 | -4.534934676 | 5.76E-06 | 5.06E-05 |
| lcl\|NC_001856.1_cds_NP_045642.1_1259 | BB_J18 | 652.4014099 | -1.21353758 | 0.493482874 | -2.459128057 | 0.013927493 | 0.046598272 |
| ncRNA0061 | A-(BB_0374) | 321.0807142 | -1.221514521 | 0.292759095 | -4.172422107 | 3.01E-05 | 0.0002261 |
| lcl\|NC_001855.1_cds_NP_045624.1_1252 | BB_K53 | 359.8395733 | -1.240359481 | 0.484313307 | -2.561068345 | 0.010435083 | 0.037333919 |
| lcl\|NC_001318.1_cds_NP_212561.2_403 | BB_0427 | 376.5915461 | -1.245177776 | 0.398443638 | -3.12510392 | 0.001777423 | 0.00850267 |
| ncRNA0031 | AA-(BB_0198,BB_0199) | 400.7134034 | -1.254495741 | 0.373991555 | -3.354342427 | 0.000795538 | 0.004384473 |
| lcl\|NC_001855.1_cds_NP_045612.1_1242 | BB_K40 | 3811.74482 | -1.258348118 | 0.231710329 | -5.430694971 | 5.61E-08 | 7.35E-07 |
| lcl\|NC_001318.1_cds_NP_212306.2_161 | BB_0172 | 2887.764892 | -1.261054254 | 0.123970769 | -10.17219033 | 2.64E-24 | 1.61E-22 |
| ncRNA0286 | A-(BB_K19) | 219.9417934 | -1.262032589 | 0.497593607 | -2.536271711 | 0.011203977 | 0.039528405 |
| lcl\|NC_000949.1_cds_NP_051234.2_890 | BB_S31 | 105.7674492 | -1.268266915 | 0.504053967 | -2.516133186 | 0.011865034 | 0.041491072 |
| lcl\|NC_001318.1_cds_NP_212174.1_38 | cheR | 2192.918259 | -1.271798562 | 0.187794352 | -6.772293995 | 1.27E-11 | 3.32E-10 |
| lcl\|NC_001318.1_cds_NP_212157.1_21 | ruvA | 1097.97296 | -1.273090208 | 0.151883284 | -8.382029754 | 5.20E-17 | 2.39E-15 |
| lcl\|NC_001318.1_cds_NP_212932.1_752 | BB_0798 | 412.7429471 | -1.27472152 | 0.230756119 | -5.524107118 | 3.31E-08 | 4.62E-07 |
| lcl\|NC_001318.1_cds_NP_212549.1_394 | BB_0415 | 1448.604328 | -1.279087526 | 0.222055031 | -5.760227649 | 8.40E-09 | 1.39E-07 |
| lcl\|NC_001903.1_cds_NP_046996.1_806 | BB_B10 | 3773.691125 | -1.28185313 | 0.381829657 | -3.357133493 | 0.000787551 | 0.004368726 |
| lcl\|NC_001857.2_cds_NP_045697.1_1303 | dbpA | 424.4061803 | -1.294172188 | 0.532360559 | -2.431006891 | 0.015056928 | 0.04943736 |
| lcl\|NC_001318.1_cds_NP_212817.1_643 | BB_0683 | 9215.747161 | -1.342614394 | 0.200226812 | -6.705467576 | 2.01E-11 | 5.18E-10 |
| ncRNA0229 | I-(BB_D18/BB_D20) | 117.8530042 | -1.351834939 | 0.345411029 | -3.913699406 | 9.09E-05 | 0.000631798 |
| lcl\|NC_001318.1_cds_NP_212818.2_644 | fni | 5419.350534 | -1.360464659 | 0.310958576 | -4.375067179 | 1.21E-05 | 9.89E-05 |
| ncRNA0110 | A-(BB_0697) | 1608.781804 | -1.363019634 | 0.256463472 | -5.314673554 | 1.07E-07 | 1.30E-06 |
| ncRNA0035 | A-(BB_0208) | 510.9105757 | -1.367867121 | 0.292134086 | -4.682326331 | 2.84E-06 | 2.64E-05 |
| ncRNA0281 | IpI-(BB_K09/BB_K10,BB_K10,BB_K10/BB_K12) | 166.2483876 | -1.374538803 | 0.505265076 | -2.720431054 | 0.006519687 | 0.025291632 |
| ncRNA0304 | I-(BB_J37/BB_J41) | 103.2796554 | -1.378344172 | 0.48298017 | -2.853831809 | 0.004319539 | 0.017514704 |
| lcl\|NC_001855.1_cds_NP_045606.1_1238 | BB_K33 | 37.62541137 | -1.381941183 | 0.444818197 | -3.106755056 | 0.001891531 | 0.008898554 |
| lcl\|NC_001856.1_cds_NP_045669.1_1277 | BB_J45 | 396.4427063 | -1.389596423 | 0.387960567 | -3.5817981 | 0.000341237 | 0.002120903 |
| lcl\|NC_001849.2_cds_NP_045405.1_1120 | BB_D22 | 128.5625749 | -1.396085804 | 0.318107631 | -4.388721519 | 1.14E-05 | 9.38E-05 |
| ncRNA0003 | AA-(BB_0005,BB_0006) | 1243.576562 | -1.402633207 | 0.311544968 | -4.5021854 | 6.73E-06 | 5.78E-05 |
| lcl\|NC_000950.1_cds_NP_051274.2_927 | bdrH | 752.05999 | -1.407429246 | 0.239268756 | -5.882210729 | 4.05E-09 | 7.11E-08 |
| lcl\|NC_001318.1_cds_YP_008686589.1_694 | BB_0739 | 1873.957841 | -1.419653077 | 0.173023273 | -8.204983358 | 2.31E-16 | 1.01E-14 |
| lcl\|NC_001855.1_cds_NP_045597.1_1234 | BB_K23 | 2453.836139 | -1.425251086 | 0.386783132 | -3.684884288 | 0.000228807 | 0.001481588 |
| ncRNA0245 | I-(BB_E31/BB_E33) | 314.7614859 | -1.445505713 | 0.484755182 | -2.98192937 | 0.002864381 | 0.012380813 |
| lcl\|NC_001856.1_cds_NP_045648.1_1264 | BB_J24 | 96.33456458 | -1.446657884 | 0.37915883 | -3.815440313 | 0.00013594 | 0.000911442 |
| lcl\|NC_001849.2_cds_NP_045397.1_1115 | BB_D13 | 908.2099786 | -1.446791877 | 0.304769578 | -4.747166331 | 2.06E-06 | 1.94E-05 |
| lcl\|NC_001852.1_cds_NP_045484.2_1176 | BB_G24 | 50.67291727 | -1.450408152 | 0.561382788 | -2.583634878 | 0.009776523 | 0.035651861 |
| lcl\|NC_001855.1_cds_NP_045598.1_1235 | BB_K24 | 377.5769784 | -1.452422742 | 0.43695597 | -3.323956741 | 0.0008875 | 0.004792717 |
| ncRNA0002 | AI-(BB_0004,BB_0004/BB_0005) | 3041.858416 | -1.466630978 | 0.248955372 | -5.891140104 | 3.84E-09 | 6.87E-08 |
| ncRNA0154 | A-(BB_S11) | 104.5257947 | -1.477242221 | 0.477036608 | -3.096706199 | 0.001956837 | 0.00913012 |
| lcl\|NC_001852.1_cds_NP_045491.2_1183 | BB_G31 | 41.00949741 | -1.490324525 | 0.469590481 | -3.173668518 | 0.001505255 | 0.007430287 |
| ncRNA0007 | A-(BB_0014) | 214.8526993 | -1.496435951 | 0.482793757 | -3.099534591 | 0.001938249 | 0.00909322 |
| ncRNA0063 | A-(BB_0381) | 391.0552137 | -1.499645362 | 0.433017783 | -3.463241977 | 0.000533708 | 0.003123385 |
| ncRNA0037 | A-(BB_0211) | 1238.76926 | -1.499844735 | 0.314759246 | -4.76505378 | 1.89E-06 | 1.81E-05 |
| lcl\|NC_001857.2_cds_NP_045696.2_1302 | BB_A23 | 697.3262104 | -1.509462463 | 0.587335071 | -2.570019291 | 0.010169285 | 0.036572852 |
| lcl\|NC_000948.1_cds_NP_051176.1_835 | BB_P15 | 57.75190122 | -1.511845915 | 0.533207038 | -2.83538252 | 0.004577085 | 0.018471031 |
| lcl\|NC_000953.1_cds_NP_051392.1_1039 | BB_L15 | 57.75190122 | -1.511845915 | 0.533207038 | -2.83538252 | 0.004577085 | 0.018471031 |
| lcl\|NC_001318.1_cds_NP_212711.1_539 | BB_0577 | 1867.115855 | -1.516846079 | 0.22325663 | -6.794181569 | 1.09E-11 | 2.90E-10 |
| ncRNA0125 | AIA-(BB_0794,BB_0794/BB_0795,BB_0795) | 2242.036294 | -1.573260538 | 0.260394058 | -6.041844998 | 1.52E-09 | 2.98E-08 |
| lcl\|NC_001318.1_cds_NP_212156.1_20 | ruvB | 2074.85845 | -1.615201563 | 0.202585957 | -7.972919689 | 1.55E-15 | 5.86E-14 |
| ncRNA0299 | I-(BB_J20/BB_J0058) | 119.1266624 | -1.620207512 | 0.322869489 | -5.018149959 | 5.22E-07 | 5.62E-06 |
| lcl\|NC_001318.1_cds_NP_212760.1_587 | rnmV | 17091.35904 | -1.622284749 | 0.410087902 | -3.955943936 | 7.62E-05 | 0.000536466 |
| ncRNA0133 | A-(BB_B03) | 471.6283693 | -1.626316186 | 0.246864331 | -6.587894579 | 4.46E-11 | 1.10E-09 |
| ncRNA0318 | IP-(BB_A16/BB_A18,BB_A18) | 1800.37672 | -1.63311484 | 0.56659464 | -2.882333724 | 0.003947414 | 0.016316617 |

| ncRNA0242 | I-(BB_E23b/BB_E29a) | 107.0500482 | -1.634381518 | 0.591753121 | -2.761931385 | 0.005746055 | 0.022704251 |
| --- | --- | --- | --- | --- | --- | --- | --- |
| ncRNA0006 | A-(BB_0013) | 3033.894978 | -1.651261635 | 0.339717919 | -4.860684529 | 1.17E-06 | 1.18E-05 |
| lcl\|NC_001856.1_cds_NP_045667.1_1276 | BB_J43 | 43.39693922 | -1.683539357 | 0.669523881 | -2.514532201 | 0.011919042 | 0.041513166 |
| lcl\|NC_001857.2_cds_NP_045707.1_1309 | BB_A34 | 190.0838372 | -1.75905884 | 0.577174012 | -3.047709707 | 0.002305926 | 0.010416423 |
| ncRNA0057 | A-(BB_0347) | 34.51259723 | -1.771846181 | 0.548621557 | -3.22963281 | 0.001239493 | 0.006263669 |
| lcl\|NC_000948.1_cds_NP_051171.1_830 | BB_P10 | 89.29313498 | -1.80856563 | 0.568945229 | -3.178804458 | 0.001478838 | 0.007321109 |
| lcl\|NC_000953.1_cds_NP_051387.1_1034 | BB_L10 | 89.29313498 | -1.80856563 | 0.568945229 | -3.178804458 | 0.001478838 | 0.007321109 |
| lcl\|NC_001856.1_cds_NP_045661.2_1273 | BB_J37 | 15.94581387 | -1.809580589 | 0.694226935 | -2.606612476 | 0.009144279 | 0.033489691 |
| ncRNA0322 | I-(BB_A37/BB_A38) | 2507.453992 | -1.827061965 | 0.464565418 | -3.932841091 | 8.39E-05 | 0.000588325 |
| ncRNA0205 | A-(BB_L36) | 27.97464525 | -1.847277027 | 0.738413586 | -2.501683421 | 0.01236044 | 0.042697421 |
| lcl\|NC_001849.2_cds_NP_045404.1_1119 | BB_D21 | 967.6859329 | -1.852887349 | 0.440068742 | -4.210449803 | 2.55E-05 | 0.000194633 |
| lcl\|NC_001857.2_cds_NP_045710.1_1311 | BB_A37 | 93.94411642 | -1.86769252 | 0.652655129 | -2.861683662 | 0.004213973 | 0.017127436 |
| ncRNA0042 | A-(BB_0240) | 2021.458735 | -1.868463425 | 0.349137347 | -5.351657282 | 8.72E-08 | 1.10E-06 |
| ncRNA0148 | A-(BB_P21) | 93.09625772 | -1.906327102 | 0.42831528 | -4.450756704 | 8.56E-06 | 7.25E-05 |
| ncRNA0080 | p-(BB_0522) | 879.8130905 | -1.911466532 | 0.236932979 | -8.067541052 | 7.17E-16 | 2.81E-14 |
| ncRNA0188 | A-(BB_O36) | 23.57876286 | -1.934664257 | 0.731373306 | -2.64524866 | 0.008163091 | 0.030756069 |
| ncRNA0191 | A-(BB_O44) | 8.708649435 | -1.952083338 | 0.79304848 | -2.461493072 | 0.013836007 | 0.0464051 |
| lcl\|NC_001849.2_cds_NP_045398.1_1116 | BB_D14 | 3487.083186 | -1.95219011 | 0.326963037 | -5.970675236 | 2.36E-09 | 4.39E-08 |
| ncRNA0050 | AIA-(BB_0269,BB_0269/BB_0270,BB_0270) | 1285.298201 | -1.970983739 | 0.556213454 | -3.543574368 | 0.000394742 | 0.002392333 |
| ncRNA0070 | A-(BB_0446) | 529.3172145 | -2.031253639 | 0.313402561 | -6.481292412 | 9.09E-11 | 2.12E-09 |
| lcl\|NC_001318.1_cds_NP_212161.1_25 | BB_0027 | 9710.085949 | -2.082180896 | 0.337466351 | -6.170040036 | 6.83E-10 | 1.45E-08 |
| ncRNA0136 | AI-(BB_B09,BB_B09/BB_B10) | 476.369844 | -2.090544907 | 0.354339634 | -5.899833676 | 3.64E-09 | 6.59E-08 |
| lcl\|NC_001852.1_cds_NP_045485.1_1177 | BB_G25 | 3.961622236 | -2.090912702 | 0.854820598 | -2.446025174 | 0.014444091 | 0.047594109 |
| ncRNA0284 | A-(BB_K17) | 123.8271122 | -2.104304342 | 0.296306341 | -7.101786398 | 1.23E-12 | 3.62E-11 |
| lcl\|NC_001853.1_cds_NP_045510.1_1195 | BB_H17 | 10.29555978 | -2.148674895 | 0.800721139 | -2.683424715 | 0.007287238 | 0.027825485 |
| ncRNA0043 | A-(BB_0244) | 577.795707 | -2.193831378 | 0.40473779 | -5.420376925 | 5.95E-08 | 7.73E-07 |
| lcl\|NC_001852.1_cds_NP_045472.1_1163 | BB_G12 | 81.95387397 | -2.20532043 | 0.670605133 | -3.288552864 | 0.001007039 | 0.00519693 |
| ncRNA0225 | I-(BB_D04/BB_D05a) | 86.57888532 | -2.205735552 | 0.542257921 | -4.067687105 | 4.75E-05 | 0.00034119 |
| ncRNA0087 | A-(BB_0588) | 1536.156421 | -2.232166273 | 0.709217961 | -3.147362865 | 0.001647504 | 0.007948155 |
| ncRNA0200 | IA-(BB_L29/BB_L30,BB_L30) | 867.0447963 | -2.254648069 | 0.353335763 | -6.381035557 | 1.76E-10 | 3.89E-09 |
| ncRNA0132 | pI-(BB_0845a,BB_0845a/BB_0845b) | 211.2546476 | -2.25854887 | 0.535976359 | -4.213896437 | 2.51E-05 | 0.000192548 |
| lcl\|NC_001318.1_cds_NP_212722.1_550 | BB_0588 | 1645.236707 | -2.280846585 | 0.1324333 | -17.2226063 | 1.80E-66 | 5.10E-64 |
| lcl\|NC_001903.1_cds_NP_047015.1_821 | BB_B29 | 24118.646 | -2.302950375 | 0.212949666 | -10.81452915 | 2.94E-27 | 2.18E-25 |
| ncRNA0239 | A-(BB_E09) | 1464.223554 | -2.309739158 | 0.428228105 | -5.393712208 | 6.90E-08 | 8.84E-07 |
| lcl\|NC_001849.2_cds_YP_004940417.1_112 | BB_D0031 | 25.13306541 | -2.332828324 | 0.700631412 | -3.329608525 | 0.000869682 | 0.004731846 |
| ncRNA0105 | A-(BB_0660) | 99.2736016 | -2.451368375 | 0.757036542 | -3.23811103 | 0.00120324 | 0.006116768 |
| ncRNA0233 | p-(BB_D23) | 46.44975772 | -2.519658462 | 0.750954393 | -3.355274949 | 0.000792861 | 0.004383906 |
| ncRNA0084 | A-(BB_0581) | 87.52936081 | -2.554026659 | 0.470127152 | -5.43262955 | 5.55E-08 | 7.33E-07 |
| ncRNA0240 | A-(BB_E09) | 74.83749 | -2.662732492 | 0.853298616 | -3.120516594 | 0.001805341 | 0.008612034 |
| lcl\|NC_001318.1_cds_NP_212643.1_482 | BB_0509 | 8232.921912 | -2.709765829 | 0.365717385 | -7.409453136 | 1.27E-13 | 4.15E-12 |
| lcl\|NC_001318.1_cds_NP_212828.2_653 | ffh | 14060.76269 | -3.018553552 | 0.374236073 | -8.065907513 | 7.27E-16 | 2.81E-14 |
| lcl\|NC_001852.1_cds_NP_045482.1_1174 | BB_G22 | 64.42227065 | -3.125608056 | 0.641815831 | -4.869945401 | 1.12E-06 | 1.13E-05 |
| ncRNA0257 | PI-(BB_G05,BB_G05/BB_G06) | 56.23393737 | -3.935288751 | 0.795248019 | -4.948504938 | 7.48E-07 | 8.01E-06 |
| ncRNA0297 | A-(BB_J18) | 206.5236032 | -4.034115008 | 0.717156855 | -5.625150172 | 1.85E-08 | 2.74E-07 |
| ncRNA0226 | P-(BB_D05a) | 56.8286166 | -4.172467124 | 0.631313043 | -6.60918885 | 3.86E-11 | 9.68E-10 |
| ncRNA0071 | A-(BB_0450) | 65.28090439 | -4.497370774 | 0.703386199 | -6.39388544 | 1.62E-10 | 3.67E-09 |
| ncRNA0099 | A-(BB_0633) | 150.8640122 | -4.912156619 | 0.640176561 | -7.673127875 | 1.68E-14 | 6.08E-13 |
| ncRNA0308 | I-(BB_J50/BB_J51) | 893.058206 | -5.20269521 | 0.494508761 | -10.52093637 | 6.92E-26 | 4.91E-24 |
